# Supplementary material for: Glutamyl-prolyl-tRNA synthetase 1 coordinates early endosomal anti-inflammatory AKT signaling
Source: Nat Commun. 2022 Oct 29;13:6455. doi: 10.1038/s41467-022-34226-4 (PMC9617928; doi:10.1038/s41467-022-34226-4)
Supplement: Supplementary file 1 — Supplementary Information [file 41467_2022_34226_MOESM1_ESM.pdf]

Supplementary Information

Glutamyl-prolyl-tRNA synthetase 1 coordinates early endosomal anti-inflammatory AKT signaling

Eun-Young Lee, Su-Man Kim, Jung Hwan Hwang, Song Yee Jang, Shinhye Park, Sanghyeon Choi, Ga Seul Lee, Jungwon Hwang, Jeong Hee Moon, Paul L Fox, Sunghoon Kim, Chul-Ho Lee, and Myung Hee Kim

Supplementary Figures 1–8 and Legends
Supplementary Table 1
Supplementary Table 2

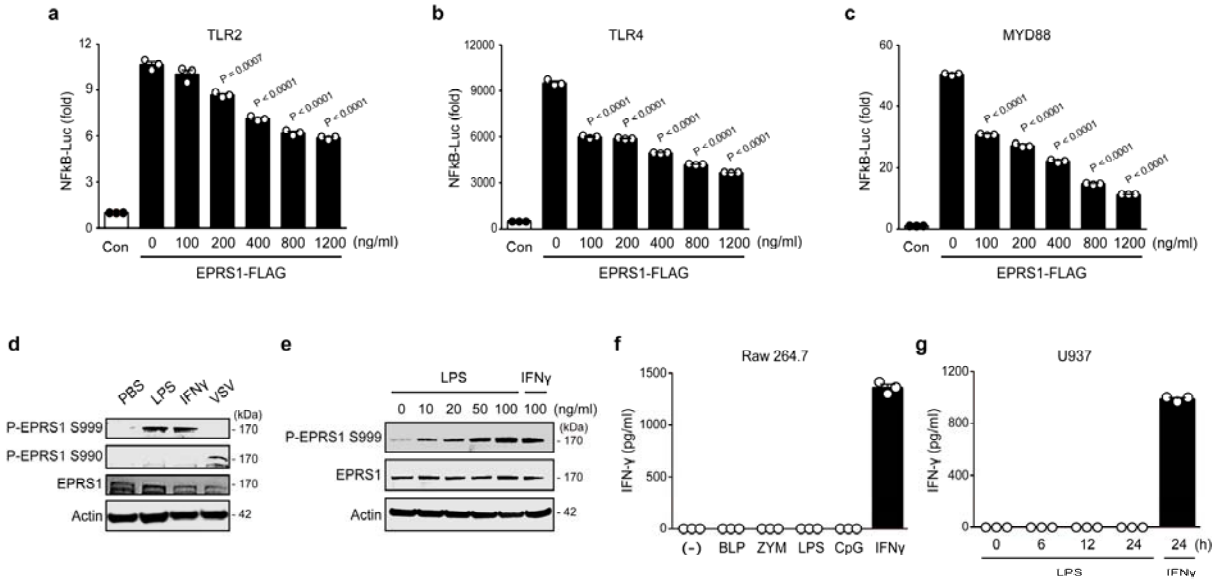

Supplementary Fig. 1. Phosphorylation of EPRS1 by diverse stimuli.

a, b Luciferase assay of NF-κB reporter activation in 293/TLR2 cells (**a**) or 293/TLR4 cells (**b**) transfected for 24 h with increasing concentrations (DNA doses: 0, 100, 200, 400, 800, 1200 ng/ml) of EPRS1-FLAG plasmids, followed by stimulation for 6 h with BLP (**a**, 100 ng/ml) or LPS (**b**, 100 ng/ml), respectively. **c** Luciferase assay of NF-κB reporter activation in 293T cells at 24 h after transfection of a plasmid encoding the TLR signaling protein MYD88, along with increasing concentrations (DNA doses: 0, 100, 200, 400, 800, 1200 ng/ml) of a plasmid encoding FLAG-tagged EPRS1. Data are expressed as the mean ± SEM. Two-tailed unpaired *t*-tests were used. **d** Immunoblot analysis of EPRS1 phosphorylated at Ser999 and Ser990 in RAW 264.7 cells treated with LPS (100 ng/ml) or IFNγ (100 ng/ml), or infected with VSV-GFP virus (1 MOI). **e** Dose-dependent analysis of LPS-mediated (10–100 ng/ml) stimulation of EPRS1 Ser999 phosphorylation. IFNγ (100 ng/ml) was used as a positive control. **f** Amounts of IFNγ secreted from RAW 264.7 cells treated with BLP (100 ng/ml), ZYM (10 μg/ml), LPS (100 ng/ml), or CpG (1 μg/ml). IFNγ (100 ng/ml) was used as a positive

control. **g** Amounts of IFNγ secreted from U937 cells treated with LPS (100 ng/ml) for the indicated times. IFNγ (100 ng/ml) was used as a positive control. Data are representative of two independent experiments, each with similar results. Source data are provided as a Source Data file.

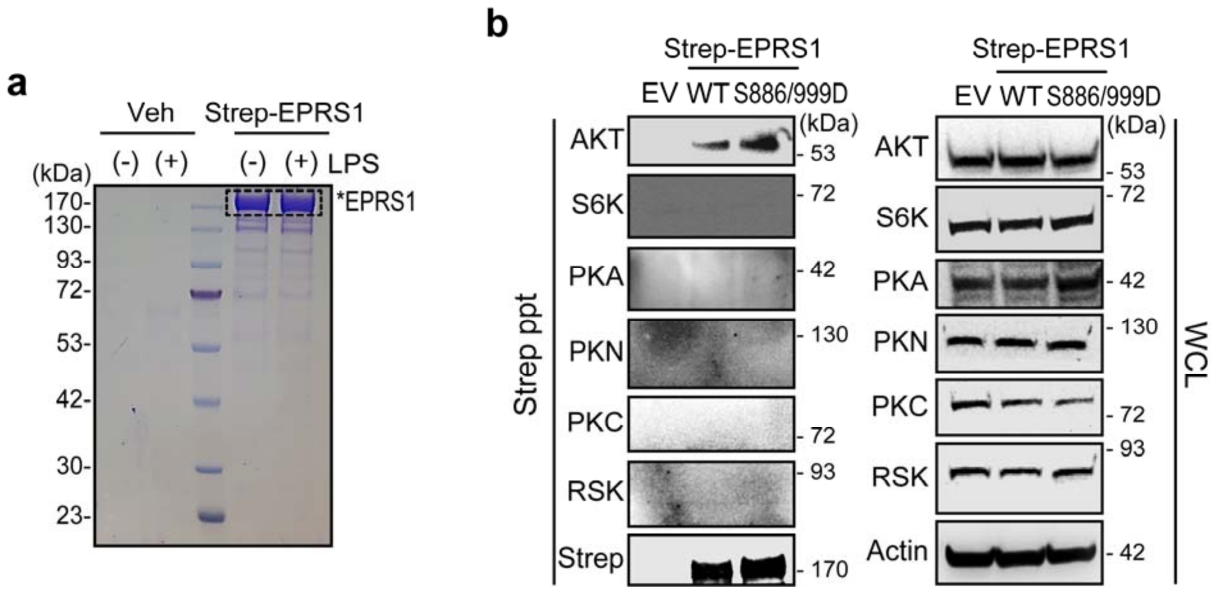

Supplementary Fig. 2. Interactome analysis of EPRS1.

a CBB-stained Strep-EPRS1 (indicated by a dotted box) purified by Strep-precipitation of U937 cells treated or untreated for 1 h with LPS (100 ng/ml). Veh, vehicle. **b** Strep-tagged empty vector (EV), Strep-EPRS1 WT, or phosphomimetic (S886D/S999D) Strep-EPRS1 was transfected into 293/hTLR4 cells, stimulated with LPS (100 ng/ml) and then pulled down with Strep-Tactin beads. Precipitates of endogenous kinases were analyzed by immunoblotting with the indicated antibodies. Data are representative of two independent experiments, each with similar results. Source data are provided as a Source Data file.

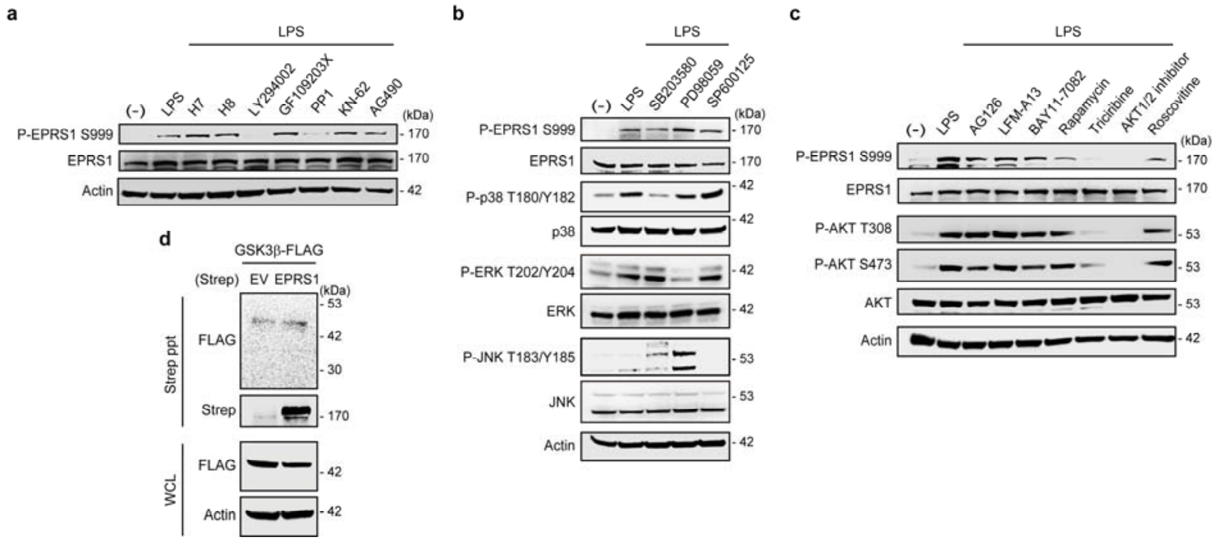

Supplementary Fig. 3. Identification of kinases responsible for LPS-triggered phosphorylation of EPRS1 Ser999.

a Immunoblot analysis of EPRS1 Ser999 phosphorylation in U937 cells treated with LPS (100 ng/ml) for 1 h. For pharmacological inhibition, 2% serum-starved cells were pretreated with the indicated kinase inhibitors for 30 min before LPS stimulation. The kinase inhibitors used were H7 (PKA/PKG/PKC, 10 μM), H8 (PKA/PKG, 10 μM), LY294002 (PI3K, 5 μM), GF109203X (PKC, 20 nM), PP1 (Src family, 10 μM), KN-62 (CAMKII, 5 μM), and AG490 (JAK2, 10 μM). **b** Immunoblot analysis of EPRS1 Ser999 and MAP kinase (p38, ERK, and JNK) phosphorylation in U937 cells treated with LPS (100 ng/ml) for 1 h. MAPK inhibitors SB203580 (p38, 10 μM), PD98059 (ERK, 10 μM), and SP600125 (JNK, 10 μM) were administered for 30 min before the stimuli. **c** Immunoblot analysis of EPRS1 Ser999 phosphorylation, along with AKT Thr308 and Ser473 phosphorylation, in U937 cells treated with LPS (100 ng/ml) for 1 h. Kinase inhibitors AG126 (IRAK, 30 μM), LFM-A13 (BTK, 10 μM), BAY11-7082 (IKK, 2 μM), rapamycin (mTOR, 10 nM), triciribine (AKT, 10 μM), an AKT1/2 inhibitor (AKT, 2.5 μM), and roscovitine (Cdk5, 10 μM) were administered for 30 min before stimulation. **d**

Immunoassay of the interaction between EPRS1 and GSK3β in lysates of 293T cells co-expressing Strep-EPRS1 and GSK3β-FLAG, assessed after precipitation with a Strep-tagged protein. Data are representative of three independent experiments, each with similar results. Source data are provided as a Source Data file.

a

Gene Symbol	Description	Protein ID
RAB5B	Ras-related protein Rab-5B	P61020
RAB5C	Ras-related protein Rab-5C	P51148
AKT1	RAC-alpha serine/threonine-protein kinase	P31749
ARL1	ADP-ribosylation factor-like protein 1	P40616
ARF4	ADP-ribosylation factor 4	P18085
YWHAG	14-3-3 protein gamma	P61981
CDK1	Cyclin-dependent kinase 1	P06493
COL1A1	Collagen alpha-1(I) chain	P02452
RCC2	Protein RCC2	Q9P258
MSH2	DNA mismatch repair protein Msh2	P54709
PHB2	Prohibitin-2	Q99623
ATP1B3	Sodium/potassium-transporting ATPase subunit beta-3	P54709
XPOT	Exportin-T	O43592
XPO1	Exportin-1	O14980
CSE1L	Exportin-2	P55060
XPO7	Exportin-7	Q9UIA9
IPO5	Importin-5	O00410
KPNB1	Importin subunit beta-1	Q14974
TMED10	Transmembrane emp24 domain-containing protein 10	P49755
COPB1	Coatomer subunit beta	P53618
PDCD6	Programmed cell death protein 6	O75340
RUVBL2	RuvB-like 2	Q9Y230
FAF2	FAS-associated factor 2	Q96CS3
TOR1AIP1	Torsin-1A-interacting protein 1	Q5JTV8
NSF	Vesicle-fusing ATPase	P46459
CLTA	Clathrin light chain A	P09496
SRP68	Signal recognition particle subunit SRP68	Q9UHB9

b

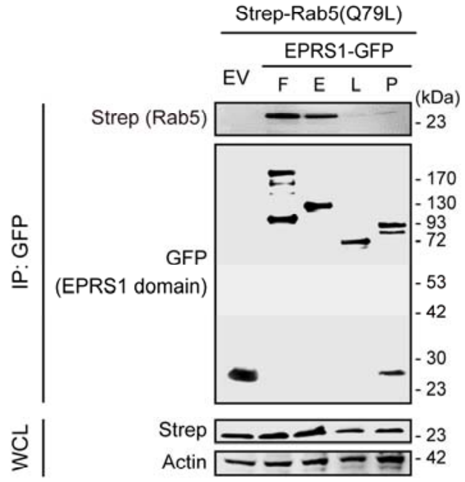

c

d

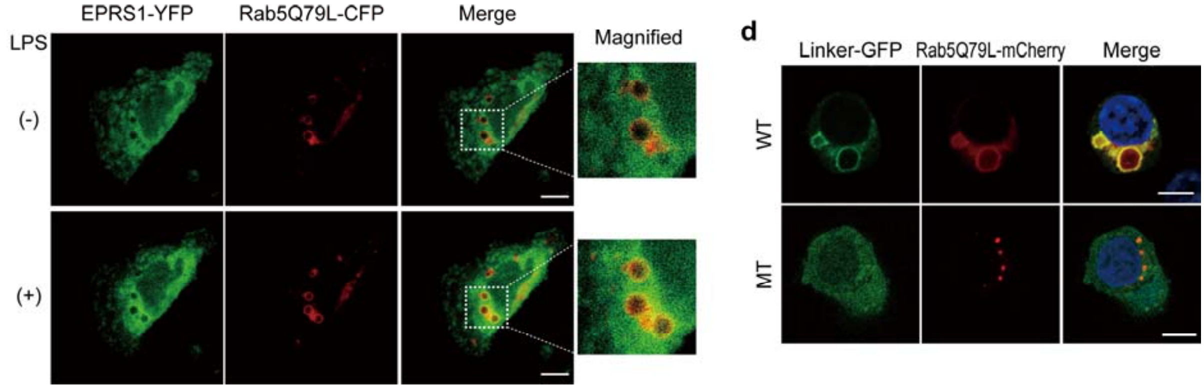

Supplementary Fig. 4. EPRS1 interacts specifically with Rab5.

a Table of EPRS1-interacting proteins identified by LC-MS/MS analysis. PANTHER classification of EPRS1-interacting proteins associated with cellular localization. **b** Immunoassay of the interaction between EPRS1 and Rab5 in lysates of 293T cells co-expressing full-length EPRS1-GFP (or its domains) and Strep-Rab5(Q79L); the interaction was assessed by co-immunoprecipitation with an anti-GFP antibody, followed by immunoblot analysis with an anti-Strep antibody. **c** Confocal live imaging of colocalization between EPRS1-YFP (green) and Rab5(Q79L)-CFP (red) transfected

into HeLa cells, followed by LPS (500 ng/ml) stimulation (up to 30 min). Far right, magnified areas shown within the white dotted boxes on the left. Scale bars, 10 μm. **d** Confocal microscopy images of colocalization between the EPRS1 linker domain (GFP, green) and active Rab5 (mCherry, red) after transfection into 293T cells. WT, wild-type. MT, K974A/K977A/P978A/K980A mutant. Scale bars, 5 μm. Data are representative of two independent experiments, each with similar results. Source data are provided as a Source Data file.

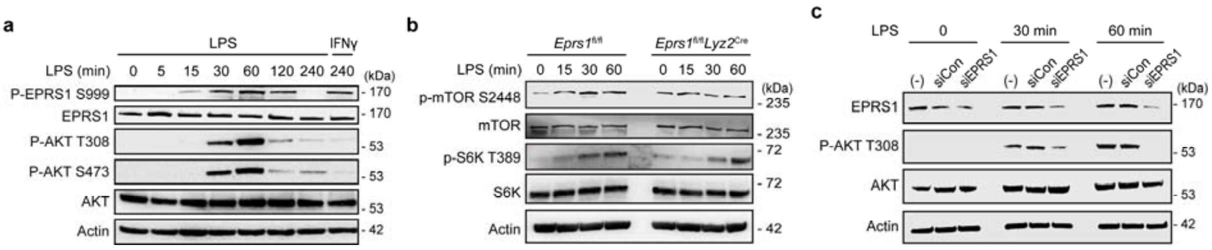

Supplementary Fig. 5. Cell signaling pathways regulated by EPRS1.

a Immunoblot analysis of phosphorylated- and total EPRS1 and AKT, along with actin, in BMDMs stimulated with LPS (100 ng/ml) for the indicated periods. IFNγ (100 ng/ml) was used as a positive control to compare EPRS1 and AKT activation against LPS stimuli. **b** Immunoblot analysis of phosphorylated- and total mTOR and S6K, along with actin, in LPS-treated BMDMs isolated from *Eprs1*$^{fl/fl}$ and *Eprs1*$^{fl/fl}$*Lyz2*^{Cre} mice. **c** Western blot analysis (using the indicated antibodies) of cell lysates from LPS-treated RAW 264.7 cells transfected with EPRS1-specific siRNA (siEPRS1) or non-targeting siRNA (siCon). Data are representative of two independent experiments, each with similar results. Source data are provided as a Source Data file.

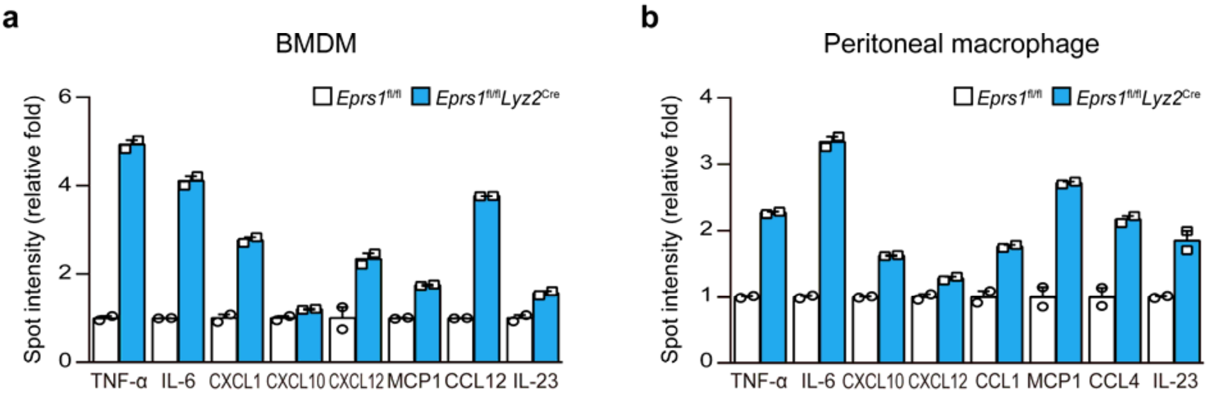

Supplementary Fig. 6. EPRS1-deficient macrophages show increased production of pro-inflammatory cytokines.

a, b Mouse cytokine arrays were used to detect cytokines and chemokines in culture supernatants from *Eprs1*$^{fl/fl}$ and *Eprs1*$^{fl/fl}$*Lyz2*^{Cre} mouse-derived BMDMs (**a**) and peritoneal macrophages (**b**) treated with LPS (100 ng/ml) for 12 h. Experiments were repeated twice. The mean pixel density of key cytokines and chemokines was analyzed using ImageJ software. Source data are provided as a Source Data file.

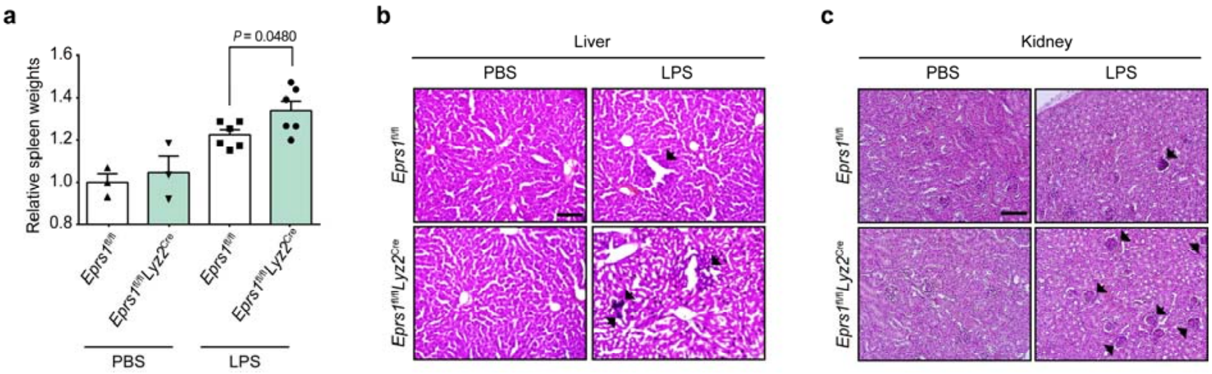

Supplementary Fig. 7. Inflammatory symptoms in LPS-challenged EPRS1-deficient mice.

Mice received an intraperitoneal injection of PBS or LPS (30 mg/kg) and were sacrificed at 24 h for experimental analysis. **a** Spleens were isolated from each group (n = 3 for each PBS group; n = 6 for the LPS-treated *Eprs1*$^{fl/fl}$ and *Eprs1*$^{fl/fl}$*Lyz2*^{Cre} groups) and relative weight was represented as a bar graph. Data are expressed as the mean ± SEM. Two-tailed unpaired *t*-tests were used. Source data are provided as a Source Data file. **b, c** H&E-stained sections of liver (**b**) and kidney (**c**) tissue from *Eprs1*$^{fl/fl}$ and *Eprs1*$^{fl/fl}$*Lyz2*^{Cre} mice 24 h after LPS injection. Scale bars, 200 μm. Black arrows indicate infiltration by inflammatory cells and sites of damage. Data shown are representative of two independent experiments, each with similar results. Source data are provided as a Source Data file.

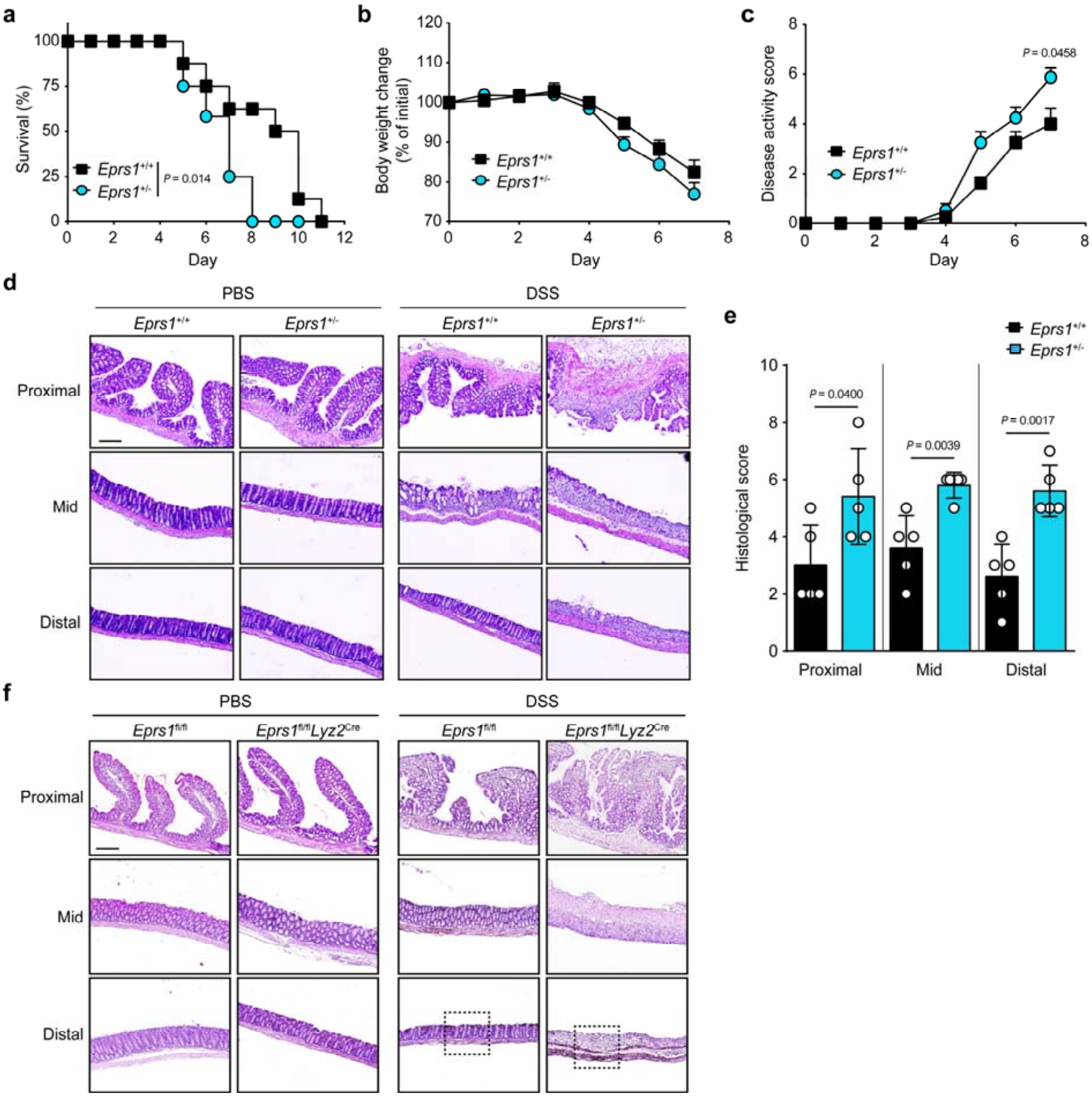

Supplementary Fig. 8. EPRS1 protects mice from DSS-induced colitis.

a Survival rates (presented as percentages) of *Eprs1*$^{+/+}$ (n = 8) and *Eprs1*$^{+/-}$ (n = 12) mice after administration of 5% DSS. **b, c** Body weight (**b**) and disease activity score (**c**) of *Eprs1*$^{+/+}$ and *Eprs1*$^{+/-}$ (n = 4 per group) mice were monitored for 7 days after treatment with 2% DSS. **d, e** Representative H&E-stained images (**d**) and histological scores (**e**) of colon tissues from *Eprs1*$^{+/+}$ and *Eprs1*$^{+/-}$ (n = 5 per group) mice on Day

7. Scale bar, 200 μm. Data are expressed as the mean ± SD. **f** Representative H&E staining in the proximal, mid, and distal colon from *Eprs1*$^{fl/fl}$ and *Eprs1*$^{fl/fl}$*Lyz2*^{Cre} mice on Day 8 post-treatment with 2% DSS. Magnified images of the boxed areas are shown in Fig. 6n. Scale bar, 200 μm. Data shown are representative of two independent experiments, each with similar results. P-values were calculated using the log-rank test (**a**) and two-tailed unpaired *t*-tests (**c, e**). Source data are provided as a Source Data file.

Supplementary Table 1. List of EPRS1-interacting candidates identified from interactome analysis

Gene names	Protein names	Protein IDs	Peptides (-LPS, Veh)	Peptides (+LPS, Veh)	Peptides (-LPS, EPRS1)	Peptides (+LPS, EPRS1)	Unique peptides (-LPS, Veh)	Unique peptides (+LPS, Veh)	Unique peptides (-LPS, EPRS1)	Unique peptides (+LPS, EPRS1)	Sequence coverage [%]	Unique sequence coverage [%]	Mol. weight [kDa]	Score	MS/MS count	Intensity (-LPS, Veh)	Intensity (+LPS, Veh)	Intensity (-LPS, EPRS1)	Intensity (+LPS, EPRS1)
A2ML1	Alpha-2-macroglobulin-like protein 1	A8K2U0; H0YGG5	8	5	6	3	8	5	6	3	9.2	9.2	161.1	44.022	21	46659000	6429500	20655000	4381000
ABCF1	ATP-binding cassette sub-family F member 1	Q8NE71; H0YGW7	1	0	4	3	1	0	4	3	4.5	4.5	95.925	13.17	6	185870	0	9994600	4452400
ABHD14B	Alpha/beta hydrolase domain-containing protein 14B	Q96IU4; B4DQI4; F8W9U3	3	4	4	5	3	4	4	5	39.5	39.5	22.345	16.738	16	5599000	3695500	23346000	25110000
ACACA	Acetyl-CoA carboxylase 1;Biotin carboxylase	Q13085	48	43	60	38	39	34	48	29	34.6	28.3	265.55	323.31	213	305000000	182330000	460230000	88633000
ACACB	Acetyl-CoA carboxylase 2;Biotin carboxylase	O00763; F8W8T8; H0YGH5	11	14	22	15	2	5	10	6	11.7	5.7	276.54	16.999	14	3448700	4061600	19704000	3193800
ACADSB	Short/branched chain specific acyl-CoA dehydrogenase, mitochondrial	P45954	2	0	3	2	2	0	3	2	8.3	8.3	47.485	6.0717	3	1573300	0	6637300	2338500
ACADVL	Very long-chain specific acyl-CoA dehydrogenase, mitochondrial	P49748; G3V1M7	1	0	3	0	1	0	3	0	6.6	6.6	70.389	5.7079	3	417980	0	8722300	0
ACAT1	Acetyl-CoA acetyltransferase, mitochondrial	P24752; A0A5F9ZHL1;A0A5F9ZHJ7;A0A5F9ZHJ0;A0A5F9ZHD4;A0A5F9ZI66	4	3	14	15	4	3	14	15	43.6	43.6	45.199	323.31	46	5666900	1584400	182340000	113630000
ACOT7	Cytosolic acyl coenzyme A thioester hydrolase	O00154; K7EKP8	3	0	3	2	3	0	3	2	10.3	10.3	41.796	8.6546	4	3481400	0	5152700	2075400
ACP1	Low molecular weight phosphotyrosine protein phosphatase	P24666; G5E9R5; F2Z2Q9	0	4	2	4	0	4	2	4	33.5	33.5	18.042	82.634	6	0	5014500	1692600	17024000
ACTL6A	Actin-like protein 6A	O96019	1	1	2	2	1	1	2	2	9.3	9.3	47.46	6.9625	6	1240400	499320	10635000	4518600
ACTN1	Alpha-actinin-1	P12814; H9KV75	26	12	21	4	9	2	8	1	33.9	13.7	103.06	27.565	19	27543000	1014000	25671000	229670
ACTN4	Alpha-actinin-4	O43707; F5GXS2	47	32	36	8	30	22	23	5	60.9	41.2	104.85	323.31	143	668910000	82924000	338800000	9231900
ACTR2	Actin-related protein 2	P61160; F5H6T1	5	1	5	2	5	1	5	2	15.7	15.7	44.76	20.383	14	15878000	0	17007000	1810600
ACTR3	Actin-related protein 3	P61158; B4DXW1	8	1	4	0	8	1	4	0	30.9	30.9	47.371	33.715	12	30883000	1571400	21418000	0
AGK	Acylglycerol kinase, mitochondrial	Q53H12; A0A3B3ISY9;A0A	1	1	1	0	1	1	1	0	6.4	6.4	47.137	3.0474	1	844880	2562600	3793100	0

		3B3ISZ0; E9PC15; A0A3B3ITD0;A0A3B3IUC9;A0A3B3ITV0;A0A3B3ITX7;A0A0G2JLG5;E9PG39																	
AGPS	Alkyldihydroxyacetone phosphate synthase, peroxisomal	O00116; A0A1B0GWA2;A0A2R8YEL0	1	0	2	3	1	0	2	3	6.1	6.1	72.911	10.729	4	106560	0	4273300	3409900
AHNAK	Neuroblast differentiation-associated protein AHNAK	Q09666	6	2	19	3	6	2	19	3	9.8	9.8	629.09	58.441	25	14030000	3224800	110810000	1728600
AHSA1	Activator of 90 kDa heat shock protein ATPase homolog 1	O95433; G3V438; H0YJG7	4	3	8	6	4	3	8	6	37	37	38.274	29.729	16	16774000	5426700	122540000	60395000
AHSG	Alpha-2-HS-glycoprotein;Alpha-2-HS-glycoprotein chain A;Alpha-2-HS-glycoprotein chain B	P02765; C9JV77	2	2	0	0	2	2	0	0	4.6	4.6	39.34	6.4448	3	2660100	7408100	0	0
AIFM1	Apoptosis-inducing factor 1, mitochondrial	O95831; A0A6Q8PG15;A0A6Q8PFM5;A0A6Q8PFE1; A0A6Q8PFA7;A0A6Q8PFQ8;A0A6Q8PFS4; A0A6Q8PHC0;A0A6Q8PHJ9;A0A6Q8PFW2	3	3	10	4	3	3	10	4	22.7	22.7	66.9	59.658	20	5284600	4895500	37415000	8752200
AIMP1	Aminoacyl tRNA synthase complex-interacting multifunctional protein 1;Endothelial monocyte-activating polypeptide 2	Q12904; A0A5F9ZHC5	3	2	15	14	3	2	15	14	66.3	66.3	34.352	288.17	44	2035000	2041100	273010000	194500000
AIMP2	Aminoacyl tRNA synthase complex-interacting multifunctional protein 2	Q13155; A8MU58	1	0	7	5	1	0	7	5	31.2	31.2	35.348	88.668	22	1778600	0	99207000	44582000
AK2	Adenylate kinase 2, mitochondrial;Adenylate kinase 2, mitochondrial, N-terminally processed;Adenylate kinase 2, mitochondrial	P54819; F8VY04; A0A5K1VW67;F8W1A4	0	0	2	0	0	0	2	0	17.2	17.2	26.477	12.389	2	0	0	3590400	0

AKT1	RAC-alpha serine/threonine-protein kinase	P31749	0	0	1	2	0	0	1	2	4.4	4.4	55.686	4.9331	4	0	0	776620	6205000
ALDH18A1	Delta-1-pyrroline-5-carboxylate synthase;Glutamate 5-kinase;Gamma-glutamyl phosphate reductase	P54886	2	2	9	7	2	2	9	7	13.8	13.8	87.301	58.468	17	2147300	3075000	37555000	30646000
ALDH1A3	Aldehyde dehydrogenase family 1 member A3	P47895; H0Y2X5	0	0	8	0	0	0	7	0	18.4	16.2	56.108	44.04	9	0	0	23608000	0
ALDH2	Aldehyde dehydrogenase, mitochondrial	P05091	0	0	3	0	0	0	3	0	7	7	56.381	6.3672	3	0	0	7535300	0
ALDH9A1	4-trimethylaminobutyraldehyde dehydrogenase	P49189	1	0	2	1	1	0	2	1	3.4	3.4	53.801	3.1757	2	252840	0	8791100	267700
ALDOA	Fructose-bisphosphate aldolase A;Fructose-bisphosphate aldolase	P04075; J3KPS3; H3BQN4;H3BUH7;H3BR04;H3BPS8;H3BMQ8	12	3	14	9	12	3	14	9	47	47	39.42	146.35	60	35215000	13171000	378570000	16179000
ALOX12B	Arachidonate 12-lipoxygenase, 12R-type	O75342; A0A3B3IRK2	5	4	6	3	5	4	6	3	9.7	9.7	80.355	11.291	24	24710000	11752000	27400000	5391500
ANXA1	Annexin A1;Annexin	P04083; Q5T3N1	5	3	12	4	5	3	12	4	38.4	38.4	38.714	191.41	30	20543000	9036000	185880000	4226600
ANXA2	Annexin A2;Annexin;Putative annexin A2-like protein	P07355; H0YN42; H0YMU9;H0YMD0;H0YKS4;A6NMY6;H0YM50;H0YNP5;H0YMM1	16	14	19	15	16	14	19	15	61.7	61.7	38.604	323.31	194	295800000	149350000	688300000	140590000
ANXA8L2	Annexin A8-like protein 2;Annexin A8;Annexin	Q5VT79; P13928; B4DTF2; A0A075B752;A0A087WUP0;A0A087WTN9	0	0	4	0	0	0	4	0	20.5	20.5	36.879	11.817	4	0	0	12924000	0
API5	Apoptosis inhibitor 5	Q9BZZ5; G3V1C3	0	0	2	1	0	0	2	1	5.9	5.9	59.004	9.2592	2	0	0	4995100	1321600
APOA1	Apolipoprotein A-I;Proapolipoprotein A-I;Truncated apolipoprotein A-I	P02647; F8W696	7	12	7	1	6	11	7	1	55.8	49.8	30.777	64.46	23	13259000	67219000	28370000	509770
APOA2	Apolipoprotein A-II;Proapolipoprotein A-II;Truncated apolipoprotein A-II	P02652; V9GYG9;V9GYM3;V9GYE3;V9GYC1;V9GYS1	2	3	2	1	2	3	2	1	42	42	11.175	67.704	9	5241900	12545000	27429000	234450
APOA4	Apolipoprotein A-IV	P06727	1	4	1	0	1	4	1	0	13.6	13.6	45.371	8.0605	5	332000	6708500	775440	0

APOD	Apolipoprotein D	P05090; C9JF17; C9JX71	1	1	1	0	1	1	1	0	9.5	9.5	21.275	2.0298	3	5112100	1303400	1436500	0
APRT	Adenine phosphoribosyltransferase	P07741; H3BQF1; H3BSW3;H3BQZ9;H3BQB1	0	1	2	1	0	1	2	1	17.2	17.2	19.608	7.2905	2	0	372310	2899900	564500
ARF3;ARF1;ARF5	ADP-ribosylation factor 3;ADP-ribosylation factor 1;ADP-ribosylation factor 5	P61204; P84077; F5H423; P84085; F5H0C7; C9J1Z8	0	4	6	5	0	3	3	3	34.3	17.1	20.601	6.9623	10	0	5753500	39568000	18506000
ARF4	ADP-ribosylation factor 4	P18085; C9JPM4; C9JAK5	0	6	8	7	0	5	5	5	44.4	27.2	20.511	31.797	18	0	25216000	84565000	85752000
ARG1	Arginase-1	P05089; A0A5F9ZH78;A0A5F9ZGN6	13	14	13	14	13	14	13	14	61.8	61.8	34.735	84.888	175	307490000	164630000	319390000	162110000
ARHGAP1	Rho GTPase-activating protein 1	Q07960; H0YE29	0	0	5	3	0	0	5	3	20.7	20.7	50.435	19.189	9	0	0	28426000	8230100
ARHGAP15	Rho GTPase-activating protein 15	Q53QZ3	0	1	2	3	0	1	2	3	5.7	5.7	54.544	4.1901	4	0	2419500	10218000	4780300
ARHGDIB	Rho GDP-dissociation inhibitor 2	P52566; F5H2R5; F5H6Q0; F5H3P3; H0YGX7	0	0	2	0	0	0	2	0	14.9	14.9	22.988	12.833	2	0	0	6486500	0
ARHGEF1	Rho guanine nucleotide exchange factor 1	Q92888; M0QZR4;M0QYC1	0	1	8	10	0	1	8	10	14.7	14.7	102.43	45.306	15	0	354760	22121000	14050000
ARHGEF2	Rho guanine nucleotide exchange factor 2	Q92974; V9GYM8;A0A5F9ZI21	0	0	4	4	0	0	4	4	6.3	6.3	111.54	43.684	7	0	0	10812000	5130900
ARL1	ADP-ribosylation factor-like protein 1	P40616; B4DZG7; F8VYN9	0	0	2	1	0	0	2	1	14.4	14.4	20.417	4.122	2	0	0	10203000	7780800
ARPC1B	Actin-related protein 2/3 complex subunit 1B	O15143; C9JEY1; C9K057; C9JBJ7; F8VXW2;A0A1W2PNV4	5	1	3	0	5	1	3	0	20.2	20.2	40.949	16.194	6	14647000	377880	2092300	0
ARPC2	Actin-related protein 2/3 complex subunit 2	O15144	6	2	6	1	6	2	6	1	24.7	24.7	34.333	36.07	15	24661000	1581300	17349000	189520
ARPC3	Actin-related protein 2/3 complex subunit 3	O15145; C9JZD1	3	1	2	1	3	1	2	1	20.8	20.8	20.546	6.4399	5	7457800	194930	3536400	149950
ARPC4	Actin-related protein 2/3 complex subunit 4	P59998; F8WDD7;F8WCF6;A0A0A6YYG9;H7C0A3	0	3	3	1	0	3	3	1	17.9	17.9	19.667	6.868	9	0	3310200	18767000	699680
ASPRV1	Retroviral-like aspartic protease 1	Q53RT3	1	1	2	1	1	1	2	1	13.1	13.1	36.991	4.4626	5	2406300	2128600	30844000	6228300
ASS1	Argininosuccinate synthase	P00966	2	2	6	4	2	2	6	4	16	16	46.53	11.591	19	7599000	1110500	67177000	16433000

ATAD3A	ATPase family AAA domain-containing protein 3A	Q9NVI7	11	10	12	10	3	1	3	2	26.5	8.5	71.368	56.467	47	40368000	34086000	104920000	36528000
ATL3	Atlastin-3	Q6DD88; F5GWF8;F5H6I7	2	0	2	2	2	0	2	2	4.1	4.1	60.541	4.9756	6	1163500	0	9189800	3039100
ATP1A1	Sodium/potassium-transporting ATPase subunit alpha-1	P05023	0	1	9	8	0	1	9	8	13.8	13.8	112.89	21.822	14	0	1059700	29227000	17179000
ATP1B3	Sodium/potassium-transporting ATPase subunit beta-3	P54709; C9JA36; H7C4L9; C9JXZ1	0	0	2	1	0	0	2	1	7.9	7.9	31.512	2.1284	2	0	0	4995600	385190
ATP5A1	ATP synthase subunit alpha, mitochondrial	P25705; K7EK77	19	17	23	22	19	17	23	22	48.6	48.6	59.75	323.31	220	138200000	139060000	1711800000	653960000
ATP5B	ATP synthase subunit beta, mitochondrial	P06576; H0YH81; F8W079	15	13	21	21	15	13	21	21	67.7	67.7	56.559	323.31	165	138370000	112240000	968420000	585780000
ATP5C1	ATP synthase subunit gamma, mitochondrial	P36542	6	6	8	7	6	6	8	7	35.6	35.6	32.996	78.961	42	20682000	12441000	176800000	96840000
ATP5D	ATP synthase subunit delta, mitochondrial	P30049	0	0	2	0	0	0	2	0	13.7	13.7	17.49	2.7803	2	0	0	4946600	0
ATP5F1	ATP synthase F(0) complex subunit B1, mitochondrial	P24539	1	1	3	3	1	1	3	3	19.5	19.5	28.908	12.324	7	801160	1772200	18727000	7894000
ATP5O	ATP synthase subunit O, mitochondrial	P48047; A0A494C0K9;H7C0C1;H7C086	1	2	4	3	1	2	4	3	21.6	21.6	23.277	6.8506	7	752690	837150	10909000	9279400
ATP6V1A	V-type proton ATPase catalytic subunit A	P38606; C9JA17; C9JVW8	1	1	4	1	1	1	4	1	8.8	8.8	68.303	5.4549	5	790930	451050	23703000	942780
ATP6V1H	V-type proton ATPase subunit H	Q9UI12; G3V126	1	0	4	3	1	0	4	3	13.9	13.9	55.882	30.491	6	361340	0	14631000	5902300
ATXN2L	Ataxin-2-like protein	Q8WWM7;H3BRB0;A0A0C4DGL7	2	2	0	0	2	2	0	0	2.1	2.1	113.37	6.6285	4	6703400	15245000	0	0
AZGP1	Zinc-alpha-2-glycoprotein	P25311; C9JEV0	10	10	10	9	10	10	10	9	45	45	34.258	70.572	136	222410000	163410000	252850000	105900000
AZU1	Azurocidin	P20160	4	4	4	4	4	4	4	4	16.3	16.3	26.885	47.174	36	103830000	49776000	116170000	34116000
BLMH	Bleomycin hydrolase	Q13867; K7ES02; K7ESE8; J3KSD8	6	11	7	7	6	11	7	7	30.3	30.3	52.562	56.607	50	39757000	69706000	60465000	21318000
BLVRB	Flavin reductase (NADPH)	P30043; A0A2R8Y7Y9;M0QZL1;M0R192;A0A2R8YEP4	1	0	1	1	1	0	1	1	11.7	11.7	22.119	6.5703	4	3825800	0	4877300	568610
BSG	Basigin	P35613; A0A087WUV8;A0A087X2B5;R4GMX5;I3L192	0	0	2	2	0	0	2	2	4.9	4.9	42.2	3.6267	4	0	0	9590600	3156300

BTK	Tyrosine-protein kinase BTK;Non-specific protein-tyrosine kinase	Q5JY90; Q06187; U3NG26	1	0	5	2	1	0	5	2	11.2	11.2	55.88	10.442	10	2424900	0	14234000	3012900
BUB3	Mitotic checkpoint protein BUB3	O43684; J3QT28	0	0	3	1	0	0	3	1	11.3	11.3	37.154	40.992	3	0	0	1712700	777930
BUD31	Protein BUD31 homolog	P41223; C9JCD9	1	1	1	2	1	1	1	2	13.9	13.9	17	2.9587	2	2264500	1674900	2452800	5911100
BYSL	Bystin	Q13895; F8WBL2	1	0	2	2	1	0	2	2	6.4	6.4	49.601	3.2453	5	359850	0	2953400	1681100
C10orf11	Leucine-rich repeat-containing protein C10orf11	Q9H2I8; A0A087WWI0;M0R2H0;A0A087WZZ7	1	1	2	2	1	1	2	2	16.7	16.7	22.567	4.6715	4	1020400	176620	3927700	2624800
C1QBP	Complement component 1 Q subcomponent-binding protein, mitochondrial	Q07021;I3L3Q7;I3L3B0	0	0	2	2	0	0	2	2	9.6	9.6	31.362	4.4024	4	0	0	5909800	8149700
C2orf47	Uncharacterized protein C2orf47, mitochondrial	Q8WWC4	0	1	2	2	0	1	2	2	7.6	7.6	32.544	2.5548	3	0	858690	6962700	4147900
C3	Complement C3;Complement C3 beta chain;C3-beta-c;Complement C3 alpha chain;C3a anaphylatoxin;Acylation stimulating protein;Complement C3b alpha chain;Complement C3c alpha chain fragment 1;Complement C3dg fragment;Complement C3g fragment;Complement C3d fragment;Complement C3f fragment;Complement C3c alpha chain fragment 2	P01024	1	5	14	1	1	5	14	1	12	12	187.15	243.92	17	697640	6153400	78122000	180320
C4B;C4A	Complement C4-B;Complement C4 beta chain;Complement C4-B alpha chain;C4a anaphylatoxin;C4b-B;C4d-B;Complement C4 gamma chain;Complement C4-A;Complement C4 beta chain;Complement C4-A alpha chain;C4a anaphylatoxin;C4b-A;C4d-A;Complement C4 gamma chain	P0C0L5; P0C0L4; A0A3B3ISA6;F5GXS0;A0A140TA49;A0A140TA44;A0A140TA32;A0A140TA29;A0A0G2JL54;A0A0G2JPR0	1	0	3	0	1	0	3	0	2.5	2.5	192.75	12.653	3	177610	0	7741900	0
CA1	Carbonic anhydrase 1	P00915; E5RII2;E5RGU8;	2	0	0	0	2	0	0	0	14.2	14.2	28.87	41.22	7	22443000	0	0	0

		E5RFL2; E5RIF9; E5RG43; E5RH81; E5RHP7																	
CAD	CAD protein;Glutamine-dependent carbamoyl-phosphate synthase;Aspartate carbamoyltransferase; Dihydroorotase	P27708; F8VPD4	2	2	8	3	2	2	8	3	4.9	4.9	242.98	19.59	8	1097000	877780	17748000	2740200
CALM2; CALM3; CALM1		P0DP25; P0DP24; P0DP23; F8WBR5 ;M0QZ52 ;G3V479 ;E7EMB3	2	1	3	1	2	1	3	1	30.9	30.9	16.837	146.58	12	10676000	2067000	60167000	3675900
CALML3	Calmodulin-like protein 3	P27482	6	2	5	2	6	2	5	2	57.7	57.7	16.891	211.11	22	49162000	2345000	91712000	3057400
CALML5	Calmodulin-like protein 5	Q9NZT1	10	8	11	10	10	8	11	10	80.8	80.8	15.892	323.31	111	564560000	194770000	434330000	219460000
CALR	Calreticulin	P27797; K7EJB9	0	1	7	0	0	1	7	0	24.7	24.7	48.141	49.433	10	0	178620	41836000	0
CAND1	Cullin-associated NEDD8-dissociated protein 1	Q86VP6; A0A0C4 DGH5	2	2	11	11	2	2	11	11	16	16	136.37	40.988	28	3397000	2677300	62879000	34127000
CANX	Calnexin	P27824	0	0	7	4	0	0	7	4	14.4	14.4	67.567	16.633	12	0	0	69946000	10814000
CAP1	Adenylyl cyclase-associated protein 1;Adenylyl cyclase-associated protein	Q01518; Q5T0R7; Q5T0R6; Q5T0R5; Q5T0R4; Q5T0R3; Q5T0R2; Q5T0R1	0	0	4	0	0	0	4	0	14.3	14.3	51.901	45.65	2	0	0	6591200	0
CAPG	Macrophage-capping protein	P40121; H7C0X8; E7ENU9	2	0	2	1	2	0	2	1	7.2	7.2	38.498	11.421	4	1714000	0	9222500	1181700
CAPZA1	F-actin-capping protein subunit alpha-1	P52907	12	6	12	9	10	6	10	8	66.1	56.6	32.922	215.55	44	166170000	16031000	98696000	19965000
CAPZA2	F-actin-capping protein subunit alpha-2	P47755	4	0	3	1	2	0	1	0	20.3	10.8	32.949	38.855	2	2497700	0	837060	0
CAPZB	F-actin-capping protein subunit beta	P47756; B1AK87; B1AK85; B1AK88	7	3	5	2	7	3	5	2	26.7	26.7	31.35	32.513	21	40302000	5618400	18226000	3095600
CASP14	Caspase-14;Caspase-14 subunit p17, mature form;Caspase-14 subunit p10, mature form;Caspase-14 subunit p20, intermediate form;Caspase-14 subunit p8, intermediate form	P31944	16	13	15	16	16	13	15	16	50.4	50.4	27.679	157.49	225	273050000	185930000	278750000	137870000
CAT	Catalase	P04040	17	16	15	13	17	16	15	13	35.5	35.5	59.755	146.64	155	181800000	102340000	195860000	79967000

CBR1	Carbonyl reductase [NADPH] 1;Carbonyl reductase [NADPH] 3	P16152;E9PQ63;A8MTM1;O75828	0	0	3	1	0	0	3	1	15.5	15.5	30.375	19.113	4	0	0	9099900	481480
CBX3	Chromobox protein homolog 3	Q13185;B8ZZ43;C9JMM0;S4R2Y4	0	0	2	0	0	0	2	0	14.2	14.2	20.811	36.456	2	0	0	7166800	0
CCDC88A	Girdin	Q3V6T2;A0A087WXD9;A0A2R8YG52;A0A2R8YGU1;A0A2R8Y4X4;A0A2R8Y4W8;A0A2R8YG73;A0A2R8Y7B1;A0A2U3TZV9;A0A2R8YD81;A0A2R8Y7D9;A0A2R8Y6B2;A0A2R8Y820	1	1	2	1	1	1	2	1	1.3	1.3	216.04	4.5986	6	5744200	8479400	7376200	250700
CCT2	T-complex protein 1 subunit beta	P78371;F8VQ14;F5GWF6	3	3	10	7	3	3	10	7	26.5	26.5	57.488	113.68	20	6835500	3493500	66794000	15110000
CCT3	T-complex protein 1 subunit gamma	P49368;B4DUR8	12	12	17	13	12	12	17	13	39.3	39.3	60.533	138.47	57	63199000	59413000	204850000	85472000
CCT4	T-complex protein 1 subunit delta	P50991	11	10	14	12	11	10	14	12	34.1	34.1	57.924	183.12	64	64606000	60614000	251700000	47881000
CCT5	T-complex protein 1 subunit epsilon	P48643;E7ENZ3;B7ZAR1;E9PCA1	2	3	6	3	2	3	6	3	14.2	14.2	59.67	15.037	16	6788600	4101300	39013000	6884300
CCT6A	T-complex protein 1 subunit zeta	P40227	7	10	16	14	7	10	16	14	37.5	37.5	58.024	114.16	46	30626000	22742000	171790000	66875000
CCT7	T-complex protein 1 subunit eta	Q99832	2	3	8	3	2	3	8	3	21.2	21.2	59.366	23.37	14	2866800	6043500	37650000	7103700
CCT8	T-complex protein 1 subunit theta	P50990;H7C4C8	1	1	9	1	1	1	9	1	19.2	19.2	59.62	19.576	11	1767900	926740	36464000	1613900
CDK1	Cyclin-dependent kinase 1	P06493;A0A024QZP7;A0A087WZZ9;E5RIU6	1	3	6	6	1	2	5	5	31.3	28.6	34.095	19.074	17	5379000	3439900	25136000	21103000
CDK11B	Cyclin-dependent kinase 11B;Cyclin-dependent kinase 11A	P21127;Q9UQ88;A0A0D9SEI3;Q5QPR3;A0A0D9SER5;A0A0D9SEN2;Q5QPQ9;E9PFJ2;A0A087	1	1	1	2	1	1	1	2	2.4	2.4	92.619	2.0128	2	8719100	2130700	1533100	3550800

		WYI9;A0A087X1I0;J3KS35;E7ESP2;Q5QPR4																
CDK6	Cyclin-dependent kinase 6	Q00534	0	2	6	5	0	1	5	4	20.9	18.4	36.938	11.531	6	0	409750	17311000	4563500
CDKN2AIP	CDKN2A-interacting protein	Q9NXV6; J3KNE1; D6RGD2	0	1	2	1	0	1	2	1	4.1	4.1	61.124	27.63	3	0	180980	3089400	867250
CDSN	Corneodesmosin	Q15517; Q2L6G8; G8JLG2	3	3	3	3	3	3	3	3	6	6	51.522	14.682	57	54932000	99187000	88774000	44175000
CFL1	Cofilin-1	P23528; G3V1A4; E9PP50; E9PK25; E9PQB7; E9PLJ3; E9PS23	3	8	11	10	3	8	11	10	65.1	65.1	18.502	271.46	38	10656000	54375000	316760000	244410000
CHCHD3	MICOS complex subunit MIC19	Q9NX63; C9JRZ6; F8WAR4;A0A286YEX5	2	1	3	2	2	1	3	2	20.7	20.7	26.152	13.163	6	2467600	521410	6810700	2970200
CKAP4	Cytoskeleton-associated protein 4	Q07065	0	0	7	1	0	0	7	1	17.4	17.4	66.022	36.269	7	0	0	22328000	718300
CKM	Creatine kinase M-type;Creatine kinase M-type, N-terminally processed	P06732	2	0	0	0	2	0	0	0	8.7	8.7	43.101	17.72	2	6160300	0	0	0
CLIC1	Chloride intracellular channel protein 1	O00299	0	0	4	0	0	0	4	0	20.7	20.7	26.922	15.967	6	0	0	34716000	0
CLPP	ATP-dependent Clp protease proteolytic subunit, mitochondrial;ATP-dependent Clp protease proteolytic subunit	Q16740; M0R208	0	0	2	1	0	0	2	1	10.8	10.8	30.18	2.692	1	0	0	1962100	834580
CLTA	Clathrin light chain A	P09496; F8WF69	1	0	2	3	1	0	2	3	10.5	10.5	27.076	6.5775	7	2537200	0	8272200	7052900
CLTC	Clathrin heavy chain 1;Clathrin heavy chain	Q00610; A0A087WVQ6	5	8	22	17	5	8	22	17	19.6	19.6	191.61	76.233	53	8576800	10745000	90913000	27321000
CLU	Clusterin;Clusterin beta chain;Clusterin alpha chain;Clusterin	P10909; H0YC35; H0YAS8; E7ERK6; H0YLK8	1	2	1	0	1	2	1	0	5.1	5.1	52.494	4.2629	6	2490900	6872400	5854100	0
CMPK1	UMP-CMP kinase	P30085; A0A494BXC7	0	0	3	1	0	0	3	1	13.8	13.8	22.222	20.87	4	0	0	4539600	1096100
COL1A1	Collagen alpha-1(I) chain	P02452	3	2	3	5	3	2	3	5	5.3	5.3	138.94	17.223	14	5586800	2796500	6819400	54317000
COPA	Coatomer subunit alpha;Xenin;Proxenin	P53621; A0A3B3IT15;A0A3B3IU89; A0A3B3ISC6;A0A3B3IS84	2	0	3	1	2	0	3	1	2.9	2.9	138.34	4.4979	4	887960	0	7800800	1172100

COPB1	Coatomer subunit beta	P53618; E9PP73	1	1	9	10	1	1	9	10	14.7	14.7	107.14	37.311	17	609350	664930	29390000	25890000
COPE	Coatomer subunit epsilon	O14579; M0R061; M0QXB4	1	0	3	3	1	0	3	3	14	14	34.482	10.929	4	1325500	0	9772300	4855700
COPG1	Coatomer subunit gamma-1	Q9Y678	1	0	9	9	1	0	9	9	15.4	15.4	97.717	43.632	21	2808300	0	35924000	21335000
CORO1A	Coronin-1A;Coronin	P31146; H3BRY3	1	1	2	1	1	1	2	1	5.9	5.9	51.026	8.7639	5	1502700	834380	5213800	1228400
CORO1C	Coronin-1C;Coronin	Q9ULV4; B4E3S0; F8VV53; F8W1H8; F8VUX3; F8VSA4; F8VRE9; F8VTT6; F8VVB7	0	0	2	0	0	0	2	0	6.5	6.5	53.248	2.9228	1	0	0	6003900	0
CPA4	Carboxypeptidase A4	Q9UI42; B7Z5J4	3	2	3	3	3	2	3	3	14.7	14.7	47.351	13.722	11	4356600	3155000	8716500	2661700
CPNE1	Copine-1	Q99829; F2Z2V0; B0QZ18; A6PVH9	0	1	3	2	0	1	3	2	7.4	7.4	59.058	66.181	8	0	1092400	10802000	2613800
CRABP2	Cellular retinoic acid-binding protein 2	P29373; Q5SYZ4	0	0	4	0	0	0	4	0	32.6	32.6	15.693	7.5324	4	0	0	22059000	0
CRYZ	Quinone oxidoreductase	Q08257; C9JH92; A6NP24	0	1	2	0	0	1	2	0	8.8	8.8	35.206	3.0894	2	0	111310	2668800	0
CS	Citrate synthase, mitochondrial;Citrate synthase	O75390; H0YIC4; A0A0C4DGI3;B4DJV2	0	0	2	0	0	0	2	0	4.7	4.7	51.712	2.2268	2	0	0	4445200	0
CSE1L	Exportin-2	P55060	0	0	9	6	0	0	9	6	13.9	13.9	110.42	48.533	16	0	0	26390000	10742000
CSK	Tyrosine-protein kinase CSK	P41240	1	0	7	4	1	0	7	4	20.7	20.7	50.704	15.032	11	634070	0	33740000	15305000
CST6	Cystatin-M	Q15828	1	2	3	1	1	2	3	1	28.2	28.2	16.511	6.7241	22	7646600	12165000	45013000	7100800
CSTA	Cystatin-A;Cystatin-A, N-terminally processed	P01040; C9J0E4	7	9	9	9	7	9	9	9	76.5	76.5	11.006	182.05	135	283250000	359390000	551980000	250540000
CSTB	Cystatin-B	P04080	0	0	3	1	0	0	3	1	45.9	45.9	11.139	13.216	6	0	0	48678000	1403100
CTPS1	CTP synthase 1;CTP synthase	P17812; A0A3B3IRI2;A0A3B3IRQ8;A0A3B3ITF6;A0A3B3ITB8; B4E1E0	2	0	11	4	2	0	11	4	24.4	24.4	66.69	140.54	22	1955600	0	59051000	14604000
CTSD	Cathepsin D;Cathepsin D light chain;Cathepsin D heavy chain	P07339; A0A1B0GW44;A0A1B0GV23;A0A1B0GVD5;A0A1B0GWE8;	4	4	5	3	4	4	5	3	17.2	17.2	44.552	23.436	17	40247000	9297800	30120000	4134800

		H7C469;A0A1B0GVP3;H7C1V0;A0A1B0GU92;A0A1B0GU03																	
CTSG	Cathepsin G	P08311	9	8	9	9	9	8	9	9	43.9	43.9	28.837	254.86	124	460230000	238690000	807320000	499400000
CUL4A	Cullin-4A	Q13619;A0A0A0MR50	0	0	3	1	0	0	3	1	4.6	4.6	87.679	8.8752	4	0	0	4130100	2285500
CYB5A	Cytochrome b5	P00167	2	0	0	0	2	0	0	0	25.4	25.4	15.33	12.784	2	5064800	0	0	0
DARS	Aspartate--tRNA ligase, cytoplasmic	P14868	17	15	25	24	17	15	25	24	59.3	59.3	57.136	306.98	158	57849000	41194000	811590000	575390000
DCD	Dermcidin;Survival-promoting peptide;DCD-1	P81605	6	6	5	6	6	6	5	6	47.3	47.3	11.284	59.571	109	500190000	359120000	304190000	204410000
DCTN1	Dynactin subunit 1	Q14203;Q6AWB1;E7EX90	0	1	1	1	0	1	1	1	1.8	1.8	141.69	11.083	2	0	267520	1360400	1411200
DDOST	Dolichyl-diphosphooligosaccharide--protein glycosyltransferase 48 kDa subunit	P39656;A0A0C4DGS1;U3KQ84	3	0	6	2	3	0	6	2	19.5	19.5	50.8	66.084	6	1022600	0	19131000	651530
DDX1	ATP-dependent RNA helicase DDX1	Q92499;A0A087X2G1;F1T0B3	0	0	2	0	0	0	2	0	4.3	4.3	82.431	3.551	1	0	0	4260000	0
DDX17	Probable ATP-dependent RNA helicase DDX17	Q92841;A0A1X7SBZ2;A0A5H1ZRQ2	2	3	9	6	1	2	5	3	14.1	9.6	80.272	72.04	11	519990	2267300	17208000	8093800
DDX18	ATP-dependent RNA helicase DDX18	Q9NVP1;H7C452	0	0	6	3	0	0	6	3	11.2	11.2	75.406	12.732	10	0	0	18301000	3432200
DDX19A;DDX19B	ATP-dependent RNA helicase DDX19A;ATP-dependent RNA helicase DDX19B	Q9NUU7;Q9UMR2;I3L0H8;F6QDS0;H3BQK0;H3BTB3;H3BMQ5;H3BN59;I3L352	1	0	4	1	1	0	4	1	9.6	9.6	53.974	15.97	5	347170	0	11604000	1625000
DDX39A;DDX39B	ATP-dependent RNA helicase DDX39A;Spliceosome RNA helicase DDX39B	O00148;K7EQN7;Q13838	7	6	11	9	6	5	8	6	31.9	24.4	49.129	111.58	54	25379000	13579000	216120000	93018000
DDX3X;DDX3Y	ATP-dependent RNA helicase DDX3X;ATP-dependent RNA helicase DDX3Y	O00571;A0A2R8YF78;A0A2R8Y5G6;A0A0D9SF53;A0A2R8Y645;A0A2R8Y4A4;A0A2R8YFS5;	4	3	12	11	4	3	12	11	27.9	27.9	73.243	61.845	37	10939000	3983900	78397000	29478000

		A0A2U3TZJ9;A0A2R8YFR4;A0A2R8YCW1;A0A0D9SG12;A0A0D9SFB3;A0A2R8YDT5;A0A2R8Y7T2;F6S8Q4;O15523;A0A2R8YDH3;A0A0J9YVQ7																	
DDX47	Probable ATP-dependent RNA helicase DDX47	Q9H0S4; F5H1N9	0	1	3	1	0	1	3	1	6.8	6.8	50.646	6.2976	3	0	276970	6690100	460310
DDX5	Probable ATP-dependent RNA helicase DDX5	P17844; J3KTA4; J3KRZ1	3	1	9	7	2	0	5	4	18.7	13.4	69.147	23.307	16	3872300	5780100	49103000	22855000
DEFA3;DEFA1	Neutrophil defensin 3;HP 3-56;Neutrophil defensin 2;Neutrophil defensin 1;HP 1-56;Neutrophil defensin 2	P59666; P59665	3	2	1	3	3	2	1	3	20.2	20.2	10.245	6.1743	11	17140000	18350000	22013000	26342000
DHX9	ATP-dependent RNA helicase A	Q08211	7	3	12	9	7	3	12	9	10.9	10.9	140.96	52.226	18	14444000	1733000	50882000	9360000
DIS3	Exosome complex exonuclease RRP44	Q9Y2L1; G3V1J5; F2Z2C0	1	0	6	4	1	0	6	4	8.8	8.8	109	9.3056	5	731690	0	11295000	3178000
DLAT	Dihydrolipoyllysine-residue acetyltransferase component of pyruvate dehydrogenase complex, mitochondrial;Acetyltransferase component of pyruvate dehydrogenase complex	P10515; H0YDD4; E9PEJ4	2	2	2	1	2	2	2	1	3.9	3.9	68.996	3.0513	3	3972000	3348400	5717200	1775500
DNAJA1	DnaJ homolog subfamily A member 1	P31689	1	0	4	3	1	0	4	3	18.1	18.1	44.868	32.433	10	912140	0	28796000	6553800
DNAJA2	DnaJ homolog subfamily A member 2	O60884	0	0	2	3	0	0	2	3	12.1	12.1	45.745	13.665	6	0	0	17029000	8845700
DNAJB6; DNAJB2; DNAJB3; DNAJB8	DnaJ homolog subfamily B member 6;DnaJ homolog subfamily B member 2;DnaJ homolog subfamily B member 3;DnaJ homolog subfamily B member 8	O75190; C9J2C4; A0A0J9YX62;P25686;Q8WWF6;Q8NHS0;C9JB42;C9JDR7;C9JDX6;F8WCZ4;C9J2P2;E9PH18;C9JRD2;C9JXB9	2	0	2	1	2	0	2	1	7.1	7.1	36.087	12.509	6	444860	0	9059900	1595400

DNAJC7	DnaJ homolog subfamily C member 7	Q99615; K7EIH8; A0A6I8PUD4;A0A6I8PS32; A0A6I8PIK4;A0A6I8PR89; A0A6I8PRZ0;K7ER44;A0A6I8PLE7; A0A6I8PU89;A0A6I8PUC2;K7EPP7;K7EK03;A0A6I8PU73;K7EJE9;K7ESP1;K7ELJ8;K7EJV7;K7EN19;K7EJL5;A0A6I8PR93	0	1	2	1	0	1	2	1	4.9	4.9	56.44	4.1631	2	0	307910	4403300	1166400
DNM2	Dynamin-2	P50570; K7EMQ3	0	0	5	5	0	0	5	5	7.4	7.4	98.063	16.435	11	0	0	14301000	9214700
DOCK2	Dedicator of cytokinesis protein 2	Q92608; E5RFJ0	1	0	1	0	1	0	1	0	2.4	2.4	211.95	3.8334	2	42898000	0	1199500	0
DPM1	Dolichol-phosphate mannosyltransferase subunit 1	O60762; Q5QPK2;H0Y368; Q5QPJ9	1	1	4	3	1	1	4	3	19.2	19.2	29.634	8.8634	6	687250	385820	10090000	4636400
DSC1	Desmocollin-1	Q08554	15	14	19	17	15	14	19	17	29	29	99.986	236.89	301	701020000	565990000	878860000	313520000
DSC3	Desmocollin-3	Q14574	5	4	7	6	5	4	7	6	11	11	99.968	36.487	56	47935000	29610000	80867000	24964000
DSG1	Desmoglein-1	Q02413	32	30	32	28	32	30	32	28	39.8	39.8	113.75	323.31	516	1144000000	858810000	1261200000	714710000
DSP	Desmoplakin	P15924	118	90	111	97	118	90	111	97	45.6	45.6	331.77	323.31	1306	3438800000	1574800000	3073200000	1272500000
DSTN	Destrin	P60981; F6RFD5	0	1	2	1	0	1	2	1	13.9	13.9	18.506	4.1592	3	0	537090	3019700	1815300
DYNC1H1	Cytoplasmic dynein 1 heavy chain 1	Q14204	0	0	3	0	0	0	3	0	1	1	532.4	6.8967	3	0	0	6116600	0
DYNC1LI1	Cytoplasmic dynein 1 light intermediate chain 1	Q9Y6G9; E9PHI6	1	1	5	6	1	1	5	6	19.9	19.9	56.578	69.657	11	1028700	481120	18363000	10422000
ECH1	Delta(3,5)-Delta(2,4)-dienoyl-CoA isomerase, mitochondrial	Q13011; M0QZW4;M0R248	1	1	3	2	1	1	3	2	11.6	11.6	35.816	8.274	4	959720	267320	6695300	2975700
ECM1	Extracellular matrix protein 1	Q16610	2	2	3	3	2	2	3	3	8.7	8.7	60.673	53.513	13	10742000	11302000	19117000	9366800
EEF1A1	Elongation factor 1-alpha 1	P68104; Q5VTE0; A0A087WVQ9;A0A087W	10	8	13	14	10	8	13	14	57.8	57.8	50.14	251.89	203	308130000	175870000	1694200000	812600000

		V01;Q05639;A0A2U3TZH3																	
EEF1B2	Elongation factor 1-beta	P24534; F8WF65; F2Z2G2; C9JZW3	1	0	2	2	1	0	2	2	12.4	12.4	24.763	78.955	4	959650	0	12483000	1521000
EEF1D	Elongation factor 1-delta	P29692; E9PMW7;E9PK01;E9PI39; E9PQ49; E9PL12; E9PPR1; E9PN91; E9PIZ1; E9PL71; E9PRY8; H0YCK7; A0A087X1X7	0	0	3	1	0	0	3	1	16	16	31.121	16.655	3	0	0	17594000	737910
EEF1E1	Eukaryotic translation elongation factor 1 epsilon-1	O43324; D6RBD7; C9J1V9; H0YAL7	0	1	9	10	0	1	9	10	63.2	63.2	19.81	31.49	22	0	579220	133910000	337530000
EEF1G	Elongation factor 1-gamma	P26641	2	3	11	6	2	3	11	6	44.9	44.9	50.118	64.34	22	4299300	3680800	113250000	25404000
EEF2	Elongation factor 2	P13639	17	17	25	22	16	16	24	21	40.7	39.5	95.337	245.37	93	45033000	28030000	304320000	91162000
EFHD2	EF-hand domain-containing protein D2	Q96C19	8	4	5	5	8	4	5	5	32.5	32.5	26.697	112.91	28	146080000	11229000	48903000	14335000
EFTUD2	116 kDa U5 small nuclear ribonucleoprotein component	Q15029	3	3	7	6	2	2	6	5	9.1	8	109.43	12.417	12	2580700	1888800	13875000	6705600
EHD1	EH domain-containing protein 1	Q9H4M9;C9JC03; A0A024R571;C9J2Z4	0	1	3	3	0	1	3	3	8.1	8.1	60.626	8.6007	6	0	307070	13014000	3312600
EIF2S1	Eukaryotic translation initiation factor 2 subunit 1	P05198; H0YJS4; G3V4T5	0	0	5	2	0	0	5	2	18.1	18.1	36.112	17.605	7	0	0	22867000	3452000
EIF2S3;EIF2S3L	Eukaryotic translation initiation factor 2 subunit 3;Putative eukaryotic translation initiation factor 2 subunit 3-like protein	P41091; Q2VIR3	4	4	11	9	4	4	11	9	37.1	37.1	51.109	152.53	25	6168200	7411600	86229000	25520000
EIF3A	Eukaryotic translation initiation factor 3 subunit A	Q14152	1	1	4	2	1	1	4	2	3.3	3.3	166.57	6.4578	5	1248200	1561600	6810800	2753700
EIF3B	Eukaryotic translation initiation factor 3 subunit B	P55884	0	0	4	3	0	0	4	3	7.1	7.1	92.48	8.7347	6	0	0	19683000	3218700
EIF3C;EIF3CL	Eukaryotic translation initiation factor 3 subunit C;Eukaryotic translation initiation factor 3 subunit C-like protein	Q99613; B5ME19	0	0	4	2	0	0	4	2	5.6	5.6	105.34	25.168	5	0	0	4174000	3892300

EIF3D	Eukaryotic translation initiation factor 3 subunit D	O15371; B0QYA4; B0QYA5	0	0	2	0	0	0	2	0	4.6	4.6	63.972	2.9027	2	0	0	4692100	0
EIF3E	Eukaryotic translation initiation factor 3 subunit E	P60228; H0YBR5; E5RGA2	1	0	5	3	1	0	5	3	13.5	13.5	52.22	8.2464	9	1091200	0	28959000	7420500
EIF3F	Eukaryotic translation initiation factor 3 subunit F	O00303; H0YDT6	0	0	2	1	0	0	2	1	7.8	7.8	37.563	33.56	4	0	0	5035400	2449200
EIF3G	Eukaryotic translation initiation factor 3 subunit G	O75821; K7ER90; K7EL20; K7EP16	0	0	2	2	0	0	2	2	8.4	8.4	35.611	21.946	3	0	0	4833100	2177100
EIF3I	Eukaryotic translation initiation factor 3 subunit I	Q13347	2	0	6	3	2	0	6	3	28	28	36.501	22.18	9	1381300	0	19592000	4935000
EIF3L	Eukaryotic translation initiation factor 3 subunit L	Q9Y262; B0QY90; B0QY89	0	0	2	0	0	0	2	0	4.6	4.6	66.726	22.873	2	0	0	2642400	0
EIF3M	Eukaryotic translation initiation factor 3 subunit M	Q7L2H7; E9PRI2; E9PN86; E7ESM3; J3KNJ2	0	0	2	1	0	0	2	1	9.9	9.9	42.502	60.347	3	0	0	7214000	4984700
EIF4A1	Eukaryotic initiation factor 4A-I	P60842; J3KT12;J3QS69;J3QL43;J3KTB5;J3KSZ0	16	10	17	15	7	4	9	6	42.4	18.7	46.153	300.44	116	111420000	43309000	602390000	186880000
EIF4A3	Eukaryotic initiation factor 4A-III;Eukaryotic initiation factor 4A-III, N-terminally processed	P38919;I3L3H2	10	5	12	7	7	2	9	4	30.9	24.8	46.871	18.01	19	15351000	2635600	60844000	10257000
EIF4E	Eukaryotic translation initiation factor 4E	P06730; D6RBW1	0	1	2	0	0	1	2	0	11.5	11.5	25.097	2.9105	2	0	249330	9056400	0
EIF5A;EIF5A2;EIF5AL1	Eukaryotic translation initiation factor 5A-1;Eukaryotic translation initiation factor 5A;Eukaryotic translation initiation factor 5A-2;Eukaryotic translation initiation factor 5A-1-like	P63241;I3L397;I3L504;Q9GZV4;Q6IS14;F8WCJ1;C9J7B5;C9J4W5	0	1	2	1	0	1	2	1	15.6	15.6	16.832	3.741	5	0	1375000	10089000	1623400
EIF6	Eukaryotic translation initiation factor 6	P56537; B7ZBH1	2	0	2	0	2	0	2	0	15.9	15.9	26.599	9.7701	4	1583600	0	4166000	0
ELANE	Neutrophil elastase	P08246	4	4	3	3	4	4	3	3	11.2	11.2	28.518	6.8737	17	27743000	9161900	65360000	19490000
ENO1	Alpha-enolase	P06733; A0A2R8Y6G6	10	9	16	11	10	9	16	11	61.5	61.5	47.168	323.31	127	109020000	90257000	614270000	70761000
EPPK1	Epiplakin	P58107; A0A075B730	0	1	6	0	0	1	5	0	11.4	8.8	555.65	36.027	6	0	211040	25046000	0
EPRS	Bifunctional glutamate/proline--tRNA ligase;Glutamate--tRNA ligase;Proline--tRNA ligase	P07814; V9GYZ6	18	47	156	141	18	47	155	140	85.3	85.3	170.59	323.31	3872	32705000	234900000	1.9131E+11	83254000000

ERLIN1	Erlin-1	O75477; B0QZ43	5	4	7	7	3	2	5	5	21.6	16.1	39.171	13.512	11	10429000	3800000	39256000	19954000
ERLIN2	Erlin-2	O94905; E5RHW4;E5RJ09	6	4	6	5	4	2	4	3	25.1	19.5	37.839	21.682	16	24249000	7784400	66100000	40478000
ERO1L	ERO1-like protein alpha	Q96HE7	0	0	8	0	0	0	8	0	23.1	23.1	54.392	67.299	9	0	0	57071000	0
ESYT2	Extended synaptotagmin-2	A0FGR8; A0A499FIX8;H7BXI1;A0A087WXU3	0	0	4	3	0	0	4	3	5	5	102.36	5.3039	4	0	0	7247000	2393500
ETF1	Eukaryotic peptide chain release factor subunit 1	P62495; B7Z7P8	1	0	3	2	1	0	3	2	8	8	49.03	7.6814	4	437330	0	8525100	2022900
EVPL	Envoplakin	Q92817; K7EKI0	0	0	10	0	0	0	10	0	6.2	6.2	231.6	42.125	10	0	0	21076000	0
EZR	Ezrin	P15311; E7EQR4	2	0	8	2	1	0	4	0	15	8.9	69.412	19.46	13	2591700	0	34438000	2804800
FABP5	Fatty acid-binding protein, epidermal	Q01469;I6L8B7	7	7	8	6	7	7	8	6	66.7	66.7	15.164	100.97	64	205520000	79433000	180200000	44930000
FAF2	FAS-associated factor 2	Q96CS3	0	0	4	4	0	0	4	4	21.3	21.3	52.623	13.599	8	0	0	11489000	7184500
FARSA	Phenylalanine--tRNA ligase alpha subunit	Q9Y285; K7ER00; K7ER16	2	0	4	2	2	0	4	2	8.5	8.5	57.563	9.1017	6	3207700	0	15709000	5562500
FASN	Fatty acid synthase;[Acyl-carrier-protein] S-acetyltransferase;[Acyl-carrier-protein] S-malonyltransferase;3-oxoacyl-[acyl-carrier-protein] synthase;3-oxoacyl-[acyl-carrier-protein] reductase;3-hydroxyacyl-[acyl-carrier-protein] dehydratase;Enoyl-[acyl-carrier-protein] reductase;Oleoyl-[acyl-carrier-protein] hydrolase	P49327; A0A0U1RQF0	9	4	20	8	9	4	20	8	10.9	10.9	273.42	97.233	21	7075900	1943400	49194000	2889200
FBL	rRNA 2-O-methyltransferase fibrillarin	P22087; M0R0P1; M0R299; M0QXL5; M0R2Q4;M0R2U2;M0R1H0;M0R2B0	1	2	5	4	1	2	5	4	20.2	20.2	33.784	15.931	17	2370400	1327100	32345000	17632000
FEN1	Flap endonuclease 1	P39748; F5H1Y3	0	1	1	2	0	1	1	2	7.1	7.1	42.592	29.118	3	0	702490	6564600	8279200
FERMT3	Fermitin family homolog 3	Q86UX7	6	5	11	8	6	5	11	8	24.1	24.1	75.952	65.516	32	14240000	5222300	75928000	27060000
FGA	Fibrinogen alpha chain;Fibrinopeptide A;Fibrinogen alpha chain	P02671	1	1	3	0	1	1	3	0	4.6	4.6	94.972	8.3339	4	863060	1029300	8538800	0

FGB	Fibrinogen beta chain;Fibrinopeptide B;Fibrinogen beta chain	P02675; D6REL8	1	0	3	0	1	0	3	0	11	11	55.928	6.7309	3	0	0	6403600	0
FH	Fumarate hydratase, mitochondrial	P07954	0	0	2	0	0	0	2	0	7.3	7.3	54.636	4.132	2	0	0	6644100	0
FHL3	Four and a half LIM domains protein 3	Q13643	1	1	1	2	1	1	1	2	6.1	6.1	31.192	4.2827	5	7271700	4934500	5411300	5212400
FLNA	Filamin-A	P21333; Q60FE5; A0A087WWY3	48	20	43	32	48	20	42	32	30.5	30.2	280.74	323.31	109	140950000	86747000	119950000	43135000
FLNB	Filamin-B	O75369; E7EN95	1	1	9	0	1	1	8	0	4.5	4.2	278.16	21.858	9	1055800	576990	19570000	0
FSCN1	Fascin	Q16658	0	0	4	0	0	0	4	0	9.1	9.1	54.529	7.0821	4	0	0	9958400	0
FXR1	Fragile X mental retardation syndrome-related protein 1	P51114; B4DXZ6; E7EU85; E9PFF5	0	0	3	3	0	0	3	3	6.8	6.8	69.72	7.0139	6	0	0	6191000	3733800
G6PD	Glucose-6-phosphate 1-dehydrogenase	P11413; E9PD92; E7EM57; E7EUI8	1	0	2	0	1	0	2	0	3.9	3.9	59.256	2.1507	2	747630	0	4109800	0
GALK1	Galactokinase	P51570	3	1	9	8	3	1	9	8	32.1	32.1	42.272	62.802	21	2789200	986270	36146000	18230000
GAPDH	Glyceraldehyde-3-phosphate dehydrogenase	P04406; E7EUT5	19	15	19	20	19	15	19	20	81.2	81.2	36.053	323.31	374	847390000	582320000	2993700000	1500300000
GARS	Glycine--tRNA ligase	P41250; A0A6Q8PGW4;A0A6Q8PGI6;A0A6Q8PGA8;A0A6Q8PHH9;A0A6Q8PGZ8;A0A6Q8PHI7;H7C443; A0A6Q8PFZ6	6	5	18	17	6	5	18	17	33.3	33.3	83.165	175.56	50	13162000	12536000	146350000	83741000
GART	Trifunctional purine biosynthetic protein adenosine-3;Phosphoribosylamine--glycine ligase;Phosphoribosylformylglycinamidine cyclo-ligase;Phosphoribosylglycinamide formyltransferase	P22102	8	8	16	15	8	8	16	15	26.1	26.1	107.77	76.791	25	12622000	9739600	59387000	42984000
GFPT1	Glutamine--fructose-6-phosphate aminotransferase [isomerizing] 1	Q06210; A0A6I8PRN4;A0A6I8PTT9	1	0	3	3	1	0	3	3	5.3	5.3	78.806	9.0756	5	430910	0	2950300	3688300
GGCT	Gamma-glutamylcyclotransferase	O75223; M0QZK8;B8ZZK2	3	4	3	3	3	4	3	3	23.9	23.9	21.007	16.47	25	23112000	18382000	12714000	16677000

GLUL	Glutamine synthetase	P15104;A0A2R8YDT1	0	0	2	0	0	0	2	0	7.5	7.5	42.064	9.9451	3	0	0	10839000	0
GM2A	Ganglioside GM2 activator;Ganglioside GM2 activator isoform short	P17900	2	2	2	1	2	2	2	1	16.1	16.1	20.838	8.3431	11	13068000	6494200	21895000	3042000
GNAI2	Guanine nucleotide-binding protein G(i) subunit alpha-2	P04899	2	1	3	2	2	1	2	1	11.3	7.9	40.45	9.9882	7	1702200	378320	6544000	2080900
GNAS	Guanine nucleotide-binding protein G(s) subunit alpha isoforms short;Guanine nucleotide-binding protein G(s) subunit alpha isoforms XLas	P63092;Q5JWF2;A0A0A0MR13;A0A590UJ58;B0AZR9;A0A590UJF0;A0A590UJB0;A0A590UJY2;A0A590UJR6;A0A590UJI6;A0A590UJQ9;A0A590UKB4;A0A590UJJ0;A0A590UJG5	0	0	3	1	0	0	3	1	8.9	8.9	45.664	3.4108	3	0	0	6197100	2438800
GNB1;GNB2;GNB4	Guanine nucleotide-binding protein G(I)/G(S)/G(T) subunit beta-1;Guanine nucleotide-binding protein G(I)/G(S)/G(T) subunit beta-2;Guanine nucleotide-binding protein subunit beta-4	F6X3N5;F6UT28;B1AKQ8;P62873;P62879;A0A6Q8PFW8;A0A6Q8PFV8;A0A6Q8PFE0;A0A6Q8PG88;E7EP32;C9JXA5;H7C5J5;C9JIS1;C9JZN1;Q9HAV0	1	1	3	1	1	1	3	1	21.5	21.5	18.34	5.3181	3	2495800	1086100	16079000	2498900
GNB2L1	Guanine nucleotide-binding protein subunit beta-2-like 1;Guanine nucleotide-binding protein subunit beta-2-like 1, N-terminally processed	P63244;J3KPE3;D6RAC2;D6RHH4;D6REE5;H0YAF8;D6R9L0;H0Y8W2;D6R9Z1;H0YAM7;D6RFX4;D6RBD0	13	10	17	17	13	10	17	17	78.9	78.9	35.076	323.31	106	123590000	72265000	649200000	349950000
GPI	Glucose-6-phosphate isomerase	P06744;A0A2U3TZU2;K7	1	1	9	1	1	0	6	1	23.3	16.5	63.146	102.37	10	558780	371840	68826000	235420

		EQ48;A0A2R8Y6C7;A0A0J9YYH3;A0A0J9YXP8;A0A0J9YX90																	
GRB2	Growth factor receptor-bound protein 2	P62993;J3KT38	0	1	3	2	0	1	3	2	22.1	22.1	25.206	7.6272	4	0	182060	4653500	1944700
GRPEL1	GrpE protein homolog 1, mitochondrial	Q9HAV7	10	11	9	10	10	11	9	10	54.8	54.8	24.279	285.29	61	424550000	308070000	480850000	276540000
GRWD1	Glutamate-rich WD repeat-containing protein 1	Q9BQ67;M0QX71	0	1	5	5	0	1	5	5	15.7	15.7	49.419	15.296	11	0	1512800	16741000	19191000
GSDMA	Gasdermin-A	Q96QA5;J3KRG2	4	5	7	6	4	5	7	6	21.3	21.3	49.364	157.27	48	37178000	25974000	80209000	22316000
GSTK1	Glutathione S-transferase kappa 1	Q9Y2Q3;E9PFN5;C9JNT3	0	0	3	1	0	0	3	1	19	19	25.497	13.574	4	0	0	15101000	5172200
GSTP1	Glutathione S-transferase P	P09211;A8MX94;A0A087X2E9	3	5	6	2	3	5	6	2	41	41	23.356	123.63	13	6328100	7941900	119200000	2444400
GTF2I	General transcription factor II-I	P78347;A0A494C1K3	0	0	3	1	0	0	3	1	7	7	112.42	5.7134	4	0	0	5374500	1312400
HADHB	Trifunctional enzyme subunit beta, mitochondrial;3-ketoacyl-CoA thiolase	P55084;B5MD38;F5GZQ3;C9JEY0;C9JE81	3	2	10	7	3	2	10	7	23.4	23.4	51.294	39.161	23	4583400	5784500	122490000	39460000
HAL	Histidine ammonia-lyase	P42357	4	9	4	3	4	9	4	3	16.9	16.9	72.697	21.043	28	18562000	37058000	27370000	1351700
HAT1	Histone acetyltransferase type B catalytic subunit	O14929	0	1	2	0	0	1	2	0	7.6	7.6	49.512	2.0779	2	0	399230	4360800	0
HAX1	HCLS1-associated protein X-1	O00165;E9PIQ7;Q5VYD6	1	0	2	2	1	0	2	2	7.9	7.9	31.62	3.726	3	370110	0	4148200	3539000
HBB	Hemoglobin subunit beta;LVV-hemorphin-7;Spinorphin	P68871;F8W6P5;A0A2R8Y7R2	7	2	3	3	6	1	2	2	36.7	36.7	15.998	73.041	168	9715900000	11029000	27913000	65210000
HDAC1;HDAC2	Histone deacetylase 1;Histone deacetylase 2	Q13547;Q5TEE2;Q92769	0	0	2	1	0	0	2	1	7.1	7.1	55.102	3.1492	3	0	0	2111600	0
HIST1H2AG;H2AFJ;HIST2H2AC;HIST1H2AJ;HIST1H2AC;HIST3H2A;HIST1H2AB;HIST2H2AA3;HIST1H2AH;HIST1H2AD;H2AFX;H2AFV;HIST	Histone H2A type 1;Histone H2A.J;Histone H2A type 2-C;Histone H2A type 1-J;Histone H2A type 1-C;Histone H2A type 3;Histone H2A type 1-B/E;Histone H2A type 2-A;Histone H2A type 1-H;Histone H2A type 1-D;Histone H2AX;Histone H2A;Histone H2A.V;Histone H2A type 1-A;Histone H2A.Z	P0C0S8;A0A0U1RR32;Q9BTM1;Q16777;Q99878;Q93077;Q7L7L0;P04908;Q6FI13;Q96KK5;A0A0U1RRH7;P20671;P16104;A0A494C189;A0A3B3I	3	3	3	3	3	3	3	3	26.9	26.9	14.091	6.5576	28	46676000	23712000	145480000	36777000

1H2AA;H2AFZ		S11;C9J0D1;Q71UI9;H0YFX9;Q96QV6;P0C0S5																	
HIST1H4A	Histone H4	P62805	4	4	6	4	4	4	6	4	42.7	42.7	11.367	48.521	52	98845000	44711000	371410000	23418000
HK2	Hexokinase-2;Hexokinase	P52789; E9PB90	0	0	5	3	0	0	5	3	7.4	7.4	102.38	16.294	10	0	0	15254000	4576200
HMGCS1	Hydroxymethylglutaryl-CoA synthase, cytoplasmic	Q01581	0	0	3	0	0	0	3	0	6.9	6.9	57.293	5.9992	3	0	0	9292200	0
HMOX1	Heme oxygenase 1	P09601; B1AHA8	1	0	2	0	1	0	2	0	8.3	8.3	32.818	4.4407	3	611500	0	8702000	0
HNRNPA0	Heterogeneous nuclear ribonucleoprotein A0	Q13151	2	0	2	3	2	0	2	3	16.7	16.7	30.84	39.276	6	2278300	0	14437000	18793000
HNRNPA2B1	Heterogeneous nuclear ribonucleoproteins A2/B1	P22626; A0A087WUI2	0	0	3	2	0	0	3	2	11.3	11.3	37.429	9.2599	5	0	0	30790000	1318000
HNRNPA3	Heterogeneous nuclear ribonucleoprotein A3	P51991	4	3	7	2	4	3	7	2	28	28	39.594	21.572	21	9280100	2661200	60033000	6427900
HNRNPC	Heterogeneous nuclear ribonucleoproteins C1/C2	P07910; G3V576; G3V4W0;B4DY08;B2R5W2;G3V4C1;G3V2Q1;G3V4M8;G3V5X6;G3V3K6;G3V251;B4DSU6;G3V555;G3V5V7;G3V575;G3V2D6	0	0	2	0	0	0	2	0	7.2	7.2	33.67	4.3395	2	0	0	6635100	0
HNRNPH1;HNRNPH2	Heterogeneous nuclear ribonucleoprotein H;Heterogeneous nuclear ribonucleoprotein H, N-terminally processed;Heterogeneous nuclear ribonucleoprotein H2	P31943; E9PCY7; G8JLB6; D6RBM0;D6RIU0; D6RFM3;D6RIH9; D6RJ04; D6RDU3;P55795; D6RIT2; D6R9T0; E5RGV0; D6RAM1;E7EQJ0;E7EN40	4	3	5	5	3	3	4	4	16.7	14.5	49.229	123.14	22	14650000	6799100	81008000	31123000
HNRNPK	Heterogeneous nuclear ribonucleoprotein K	P61978; Q5T6W2	0	1	6	2	0	1	6	2	18.6	18.6	50.976	35.788	9	0	1536900	22127000	3166800
HNRNPL	Heterogeneous nuclear ribonucleoprotein L	P14866; M0QXS5	0	0	2	1	0	0	2	1	10.4	10.4	64.132	7.38	3	0	0	4430600	1589900

		;A0A3B3ITJ4																	
HNRNPM	Heterogeneous nuclear ribonucleoprotein M	P52272; A0A087X0X3	8	4	10	8	8	4	10	8	18.8	18.8	77.515	63.507	43	27096000	16508000	118920000	55177000
HNRNPU	Heterogeneous nuclear ribonucleoprotein U	Q00839; Q5RI18; A0A1W2PP35;A0A1W2PPS1;A0A1W2PQL0;A0A1W2PP34;A0A1W2PPL4;A0A1W2PPH7;A0A1X7SBS1;A0A1W2PRZ7;A0A1W2PQ74;A0A1W2PQD4	6	5	11	8	6	5	11	8	15.8	15.8	90.583	106.56	45	37272000	26743000	214270000	65853000
HNRNPUL1	Heterogeneous nuclear ribonucleoprotein U-like protein 1	Q9BUJ2; M0R3F1; A0A0A0MRA5;B7Z4B8;M0QYM5; M0QYI8; M0QYZ0	4	4	3	1	4	4	3	1	5	5	95.737	11.228	11	10773000	7954600	9733700	1503000
HP1BP3	Heterochromatin protein 1-binding protein 3	Q5SSJ5; B0QZK4; Q5SWC8	2	2	9	2	2	2	9	2	18.6	18.6	61.206	58.215	13	1224700	833460	48415000	9680600
HSD17B10	3-hydroxyacyl-CoA dehydrogenase type-2	Q99714; Q5H928	2	0	5	3	2	0	5	3	35.2	35.2	26.923	106.82	8	3247900	0	23937000	6988000
HSP90AA1	Heat shock protein HSP 90-alpha	P07900	13	10	21	22	5	3	9	10	29	12.2	84.659	105.8	31	9084700	7551200	52545000	25180000
HSP90AB1	Heat shock protein HSP 90-beta	P08238	20	17	27	27	9	8	12	12	37	16.7	83.263	323.31	145	147800000	135790000	790160000	447990000
HSP90B1	Endoplasmin	P14625	5	3	8	5	3	2	6	3	14.4	12.7	92.468	19.019	13	5846500	2781600	29508000	6755800
HSPA1A;HSPA1B	Heat shock 70 kDa protein 1A;Heat shock 70 kDa protein 1B	P0DMV8;A0A0G2JIW1;P0DMV9;V9GZ37	7	7	14	10	3	4	6	6	24.5	9.8	70.051	106.45	40	14955000	13497000	143680000	36142000
HSPA5	78 kDa glucose-regulated protein	P11021	24	23	26	26	23	22	25	25	47.1	47.1	72.332	323.31	193	378670000	316040000	471110000	326080000
HSPA8	Heat shock cognate 71 kDa protein	P11142; E9PKE3; E9PN89; E9PNE6	23	23	24	23	20	20	21	20	46.3	42.9	70.897	323.31	225	475510000	466480000	1041500000	561510000
HSPA9	Stress-70 protein, mitochondrial	P38646	20	20	22	22	20	20	22	22	46.1	46.1	73.68	323.31	161	376620000	358850000	642240000	444700000
HSPB1	Heat shock protein beta-1	P04792; A0A6Q8PFK8;A0A6Q8PGK1;A0A6Q8PHJ6;	9	7	12	8	9	7	12	8	85.9	85.9	22.782	323.31	99	250350000	63927000	749460000	215470000

		A0A6Q8PFE7;A0A6Q8PF43;A0A6Q8PHA6;A0A6Q8PH65;A0A6Q8PGY2																	
HSPD1	60 kDa heat shock protein, mitochondrial	P10809	11	8	20	20	11	8	20	20	49	49	61.054	321.86	98	47727000	36609000	510630000	207430000
HUWE1	E3 ubiquitin-protein ligase HUWE1	Q7Z6Z7;A0A087X1S3;H0Y659	0	0	2	0	0	0	2	0	0.6	0.6	481.89	2.021	2	0	0	1871300	0
HYOU1	Hypoxia up-regulated protein 1	Q9Y4L1;A0A087X054;A0A494C039;E9PJ21;A0A087WWI4;K7EQK2	5	5	12	12	5	5	12	12	19.6	19.6	111.33	101.74	28	5070900	4161500	45032000	42418000
IARS	Isoleucine--tRNA ligase, cytoplasmic	P41252;A0A0A0MSX9;J3KR24	5	2	50	46	5	2	50	46	50.7	50.7	144.5	323.31	262	4802100	4031800	1894300000	1088900000
IARS2	Isoleucine--tRNA ligase, mitochondrial	Q9NSE4	0	0	2	1	0	0	2	1	1.9	1.9	113.79	2.3952	3	0	0	3325200	849780
IDH2	Isocitrate dehydrogenase [NADP], mitochondrial	P48735	11	8	15	15	10	8	14	14	43.4	41.4	50.909	285.24	76	46421000	23131000	187990000	89651000
IDH3A	Isocitrate dehydrogenase [NAD] subunit alpha, mitochondrial	P50213;H0YKD0;H0YLI6;H0YL72;H0YM64;H0YMU3	0	0	2	1	0	0	2	1	5.5	5.5	39.591	3.7249	3	0	0	7364900	1511000
IDH3B	Isocitrate dehydrogenase [NAD] subunit beta, mitochondrial;Isocitrate dehydrogenase [NAD] subunit, mitochondrial	O43837;A0A087WZN1;A0A087X2E5	2	2	4	5	2	2	4	5	15.6	15.6	42.183	14.784	10	2102700	2183400	17673000	10882000
IGF2BP3	Insulin-like growth factor 2 mRNA-binding protein 3	O00425	0	0	2	1	0	0	2	1	4.7	4.7	63.704	10.388	3	0	0	4447200	1530000
IGHA1	Ig alpha-1 chain C region	P01876;A0A286YEY1	10	9	7	7	3	2	3	3	32.3	10.2	37.654	142.22	89	706470000	582210000	231800000	182750000
IGHG1	Ig gamma-1 chain C region	P01857;A0A0A0MS08;A0A0A0MS07	4	6	13	7	3	4	6	4	59.4	31.5	36.105	201.5	91	46706000	40934000	896190000	37380000
IGHG2	Ig gamma-2 chain C region	P01859;A0A286YEY4	2	4	9	3	0	1	2	0	35.3	10.1	35.9	145.61	13	452940	7731600	187260000	2624900
IGHM	Ig mu chain C region	P01871	4	5	2	1	4	5	2	1	16.6	16.6	49.439	102.31	12	11878000	22728000	4853600	1480800
IGJ;JCHAIN	Immunoglobulin J chain	P01591;C9JA05;	4	3	3	3	4	3	3	3	18.9	18.9	18.098	27.194	28	114330000	79614000	67570000	26039000

		D6RHJ6; D6RD17																
IGKC	Ig kappa chain C region	P01834; A0A5H1ZRQ3	7	6	8	2	7	6	8	2	83.2	83.2	11.765	323.31	76	180060000	286040000	660550000	45102000
IGKV3D-20	Ig kappa chain V-III region B6	P01619; A0A0C4DH25	1	2	1	0	1	2	1	0	21.6	21.6	12.557	40.349	7	4898500	9384900	25126000	0
IGLC6;IGLC7	Ig lambda-6 chain C region;Ig lambda-7 chain C region	P0DOY3; P0DOY2; P0CF74; A0A5H1ZRQ7;A0M8Q6	6	5	6	2	2	1	2	0	85.8	34	11.265	207.48	63	96118000	89692000	224380000	21650000
IGLL5;IGLC1	Immunoglobulin lambda-like polypeptide 5;Ig lambda-1 chain C regions	B9A064; A0A0B4J231;A0A5H1ZRQ4;P0CG04	6	6	6	2	2	2	2	0	38.3	12.6	23.063	13.745	6	495160	5590300	18610000	0
IL1RN	Interleukin-1 receptor antagonist protein	P18510	0	0	5	0	0	0	5	0	34.5	34.5	20.055	78.616	5	0	0	30720000	0
IMMT	MICOS complex subunit MIC60	Q16891; H7C463; C9J406; B9A067	6	3	10	9	6	3	10	9	21.1	21.1	83.677	65.721	22	8004200	3604300	44823000	21301000
IMPDH2	Inosine-5-monophosphate dehydrogenase 2	P12268; H0Y4R1	3	0	2	1	3	0	2	1	10.7	10.7	55.804	7.1611	5	5729800	0	5966500	1637700
INPP5D	Phosphatidylinositol 3,4,5-trisphosphate 5-phosphatase 1	Q92835; H0Y5Q9	1	1	2	1	1	1	2	1	2	2	133.29	3.2755	3	184710	76215	3856200	860050
IPO5	Importin-5	O00410; H0Y8C6; E7ETV3	0	0	3	3	0	0	3	3	3.9	3.9	123.63	4.6784	4	0	0	11155000	4847200
IQGAP1	Ras GTPase-activating-like protein IQGAP1	P46940; A0A0J9YXZ5;H0YLE8	30	12	36	20	29	12	36	19	29.1	28.5	189.25	323.31	90	130040000	23070000	153110000	60539000
ITPA	Inosine triphosphate pyrophosphatase	Q9BY32	3	3	5	5	3	3	5	5	39.7	39.7	21.445	59.594	10	5622900	3710600	28993000	20771000
IVL	Involucrin	P07476	3	0	8	0	3	0	8	0	19.3	19.3	68.478	47.01	9	5163700	0	25127000	0
JUP	Junction plakoglobin	P14923	36	25	31	33	33	22	28	30	56.2	51.7	81.744	323.31	409	1266400000	570800000	1104100000	538470000
KARS	Lysine--tRNA ligase	Q15046	2	3	19	16	2	3	19	16	43	43	68.047	152.08	54	1850000	2710200	210870000	95824000
KHDRBS1	KH domain-containing, RNA-binding, signal transduction-associated protein 1	Q07666	0	0	2	0	0	0	2	0	6.3	6.3	48.227	1.8124	2	0	0	2243500	0
KHSRP	Far upstream element-binding protein 2	Q92945; A0A087WTP3	3	1	7	6	3	1	7	6	11.3	11.3	73.114	13.929	10	1650400	307240	15322000	10457000
KIF15	Kinesin-like protein KIF15;Kinesin-like protein	Q9NS87; D6RCT7; C9JKA9; H7C1K7	1	2	1	1	1	2	1	1	2.4	2.4	160.16	2.9725	5	50697000	59858000	82728000	65996000
KLK5	Kallikrein-5	Q9Y337; M0QXX2	0	1	1	0	0	1	1	0	11.3	11.3	32.02	11.325	2	0	1233300	4161800	0
KLK7	Kallikrein-7	P49862	2	2	2	2	2	2	2	2	7.1	7.1	27.524	3.6776	13	1734300	4472500	17078000	2705200

KPNB1	Importin subunit beta-1	Q14974; J3QR48; J3KTM9	3	1	5	5	3	1	5	5	8.6	8.6	97.169	31.83	12	1647900	257310	21112000	13883000
LACRT	Extracellular glycoprotein lacritin	Q9GZZ8; F8W0V3	2	2	2	0	2	2	2	0	16.7	16.7	14.246	4.45	6	11285000	17929000	7575800	0
LANCL1	LanC-like protein 1	O43813; E9PHS0; F8WDS9	2	0	3	2	2	0	3	2	13.5	13.5	45.283	6.6652	5	849050	0	6039900	2730600
LAP3	Cytosol aminopeptidase	P28838; H0Y9Q1	1	0	2	0	1	0	2	0	4.6	4.6	56.166	3.0961	2	631880	0	1147600	0
LARS	Leucine--tRNA ligase, cytoplasmic	Q9P2J5; A0A6I8PL42;B4DER1;A0A6I8PIT3; A0A6I8PIV1;A0A6I8PS05; A0A6I8PRS0;A0A6I8PLB3; A0A6I8PLB8;A0A6I8PIP7	3	1	18	13	3	1	18	13	19.6	19.6	134.46	113.26	36	3898500	486480	99351000	58306000
LBR	Lamin-B receptor	Q14739; A0A494C1L1;C9JXK0	1	0	2	0	1	0	2	0	3.1	3.1	70.702	2.9862	3	3455000	0	4675500	0
LCN1	Lipocalin-1	P31025	9	8	6	5	9	8	6	5	47.7	47.7	19.25	45.032	62	325880000	201350000	190010000	80295000
LCN2	Neutrophil gelatinase-associated lipocalin	P80188; X6R8F3	1	1	2	1	1	1	2	1	15.2	15.2	22.588	132.72	6	5039900	1921700	12210000	1185300
LCP1	Plastin-2	P13796	1	2	5	0	1	2	4	0	11.3	9.9	70.288	11.453	6	1208300	2271500	17565000	0
LDHA	L-lactate dehydrogenase A chain	P00338	9	6	12	9	8	5	11	8	41.9	38.3	36.688	77.814	64	76261000	20433000	347470000	58412000
LDHB	L-lactate dehydrogenase B chain	P07195; A0A5F9ZHM4;A0A3B3IS95;A8MW50	7	4	9	9	6	3	8	8	32	28.4	36.638	23.412	19	8550700	3641000	40143000	20007000
LGALS3	Galectin-3	P17931	0	0	2	0	0	0	2	0	10	10	26.152	5.1112	2	0	0	10356000	0
LGALS7	Galectin-7	P47929	8	8	9	8	8	8	9	8	82.4	82.4	15.075	118.02	104	131950000	67232000	870510000	36628000
LIG3	DNA ligase 3	P49916; K7ERZ5	0	1	2	2	0	1	2	2	2.7	2.7	112.91	2.4717	1	0	132530	1699400	1288900
LMAN1	Protein ERGIC-53	P49257	0	0	2	1	0	0	2	1	3.7	3.7	57.548	1.9423	3	0	0	2982200	0
LMNA	Prelamin-A/C;Lamin-A/C	P02545; A0A6Q8PFJ0;A0A6Q8PHQ9;Q3BDU5;Q5TCI8;A0A6Q8PF80	17	13	30	25	16	13	30	25	53.9	52.7	74.139	323.31	109	83889000	23495000	382510000	68792000

LMNB1	Lamin-B1	P20700; E9PBF6; A0A0D9SFE5	8	9	14	14	7	9	14	14	36.9	35.5	66.408	323.31	33	7563200	8555200	100030000	39868000
LMNB2	Lamin-B2	Q03252	2	2	2	2	1	2	2	2	8.2	6.9	69.948	5.2686	3	400160	273310	4463600	2074000
LONP1	Lon protease homolog, mitochondrial	P36776; K7EJE8; K7EKE6; K7ER27	1	1	8	8	1	1	8	8	10.3	10.3	106.49	21.396	12	1731100	1285600	24606000	14137000
LOR	Loricrin	P23490	2	2	3	3	2	2	3	3	6.7	6.7	25.76	7.3504	45	47355000	26945000	56117000	30778000
LPXN	Leupaxin	O60711; B7Z5P7	2	2	3	2	2	2	3	2	12.2	12.2	43.332	8.4733	7	1932400	2321800	5707100	3613500
LRPPRC	Leucine-rich PPR motif-containing protein, mitochondrial	P42704	2	3	7	6	2	3	7	6	8.2	8.2	157.9	17.749	23	131360000	122900000	183630000	93220000
LRRC40	Leucine-rich repeat-containing protein 40	Q9H9A6	0	0	1	1	0	0	1	1	3.2	3.2	68.249	1.8577	2	0	0	2995200	874090
LRRC47	Leucine-rich repeat-containing protein 47	Q8N1G4	0	0	2	1	0	0	2	1	6.7	6.7	63.472	7.3171	3	0	0	1569600	1417800
LTF	Lactotransferrin;Lactoferricin-H;Kaliocin-1;Lactoferroxin-A;Lactoferroxin-B;Lactoferroxin-C	P02788; E7EQB2; E7ER44	36	25	31	14	34	24	29	13	62.5	59.6	78.181	323.31	149	247740000	179890000	290350000	40520000
LYPLAL1	Lysophospholipase-like protein 1	Q5VWZ2	0	0	2	1	0	0	2	1	8.4	8.4	26.316	2.1776	3	0	0	4061900	0
LYZ	Lysozyme C;Lysozyme	P61626; F8VV32; A0A0B4J259	5	5	5	4	5	5	5	4	52.7	52.7	16.537	157.79	50	292870000	182820000	234250000	127720000
MAGED2	Melanoma-associated antigen D2	Q9UNF1; Q5H909; Q5H907	15	17	18	14	15	17	18	14	38.8	38.8	64.953	323.31	144	235690000	243600000	345120000	116950000
MAP2K1	Dual specificity mitogen-activated protein kinase kinase 1	Q02750	2	2	3	0	2	1	2	0	8.1	5.9	43.439	2.2988	3	2342000	657940	8713800	0
MAP2K2	Dual specificity mitogen-activated protein kinase kinase 2	P36507	1	1	7	2	1	0	6	2	32.5	30.2	44.424	28.147	8	792260	1042900	20311000	7009000
MAPRE2;MAPRE3	Microtubule-associated protein RP/EB family member 2;Microtubule-associated protein RP/EB family member 3	Q15555; Q9UPY8; K7ENB3; M0QX52; K7EL66; K7ERD8; C9JB30	0	1	2	0	0	1	2	0	8.9	8.9	37.031	8.0319	2	0	262500	2125000	0
MARS	Methionine--tRNA ligase, cytoplasmic	P56192	1	1	19	16	1	1	19	16	32.1	32.1	101.11	305.59	47	688060	197890	175630000	76154000
MCCC1	Methylcrotonoyl-CoA carboxylase subunit alpha, mitochondrial	Q96RQ3; E9PHF7; F5GYT8; E9PG35; G5E9X5	31	31	33	33	31	31	33	33	67	67	80.472	323.31	965	6483400000	4538200000	7129300000	3282700000
MCCC2	Methylcrotonoyl-CoA carboxylase beta chain, mitochondrial	Q9HCC0;D6RDF7	36	34	36	35	17	16	17	15	75.8	37.5	61.332	323.31	797	9295800000	7005500000	9849800000	4743300000
MCM2	DNA replication licensing factor MCM2	P49736; H0Y8E6	2	0	5	5	2	0	5	5	8.6	8.6	101.89	11.946	9	4882500	0	19781000	8978900

MCM3	DNA replication licensing factor MCM3	P25205; J3KQ69; A0A499FHX9	8	6	18	18	8	6	18	18	31.8	31.8	90.98	216.03	37	13115000	7918700	106060000	65768000
MCM4	DNA replication licensing factor MCM4	P33991; A0A3B3IU45;A0A3B3IT92; A0A3B3IS57;E5RG31;A0A3B3ITP6	17	13	24	22	17	13	24	22	34.2	34.2	96.557	269.96	85	45802000	31742000	247120000	128590000
MCM5	DNA replication licensing factor MCM5;DNA helicase	P33992; B1AHB1	7	7	13	11	7	7	13	11	22.2	22.2	82.285	34.423	33	13326000	5630400	57651000	60263000
MCM6	DNA replication licensing factor MCM6	Q14566	7	5	16	17	7	5	16	17	28.1	28.1	92.888	251.15	61	9192300	6216800	145720000	69548000
MCM7	DNA replication licensing factor MCM7	P33993	14	11	24	26	14	11	24	26	49.4	49.4	81.307	175.44	97	46423000	35547000	294560000	179770000
MCU	Calcium uniporter protein, mitochondrial	Q8NE86; S4R468	0	0	2	1	0	0	2	1	8	8	39.866	2.7034	3	0	0	2028800	0
MDH1	Malate dehydrogenase, cytoplasmic;Malate dehydrogenase	P40925; A0A5K1VW95;B9A041;C9JF79;B8ZZ51	1	0	5	0	1	0	5	0	21.3	21.3	36.426	12.371	5	766840	0	15284000	0
MDH2	Malate dehydrogenase, mitochondrial;Malate dehydrogenase	P40926; G3XAL0	1	1	6	1	1	1	6	1	23.7	23.7	35.503	10.998	10	3628100	147940	36476000	840380
MGST1	Microsomal glutathione S-transferase 1	P10620; F5H7F6; F5H6X2; F5H760	0	1	1	2	0	1	1	2	18.7	18.7	17.598	5.3189	3	0	2362000	1515500	13174000
MLEC	Malectin	Q14165; F5H1S8; F5GX14	1	2	5	5	1	2	5	5	27.1	27.1	32.233	56.968	17	1396000	1376100	26385000	18159000
MMS19	MMS19 nucleotide excision repair protein homolog	Q96T76; Q5T454	0	1	3	2	0	1	3	2	3.8	3.8	113.29	6.5039	7	0	634560	8280700	2662800
MNDA	Myeloid cell nuclear differentiation antigen	P41218	0	1	2	1	0	1	2	1	6.6	6.6	45.836	2.3977	1	0	1526100	5470700	819900
MPO	Myeloperoxidase;Myeloperoxidase;89 kDa myeloperoxidase;84 kDa myeloperoxidase;Myeloperoxidase light chain;Myeloperoxidase heavy chain	P05164; J3QSF7	7	2	3	0	7	2	3	0	10.3	10.3	83.868	62.862	12	26854000	1461900	4468700	0
MRTO4	mRNA turnover protein 4 homolog	Q9UKD2	0	0	2	2	0	0	2	2	16.3	16.3	27.56	5.4271	3	0	0	4104000	4547300
MSH2	DNA mismatch repair protein Msh2	P43246; A0A2R8YFH0;E9PHA6;A0A2R8Y6P0;A0A2R8YG02; A0A2R8Y7S8	3	2	3	4	3	2	3	4	6	6	104.74	6.9478	4	2030700	664340	5929800	4639400

MSN;RDX	Moesin;Radixin	P26038; P35241; A0A2R8Y7M3;A0A2R8Y5S7	3	1	7	5	2	1	3	3	12.3	6.1	67.819	14.589	6	2580800	2723300	30340000	4590400
MTHFD1	C-1-tetrahydrofolate synthase, cytoplasmic;Methylenetetrahydrofolate dehydrogenase;Methenyltetrahydrofolate cyclohydrolase;Formyltetrahydrofolate synthetase;C-1-tetrahydrofolate synthase, cytoplasmic, N-terminally processed	P11586; V9GYY3; A0A384N5Y3;F5H2F4;V9GZ78;A0A494C1T2	9	4	23	23	9	4	23	23	28.9	28.9	101.56	134.77	47	12576000	3442200	125590000	56945000
MYH14	Myosin-14	Q7Z406; A1L2Z2	5	3	8	1	2	1	4	1	4.8	2.2	227.87	8.5232	4	4239800	2846800	13394000	215120
MYH9	Myosin-9	P35579	48	42	63	9	45	40	59	9	37	34.3	226.53	323.31	188	142010000	102120000	526460000	5351100
MYL6	Myosin light polypeptide 6	P60660; F8VPF3; F8W1R7; J3KND3; G8JLA2; G3V1V0; B7Z6Z4; F8VZU9; G3V1Y7; F8W180	0	1	3	1	0	1	3	1	24.5	24.5	16.93	10.699	3	0	1346400	21436000	622950
MYO1F; MYO1E	Unconventional myosin-If;Unconventional myosin-Ie	O00160; Q12965	0	0	2	3	0	0	2	3	3.7	3.7	124.84	17.622	7	0	0	5799400	7447600
MYO1G	Unconventional myosin-Ig;Minor histocompatibility antigen HA-2	B0I1T2;A0A3B3IU30	26	18	24	19	26	18	24	19	42.1	42.1	116.44	323.31	77	148570000	30412000	90580000	30641000
NACA	Nascent polypeptide-associated complex subunit alpha, muscle-specific form;Nascent polypeptide-associated complex subunit alpha	E9PAV3; F8W1N5; F8VZJ2; F8VNW4;F8W0W4;H0YHX9;Q13765	0	0	2	1	0	0	2	1	1.4	1.4	205.42	21.232	5	0	0	11494000	1536400
NAMPT	Nicotinamide phosphoribosyltransferase	P43490; A0A0C4DFS8	0	1	8	5	0	1	8	5	28.9	28.9	55.52	56.591	13	0	3147700	38084000	12334000
NCAPD2	Condensin complex subunit 1	Q15021; E7EN77	1	1	2	2	1	1	2	2	3.4	3.4	157.18	2.754	3	315850	307460	1027600	7906200
NCCRP1	F-box only protein 50	Q6ZVX7	4	3	3	3	4	3	3	3	18.9	18.9	30.847	117.29	40	46320000	21257000	87451000	28392000
NCL	Nucleolin	P19338; H7BY16	2	3	14	13	2	3	14	13	22.1	22.1	76.613	164.79	31	1768500	3253700	212750000	102860000
NDRG1	Protein NDRG1	Q92597; E5RJY1; E7ESM1	2	0	2	3	2	0	2	3	14	14	42.835	41.759	9	6046900	0	10055000	10128000
NDUFS1	NADH-ubiquinone oxidoreductase 75	P28331; B4DJ81	1	0	3	3	1	0	3	3	7.6	7.6	79.467	15.85	5	1016500	0	21548000	4919000

	kDa subunit, mitochondrial																	
NDUFS3	NADH dehydrogenase [ubiquinone] iron-sulfur protein 3, mitochondrial	O75489	1	2	4	3	1	2	4	3	18.9	18.9	30.241	8.1424	6	2127400	1736400	14882000	5621300
NDUFV1	NADH dehydrogenase [ubiquinone] flavoprotein 1, mitochondrial	P49821; G3V0I5; E9PQP1; B4DE93	0	1	3	1	0	1	3	1	6.9	6.9	50.817	5.6362	4	0	721220	13315000	1217400
NOC2L	Nucleolar complex protein 2 homolog	Q9Y3T9	0	0	2	3	0	0	2	3	3.6	3.6	84.918	3.8235	6	0	0	0	1178700
NONO	Non-POU domain-containing octamer-binding protein	Q15233; C9IZL7; H7C367	3	2	5	3	2	2	4	2	15.1	13.4	54.231	28.399	15	1313100	2065800	52199000	11507000
NOP56	Nucleolar protein 56	O00567; A0A494C128;H0Y653;H0YDU4;Q5JXT2	0	0	2	2	0	0	2	2	4	4	66.049	4.3175	6	0	0	4787100	2048800
NOP58	Nucleolar protein 58	Q9Y2X3	1	1	4	3	1	1	4	3	10	10	59.578	8.4556	8	943190	485770	11756000	4813900
NPEPPS;NPEPPSL1	Puromycin-sensitive aminopeptidase;Puromycin-sensitive aminopeptidase-like protein	P55786; E9PLK3; A6NEC2; E9PP11	0	0	2	3	0	0	2	3	3.9	3.9	103.28	6.3039	6	0	0	6286400	2867400
NPM3	Nucleoplasmin-3	O75607	0	0	1	1	0	0	1	1	17.4	17.4	19.343	78.287	2	0	0	2858500	4605500
NSF	Vesicle-fusing ATPase	P46459;I3L0N3	0	1	2	1	0	1	2	1	3.1	3.1	82.593	3.2214	3	0	242890	3137100	704400
NSUN2	tRNA (cytosine(34)-C(5))-methyltransferase	Q08J23	0	0	2	2	0	0	2	2	3.9	3.9	86.47	7.1512	3	0	0	4302800	3116100
NTPCR	Cancer-related nucleoside-triphosphatase	Q9BSD7; Q5TDF0	0	0	3	3	0	0	3	3	24.2	24.2	20.713	46.651	7	0	0	9586100	9090000
NUDT4; NUDT11; NUDT10	Diphosphoinositol polyphosphate phosphohydrolase 2;Diphosphoinositol polyphosphate phosphohydrolase 3-beta;Diphosphoinositol polyphosphate phosphohydrolase 3-alpha	Q9NZJ9; A0A024RBG1;Q96G61;Q8NFP7;F8VRL4;F8VRR0;A0A0C4DGJ4	1	1	2	1	1	1	2	1	16.1	16.1	20.306	4.2509	4	924130	202910	3572500	1678900
NUMA1	Nuclear mitotic apparatus protein 1	Q14980; A0A087WY61;F5H4J1	6	9	15	5	6	9	15	5	10.9	10.9	238.26	156.06	21	4290200	9733800	39090000	3922700
NUP210	Nuclear pore membrane glycoprotein 210	Q8TEM1	2	1	4	1	2	1	4	1	2.2	2.2	205.11	6.9422	7	1101900	802310	5213600	883660
NUP93	Nuclear pore complex protein Nup93	Q8N1F7; H3BVG0	5	2	7	9	5	2	7	9	11.4	11.4	93.487	21.365	17	3080600	603760	28409000	17032000
OAT	Ornithine aminotransferase, mitochondrial;Ornithine aminotransferase, hepatic form;Ornithine	P04181	0	0	3	1	0	0	3	1	8	8	48.534	5.6979	4	0	0	9682200	1467100

	aminotransferase, renal form																		
OGFR	Opioid growth factor receptor	Q9NZT2; A0A087X028;A0A0A0MRN5	1	1	1	2	1	1	1	2	4.1	4.1	73.324	2.819	4	42968000	35908000	10607000	2222100
OTUB1	Ubiquitin thioesterase OTUB1	Q96FW1;F5GYN4;J3KR44; F5H3F0; F5H6Q1; F5GYJ8	0	0	2	2	0	0	2	2	12.2	12.2	31.284	3.3053	1	0	0	3754700	1230800
OXSR1	Serine/threonine-protein kinase OSR1	O95747; C9JIG9	0	0	3	3	0	0	3	3	11.4	11.4	58.022	10.242	4	0	0	12809000	3444600
P4HB	Protein disulfide-isomerase	P07237; H7BZ94; H0Y3Z3	2	8	21	4	2	8	21	4	51	51	57.116	266.39	38	3949700	8367200	177330000	7044600
PA2G4	Proliferation-associated protein 2G4	Q9UQ80; F8VR77; H0YIN7; F8W0A3	0	0	2	0	0	0	2	0	7.1	7.1	43.786	7.0894	2	0	0	8113900	0
PABPC1	Polyadenylate-binding protein 1	P11940; A0A087WTT1;E7EQV3;E7ERJ7;H0YAR2	21	20	18	17	14	14	13	13	31.6	20.8	70.67	277.13	170	519540000	378980000	499810000	232330000
PABPC4	Polyadenylate-binding protein 4;Polyadenylate-binding protein	Q13310; B1ANR0; H0Y5F5	14	12	11	9	7	6	6	5	23.4	12.7	70.782	16.441	25	33768000	22980000	27807000	9010900
PARK7	Protein deglycase DJ-1	Q99497; K7ELW0; K7EN27	2	1	1	3	2	1	1	3	20.1	20.1	19.891	7.0947	5	5412800	923320	3217400	4824900
PARP1	Poly [ADP-ribose] polymerase 1	P09874	1	2	6	1	1	2	6	1	7.5	7.5	113.08	33.586	8	309370	2066500	39273000	3165600
PC	Pyruvate carboxylase, mitochondrial	P11498; A0A494C016	47	46	50	48	47	46	50	48	63.3	63.3	129.63	323.31	929	2822800000	2475800000	6866200000	3946700000
PCBP1	Poly(rC)-binding protein 1	Q15365	5	4	7	6	3	2	5	4	33.7	25	37.497	103.52	46	42465000	26782000	176910000	102110000
PCBP2;PCBP3	Poly(rC)-binding protein 2;Poly(rC)-binding protein 3	Q15366; F8W0G4;F8VXH9;H3BRU6;F8VZX2; P57721; F8W1G6;J3QT27; E9PFP8	4	4	4	4	2	2	2	2	15.9	7.4	38.58	5.3936	11	14475000	9456200	39843000	23700000
PCCA	Propionyl-CoA carboxylase alpha chain, mitochondrial	P05165; A0A1B0GU58;A0A1B0GUX9;A0A1B0GWI4; A0A1B0GWA1	4	3	40	41	4	3	40	41	64	64	80.058	323.31	262	18797000	14691000	3033500000	1293600000
PCCB	Propionyl-CoA carboxylase beta chain, mitochondrial	P05166; E9PDR0; C9JQS9; E7EX59;	1	1	25	23	1	1	25	23	61.4	61.4	58.215	323.31	129	2995900	6782000	3051500000	1106400000

PCMT1	Protein-L-isoaspartate(D-aspartate) O-methyltransferase;Protein-L-isoaspartate O-methyltransferase	E7ETT1; E7ETT4; E7EUY3; E9PEC3; F8WBI9 P22061; A0A0A0MRJ6;F6S8N6;A0A3F2YNX8	12	13	14	12	12	13	14	12	70.5	70.5	24.636	323.31	152	1459000000	969960000	1689100000	801870000
PCNA	Proliferating cell nuclear antigen	P12004	5	4	6	7	5	4	6	7	39.1	39.1	28.768	323.31	32	16003000	8931600	88476000	67681000
PDCD6	Programmed cell death protein 6	O75340; A0A024QZ42;H0Y9X3;A0A087WZ38	1	1	2	2	1	1	2	2	12.6	12.6	21.868	5.4934	5	720880	1772600	12428000	11180000
PDCD6IP	Programmed cell death 6-interacting protein	Q8WUM4	14	14	26	25	14	14	26	25	40.8	40.8	96.022	162.31	69	29318000	11970000	184250000	93978000
PDHB	Pyruvate dehydrogenase E1 component subunit beta, mitochondrial	P11177; F8WF02; C9J634	1	0	3	3	1	0	3	3	12	12	39.233	18.88	5	949760	0	15745000	8089700
PDIA3	Protein disulfide-isomerase A3	P30101	3	3	8	0	3	3	6	0	21.6	17	56.782	16.087	8	2321300	1667900	41921000	0
PDIA6	Protein disulfide-isomerase A6	Q15084	5	10	13	14	5	10	13	14	46.6	46.6	48.121	178.78	54	26069000	41380000	267890000	151230000
PDK1	Pyruvate dehydrogenase (acetyl-transferring) kinase isozyme 1, mitochondrial	Q15118	10	6	14	11	10	6	14	11	49.5	49.5	49.244	212.34	66	50413000	26606000	223410000	134110000
PDS5A	Sister chromatid cohesion protein PDS5 homolog A	Q29RF7	1	0	5	4	1	0	5	4	7.2	7.2	150.83	27.04	8	557390	0	13497000	8703600
PEBP1	Phosphatidylethanolamine-binding protein 1;Hippocampal cholinergic neurostimulating peptide	P30086	2	1	1	0	2	1	1	0	8.6	8.6	21.057	1.6818	2	5514900	1004200	1085300	0
PES1	Pescadillo homolog	O00541; B5MCF9; B3KXD6	0	0	2	0	0	0	2	0	4.4	4.4	68.002	2.0981	2	0	0	6127500	0
PFKL	ATP-dependent 6-phosphofructokinase, liver type	P17858	6	1	13	12	5	1	12	11	22.2	19.7	85.018	118.62	27	10749000	1565100	72154000	27519000
PFKP	ATP-dependent 6-phosphofructokinase, platelet type	Q01813; B1APP6	2	1	6	4	1	1	5	3	9.6	7.1	85.595	24.673	9	797920	64922	16629000	2806900
PFN1	Profilin-1	P07737;I3L3D5	2	1	3	0	2	1	3	0	30	30	15.054	9.9115	5	5290800	1348100	34870000	0
PGAM1; PGAM2	Phosphoglycerate mutase 1;Phosphoglycerate mutase 2	P18669; P15259	1	1	7	1	1	1	7	1	31.9	31.9	28.804	66.834	21	38188000	19428000	146350000	26810000
PGAM5	Serine/threonine-protein phosphatase PGAM5, mitochondrial	Q96HS1	1	2	4	4	1	2	4	4	13.1	13.1	32.004	6.2783	8	4282700	738620	18056000	5383000

PGD	6-phosphogluconate dehydrogenase, decarboxylating	P52209; K7EMN2;K7EM49;K7EPF6	0	0	2	0	0	0	2	0	5.6	5.6	53.139	3.1762	2	0	0	7732900	0
PGK1	Phosphoglycerate kinase 1	P00558	1	1	8	2	1	1	8	2	32.1	32.1	44.614	112.45	21	0	1028100	124330000	1601300
PGM1	Phosphoglucomutase-1	P36871; A0A3B3ITK7	0	0	2	0	0	0	2	0	7.1	7.1	61.448	3.5815	2	0	0	5413500	0
PGM2	Phosphoglucomutase-2	Q96G03; E9PD70; E7ENQ8	3	1	3	0	3	1	3	0	5.4	5.4	68.283	9.3528	6	5285700	975210	7248700	0
PHB	Prohibitin	P35232; C9JW96; C9JZ20; E7ESE2; E9PCW0	10	9	11	12	10	9	11	12	52.9	52.9	29.804	78.572	63	70424000	36138000	176530000	170300000
PHB2	Prohibitin-2	Q99623; F5GY37; J3KPX7; F5GWA7;F5H3X6	9	10	12	12	9	10	12	12	45.5	45.5	33.296	105.04	60	65314000	46603000	354600000	161700000
PHGDH	D-3-phosphoglycerate dehydrogenase	O43175; A0A286YF22;A0A2C9F2M7;A0A286YFA2; A0A286YFL2	3	4	12	9	3	4	12	9	34.3	34.3	56.65	98.882	38	7871800	6815500	130120000	65215000
PIGR	Polymeric immunoglobulin receptor;Secretory component	P01833	17	16	14	10	17	16	14	10	27.9	27.9	83.283	323.31	76	149880000	179240000	81278000	28563000
PIP	Prolactin-inducible protein	P12273	7	7	8	5	7	7	8	5	64.4	64.4	16.572	22.452	60	80129000	52214000	133030000	34675000
PKM	Pyruvate kinase PKM;Pyruvate kinase	P14618; B4DNK4; H3BTN5; H3BR70; H3BQ34	28	26	31	22	28	26	31	22	70.4	70.4	57.936	323.31	254	734570000	445380000	1336400000	214750000
PKP1	Plakophilin-1	Q13835	12	15	19	15	12	15	19	15	34.7	34.7	82.86	203.46	121	172210000	103930000	294740000	100120000
PLCL2;PLCL1	Inactive phospholipase C-like protein 2;Inactive phospholipase C-like protein 1;Phosphoinositide phospholipase C	Q9UPR0;Q15111; H7C276; H3BUD4; A0A0A0MT40	1	1	0	2	1	1	0	2	2.5	2.5	125.86	1.9812	7	14697000	13251000	0	33294000
PLEC	Plectin	Q15149; H0YDN1	2	3	35	2	2	3	34	2	9.5	9.1	531.78	91.024	41	4339500	69253000	231070000	66315000
PLS3;PLS1	Plastin-3;Plastin-1	P13797; A0A0A0MSQ0;Q14651;H7C4N2;C9JAM8	0	0	4	0	0	0	3	0	7.8	6.3	70.81	4.2009	3	0	0	7170700	0
PM20D2	Peptidase M20 domain-containing protein 2	Q8IYS1	0	0	2	0	0	0	2	0	8	8	47.776	4.3137	1	0	0	4713500	0
PNO1	RNA-binding protein PNO1	Q9NRX1;F8WBJ6	2	1	4	4	2	1	4	4	24.6	24.6	27.924	45.353	11	1296500	673680	20163000	6422900

POF1B	Protein POF1B	Q8WVV4	6	5	7	5	6	5	7	5	15.1	15.1	68.064	50.431	39	42268000	17265000	69229000	12095000
POLD2	DNA polymerase delta subunit 2	P49005; F8W8R3; A0A087WWF6;C9JHC7	0	0	3	0	0	0	3	0	9.2	9.2	51.289	28.02	2	0	0	6725000	0
POLD3	DNA polymerase delta subunit 3	Q15054	0	0	3	2	0	0	3	2	9.4	9.4	51.4	8.6915	5	0	0	7044400	4923600
POLDIP2	Polymerase delta-interacting protein 2	Q9Y2S7; B4DEM9	1	0	7	7	1	0	7	7	23.4	23.4	42.033	13.975	9	302240	0	17192000	7581300
PPIA	Peptidyl-prolyl cis-trans isomerase A;Peptidyl-prolyl cis-trans isomerase A, N-terminally processed;Peptidyl-prolyl cis-trans isomerase	P62937; F8WE65; C9J5S7	1	1	3	1	1	1	3	1	27.3	27.3	18.012	18.759	7	1955500	1454400	23865000	2419900
PPL	Periplakin	O60437; K7EKI8; K7EQ71	0	0	7	0	0	0	7	0	4.7	4.7	204.74	20.129	6	0	0	17163000	0
PPP1CA	Serine/threonine-protein phosphatase PP1-alpha catalytic subunit;Serine/threonine-protein phosphatase	P62136; E9PMD7	3	1	6	5	1	0	2	1	23	6.1	37.512	3.2487	3	578930	0	7275900	1604100
PPP1CC	Serine/threonine-protein phosphatase PP1-gamma catalytic subunit;Serine/threonine-protein phosphatase	P36873; F8VYE8; A0A087WYY5;F8VR82;F8W0W8	2	1	6	5	0	0	2	1	24.8	7.4	36.983	48.011	11	1043600	751380	31322000	12474000
PPP2CA; PPP2CB	Serine/threonine-protein phosphatase 2A catalytic subunit alpha isoform;Serine/threonine-protein phosphatase 2A catalytic subunit beta isoform;Serine/threonine-protein phosphatase	P67775; P62714; E5RFI3; E5RHP4; E7ESG8; E5RHC1	2	0	6	4	2	0	6	4	24.3	24.3	35.594	16.855	7	2642200	0	25874000	9859900
PPP2R1A	Serine/threonine-protein phosphatase 2A 65 kDa regulatory subunit A alpha isoform	P30153; B3KQV6; E9PH38	0	0	5	5	0	0	5	5	16	16	65.308	43.967	10	0	0	20533000	9352300
PPP2R2A	Serine/threonine-protein phosphatase 2A 55 kDa regulatory subunit B alpha isoform	P63151; A0A590UJY3;A0A590UJJ1	1	1	7	4	1	1	7	4	17.7	17.7	51.691	24.916	11	981970	234820	23519000	8159600
PRB3;PRB4	Basic salivary proline-rich protein 3;Basic salivary proline-rich protein 4;Protein N1;Glycosylated protein A;Peptide P-D	Q04118; A0A0G2JPD0;A0A0G2JNB4;F5H7C1;E7EXA8;E9PAL0;P10163	1	0	2	0	1	0	2	0	7.4	7.4	30.98	3.6453	2	772320	0	2801300	0

PRDX1	Peroxiredoxin-1	Q06830;A0A0A0MSI0;A0A0A0MRQ5	7	6	8	10	6	4	6	7	55.3	43.7	22.11	26.999	60	1599500000	91552000	2361300000	1345700000
PRDX2	Peroxiredoxin-2	P32119;A6NIW5	4	5	5	5	3	4	4	4	28.8	23.2	21.892	24.085	41	19007000	20326000	62885000	16691000
PRDX4	Peroxiredoxin-4	Q13162;H7C3T4	0	1	4	3	0	0	3	1	18.8	14.4	30.54	5.812	4	0	0	6472900	947920
PRDX5	Peroxiredoxin-5, mitochondrial	P30044	0	1	0	2	0	1	0	2	14.5	14.5	22.086	25.94	3	0	1597400	0	5670300
PRDX6	Peroxiredoxin-6	P30041	2	3	6	4	2	3	6	4	43.3	43.3	25.035	45.701	12	8286800	5075900	52361000	8750800
PRKDC	DNA-dependent protein kinase catalytic subunit	P78527	33	32	74	50	33	32	74	50	22.7	22.7	469.08	323.31	299	131410000	93533000	566540000	231800000
PRPF6	Pre-mRNA-processing factor 6	O94906	4	1	9	6	4	1	9	6	10.8	10.8	106.92	18.199	13	3155900	295800	26359000	9968800
PRPS2	Ribose-phosphate pyrophosphokinase 2	P11908;A6NMS2;D3YTJ7	3	0	4	5	1	0	2	2	23.9	10.1	34.769	37.553	11	2354000	0	18499000	11670000
PRPSAP2	Phosphoribosyl pyrophosphate synthase-associated protein 2	O60256;E7EPA1;I3L331;I3L164;C9JDH0;E7EW35;C9JDU5;C9JJS3;C9K0K7	1	0	2	3	1	0	2	3	8.4	8.4	40.925	7.6165	5	529650	0	4814200	2952200
PSMA4	Proteasome subunit alpha type-4;Proteasome subunit alpha type;Proteasome subunit beta type	P25789;H0YLS6;H0YLC2;H0YMI6;H0YMA1;H0YMZ1;H0YL69;H0YKT8;H0YN18	0	1	2	2	0	1	2	2	7.3	7.3	29.483	3.3923	3	0	1015000	16241000	2316100
PSMA5	Proteasome subunit alpha type-5	P28066	2	2	3	2	2	2	3	2	17	17	26.411	8.6414	12	11982000	6141100	35974000	3652900
PSMA6	Proteasome subunit alpha type-6;Proteasome subunit alpha type	P60900;G3V3U4;G3V3I1;G3V295;G3V5Z7	1	1	3	1	1	1	3	1	14.2	14.2	27.399	5.1084	15	8579300	6586400	17000000	2598300
PSMA7;PSMA8	Proteasome subunit alpha type-7;Proteasome subunit alpha type-7-like	O14818;Q8TAA3;H0Y586;A0A087WYS6	2	1	3	0	2	1	3	0	16.1	16.1	27.887	8.746	6	3871800	704140	11165000	0
PSMB1	Proteasome subunit beta type-1	P20618	1	0	2	0	1	0	2	0	8.7	8.7	26.489	3.8012	6	1863200	0	15013000	0
PSMB5	Proteasome subunit beta type-5	P28074;H0YJM8	2	2	2	1	2	2	2	1	9.5	9.5	28.48	3.8415	14	6727600	6158200	13215000	4472800
PSMC1	26S protease regulatory subunit 4	P62191	0	0	3	1	0	0	3	1	10.2	10.2	49.184	9.3929	3	0	0	8934900	1094200
PSMC2	26S protease regulatory subunit 7	P35998	0	0	3	1	0	0	3	1	8.8	8.8	48.633	20.436	4	0	0	5473400	1030600
PSMC4	26S protease regulatory subunit 6B	P43686	0	0	6	3	0	0	6	3	18.2	18.2	47.366	12.583	8	0	0	16149000	4241700

PSMD12	26S proteasome non-ATPase regulatory subunit 12	O00232	1	0	2	1	1	0	2	1	3.5	3.5	52.904	1.9527	2	545400	0	6268900	180960
PSMD14	26S proteasome non-ATPase regulatory subunit 14	O00487; C9JW37	0	1	2	2	0	1	2	2	15.2	15.2	34.577	3.4918	3	0	633080	5253100	2706400
PSMD2	26S proteasome non-ATPase regulatory subunit 2	Q13200; F8WBS8;H7C1H2;C9JPC0	0	1	4	4	0	1	4	4	5.2	5.2	100.2	6.3489	4	0	553610	6528400	2605100
PSMD3	26S proteasome non-ATPase regulatory subunit 3	O43242	5	5	14	6	5	5	14	6	33.3	33.3	60.977	46.899	23	5080300	2965600	76274000	9504700
PSMD5	26S proteasome non-ATPase regulatory subunit 5	Q16401	0	0	3	0	0	0	3	0	8.5	8.5	56.195	41.375	3	0	0	2499400	0
PTPN6	Tyrosine-protein phosphatase non-receptor type 6	P29350	0	2	14	5	0	2	14	5	32.6	32.6	67.56	82.713	22	0	1776100	68008000	10786000
PUS1	tRNA pseudouridine synthase A, mitochondrial;tRNA pseudouridine synthase	Q9Y606; F5H1S9; G8JLB3	1	0	5	6	1	0	5	6	14.5	14.5	47.47	11.39	8	580550	0	14385000	9022300
PYGL	Glycogen phosphorylase, liver form;Alpha-1,4 glucan phosphorylase	P06737; E9PK47	2	2	11	11	2	2	9	8	18.5	15.2	97.147	37.992	18	1371900	1099400	23600000	18087000
QARS	Glutamine--tRNA ligase	P47897; A0A1B0GVU9;B4DDN1	8	9	33	33	8	9	32	32	56.8	55.7	87.798	323.31	102	11303000	9842900	463170000	261780000
RAB11A; RAB11B	Ras-related protein Rab-11A;Ras-related protein Rab-11B	P62491; Q15907; H3BMH2;H3BSC1	0	2	4	3	0	2	4	3	18.5	18.5	24.393	6.0797	7	0	680450	14707000	5016800
RAB14	Ras-related protein Rab-14	P61106; X6RFL8	0	0	2	0	0	0	2	0	20.9	20.9	23.897	3.1976	2	0	0	3951300	0
RAB1B;RAB1A;RAB1C	Ras-related protein Rab-1B;Ras-related protein Rab-1A;Putative Ras-related protein Rab-1C	Q9H0U4; P62820; E9PLD0; E7END7; Q92928	2	1	5	1	2	1	4	1	28.9	23.4	22.171	11.22	8	4837400	569340	45240000	2690700
RAB27A	Ras-related protein Rab-27A	P51159; H3BN55; H3BUD9; H3BS49; H3BVH7	6	7	7	7	6	7	7	7	38.5	38.5	24.868	21.254	21	35093000	14547000	46147000	28238000
RAB32;RAB29;RAB38	Ras-related protein Rab-32;Ras-related protein Rab-7L1;Ras-related protein Rab-38	Q13637; O14966; P57729	1	2	2	1	1	2	2	1	9.8	9.8	24.997	3.9875	3	950390	993440	4215300	1557900
RAB5B;RAB5C	Ras-related protein Rab-5C;Ras-related protein Rab-5B	P51148; K7ERI8; K7ERQ8; P61020; F8VWU4;F8VVZ0; F8VUA5; F8VVK3; K7ENY4	1	1	4	2	0	0	2	0	22.2	12	23.482	9.2074	6	1892800	1968700	11267000	7963500
RAC2;RAC1	Ras-related C3 botulinum toxin substrate 2;Ras-	P15153; B1AH77; B1AH80;	3	4	6	7	3	4	6	7	37	37	21.429	14.634	11	6504900	7018200	36568000	20092000

	related C3 botulinum toxin substrate 1	B1AH78; P63000																
RAD23B	UV excision repair protein RAD23 homolog B	P54727; Q5W0S4;H0Y579; Q5W0S5	0	1	2	1	0	1	2	1	9.3	9.3	43.171	27.7	3	0	546450	8981700	1397900
RAD50	DNA repair protein RAD50	Q92878; A0A1W2PQ90;A0A494BZX8;A0A494C1B7	0	1	2	2	0	1	2	2	1.8	1.8	153.89	2.8213	3	0	625240	5076300	1681700
RAN	GTP-binding nuclear protein Ran	P62826; B5MDF5; J3KQE5; F5H018; H0YFC6	7	7	8	8	7	7	8	8	41.2	41.2	24.423	86.809	52	59481000	34109000	278440000	170060000
RANBP1	Ran-specific GTPase-activating protein	P43487; C9JJ34; C9JDM3; F6WQW2	2	1	1	2	2	1	1	2	27.9	27.9	23.31	11.206	5	2654800	1065000	4925000	4137400
RAP1B;RAP1A	Ras-related protein Rap-1b;Ras-related protein Rap-1A;Ras-related protein Rap-1b-like protein	P61224; P62834; F5H500; A0A075B6Q0;F5H004;F5H6R7;E7ESV4;F5H4H0;F5H0B7;F5GX62;F5H491;F5GWU8;F5H077;F5GYH7;F5H7Y6;A6NIZ1;F5GYB5	1	1	3	3	1	1	3	3	17.9	17.9	20.825	5.6231	4	1812900	1027300	14117000	7245900
RAP1GAP	Rap1 GTPase-activating protein 1	P47736; Q5T3T0; Q5T3T1; F2Z357; A0A0A0MQY8;X6R8W7	2	2	2	2	2	2	2	2	2.1	2.1	73.361	1.9092	7	30936000	11244000	5854800	10768000
RARS	Arginine--tRNA ligase, cytoplasmic	P54136	4	1	33	34	4	1	33	34	61.8	61.8	75.378	323.31	114	4001400	12721000	474240000	337310000
RASAL3	RAS protein activator like-3	Q86YV0	1	1	2	2	1	1	2	2	2.7	2.7	111.9	8.1923	4	130580	183980	2704600	1791500
RBBP4	Histone-binding protein RBBP4	Q09028; H0YF10	3	1	5	3	1	1	3	1	18.4	13.4	47.655	63.967	7	4509000	888400	27183000	12127000
RBBP7	Histone-binding protein RBBP7	Q16576; E9PC52	3	0	5	4	1	0	3	2	20.7	15.8	47.82	14.583	5	955990	0	11571000	3811400
RBM14	RNA-binding protein 14	Q96PK6; A0A0A6YYI9;F8WDX3;A0A0A0MSL8;B8ZZ74	3	2	2	3	3	2	2	3	5.7	5.7	69.491	8.7301	9	4125300	2795500	23142000	10255000

RBM25	RNA-binding protein 25	P49756	0	0	3	1	0	0	3	1	4.2	4.2	100.18	5.264	3	0	0	3152700	300670
RBM39	RNA-binding protein 39	Q14498; H0Y4X3; A0A0U1RQW2;A0A0U1RQH7;G3XAC6	0	1	3	3	0	1	3	3	7.2	7.2	59.379	15.411	7	0	493920	8435300	2745200
RCC2	Protein RCC2	Q9P258	1	1	4	1	1	1	4	1	11.3	11.3	56.084	6.8032	7	0	1184400	17113000	0
RFC2	Replication factor C subunit 2	P35250; H7C5P4; A0A087WVY3	2	1	3	2	2	1	3	2	9.6	9.6	39.157	4.3671	5	1422300	770770	13277000	4515000
RFC3	Replication factor C subunit 3	P40938; A0A087X270	2	0	2	2	2	0	2	2	7.9	7.9	40.556	132.87	5	2187100	0	11289000	6375400
RFC4	Replication factor C subunit 4	P35249; C9JZI1;C9JXZ7;C9JTT7;C9J8M3	2	0	5	4	2	0	5	4	17.4	17.4	39.681	7.8151	8	980420	0	9823600	4330900
RFC5	Replication factor C subunit 5	P40937	2	0	4	5	2	0	4	5	18.8	18.8	38.496	8.4013	7	3223300	0	18151000	10466000
RNASE7	Ribonuclease 7	Q9H1E1	1	2	2	1	1	2	2	1	16	16	17.419	3.1032	15	10190000	3511300	16881000	5391700
RNH1	Ribonuclease inhibitor	P13489; E9PMJ3; E9PLZ3; E9PIM9; H0YCR7	0	1	4	0	0	1	4	0	11.9	11.9	49.973	68.19	4	0	677380	19089000	0
RPA1	Replication protein A 70 kDa DNA-binding subunit;Replication protein A 70 kDa DNA-binding subunit, N-terminally processed	P27694	24	21	23	23	24	21	23	23	52.8	52.8	68.137	323.31	134	593260000	504780000	601690000	336910000
RPA2	Replication protein A 32 kDa subunit	P15927; Q5TEJ7	6	6	7	7	6	6	7	7	50	50	29.247	131.98	40	106970000	56266000	128610000	104340000
RPL10A	60S ribosomal protein L10a	P62906	7	6	7	6	7	6	7	6	33.6	33.6	24.831	18.192	28	72234000	33169000	114370000	51408000
RPL11	60S ribosomal protein L11	P62913; Q5VVC8	5	4	6	6	5	4	6	6	32	32	20.252	89.057	26	94272000	34207000	233520000	187240000
RPL12	60S ribosomal protein L12	P30050	3	5	5	5	3	5	5	5	49.1	49.1	17.818	228.04	21	7873300	24847000	50938000	53702000
RPL13	60S ribosomal protein L13	P26373; J3QSB4	2	2	3	3	2	2	3	3	14.2	14.2	24.261	47.614	18	5726000	2607400	79213000	20504000
RPL13A	60S ribosomal protein L13a	P40429; M0QYS1	3	3	3	3	3	3	3	3	16.7	16.7	23.577	124.97	18	41130000	22116000	113340000	32937000
RPL14	60S ribosomal protein L14	P50914; E7EPB3	0	3	3	3	0	3	3	3	16.3	16.3	23.432	21.386	14	0	12495000	142070000	67717000
RPL15	60S ribosomal protein L15	P61313; E7EX53; A0A2R8Y738;A0A2R8YEM3;E7EQV9;E7ENU7	0	1	2	0	0	1	2	0	7.8	7.8	24.146	2.9441	3	0	3599300	11925000	0

RPL17;RPL17-C18orf32	60S ribosomal protein L17	P18621;A0A087WXM6;J3QQT2;J3KRX5;A0A0A6YYL6;J3KRB3;A0A087WWH0;J3QS96;J3QLC8;A0A0A0MRF8;A0A087WY81	4	4	4	4	4	4	4	4	32.1	32.1	21.397	108.22	18	17856000	20708000	78535000	60535000
RPL18	60S ribosomal protein L18	Q07020;G3V203;J3QQ67;H0YHA7;F8VYV2;A0A075B7A0;F8VUA6	6	7	7	7	6	7	7	7	35.1	35.1	21.634	66.765	88	246050000	453810000	524280000	249340000
RPL18A	60S ribosomal protein L18a	Q02543;M0R3D6;M0R1A7;M0R117;M0R0P7	2	3	5	5	2	3	5	5	30.1	30.1	20.762	95.789	33	32495000	30162000	162390000	59606000
RPL19	60S ribosomal protein L19	P84098;J3QR09;J3KTE4	3	2	4	3	3	2	4	3	18.4	18.4	23.466	84.173	32	42412000	15071000	167250000	40328000
RPL22	60S ribosomal protein L22	P35268;K7ERI7;K7EJT5;K7EP65;K7EKS7;K7ELC4;K7EMH1	2	3	3	3	2	3	3	3	30.5	30.5	14.787	13.627	12	5316400	21831000	25466000	71855000
RPL23	60S ribosomal protein L23	P62829;C9JD32;B9ZVP7;J3KT29	0	2	2	2	0	2	2	2	25	25	14.865	44.946	5	0	9116500	6319100	30340000
RPL24	60S ribosomal protein L24	P83731;C9JXB8;C9JNW5	2	3	3	3	2	3	3	3	19.7	19.7	17.779	5.4541	7	3507400	5654200	12009000	10682000
RPL27	60S ribosomal protein L27	P61353;K7ELC7	0	4	3	4	0	4	3	4	43.4	43.4	15.798	16.223	13	0	5502800	23321000	28539000
RPL27A	60S ribosomal protein L27a	P46776;E9PJD9;E9PLL6	0	2	3	2	0	2	3	2	22.3	22.3	16.561	6.5129	7	0	3626900	25351000	7988800
RPL28	60S ribosomal protein L28	P46779;H0YLP6;H0YMF4;H0YKD8	2	2	2	2	2	2	2	2	15.3	15.3	15.747	37.982	18	4946800	7609600	39429000	33732000
RPL3	60S ribosomal protein L3	P39023;H7C422;B5MCW2;G5E9G0;H7C3M2	2	5	9	6	2	5	9	6	22.6	22.6	46.108	29.604	21	2393000	8373500	112540000	30342000
RPL4	60S ribosomal protein L4	P36578;H3BM89	5	5	9	8	5	5	9	8	21.3	21.3	47.697	39.723	48	34241000	36359000	178730000	62794000
RPL5	60S ribosomal protein L5	P46777;A0A2R8	2	0	5	5	2	0	5	5	20.2	20.2	34.362	13.437	12	3179900	0	69097000	21472000

		Y6J3;Q5T7N0																	
RPL6	60S ribosomal protein L6	Q02878	5	7	9	7	5	7	9	7	34.7	34.7	32.728	115.9	32	15029000	27234000	108480000	25148000
RPL7	60S ribosomal protein L7	P18124;A8MUD9	5	10	11	12	5	10	11	12	44	44	29.225	82.266	64	45653000	101360000	363330000	155710000
RPL7A	60S ribosomal protein L7a	P62424;Q5T8U2	4	6	9	7	4	6	9	7	36.1	36.1	29.995	36.098	24	10181000	14347000	75776000	26327000
RPL8	60S ribosomal protein L8	P62917;E9PKZ0;E9PP36;G3V1A1;E9PKU4	2	2	2	2	2	2	2	2	10.5	10.5	28.024	12.699	13	9036400	10638000	49410000	21811000
RPL9	60S ribosomal protein L9	P32969;D6RAN4;A0A2R8Y5Y7;H0Y9V9	6	6	8	7	6	6	8	7	65.1	65.1	21.863	208.03	39	33019000	37264000	214150000	230990000
RPLP0;RPLP0P6	60S acidic ribosomal protein P0;60S acidic ribosomal protein P0-like	P05388;F8VWS0;F8VU65;F8VW21;Q8NHW5;F8VPE8;G3V210;F8VZS0;F8VQY6;F8VRK7	9	8	9	7	9	8	9	7	50.2	50.2	34.273	108.09	33	70874000	87439000	162800000	108910000
RPN1	Dolichyl-diphosphooligosaccharide--protein glycosyltransferase subunit 1	P04843;B7Z4L4	13	8	13	8	13	8	13	8	40.9	40.9	68.569	265.52	41	34218000	15461000	116010000	31341000
RPN2	Dolichyl-diphosphooligosaccharide--protein glycosyltransferase subunit 2	P04844	3	0	8	4	3	0	8	4	22	22	69.283	93.53	10	3696700	0	44421000	5555200
RPS10	40S ribosomal protein S10	P46783;A0A2R8Y6L3;F6U211;A0A2R8Y7H1;A0A2R8YFH6;A0A1W2PQS6;Q9NQ39	0	0	3	3	0	0	3	3	23.6	23.6	18.898	12.104	5	0	0	33352000	6139500
RPS11	40S ribosomal protein S11	P62280;M0QZC5;M0R1H5	6	5	8	7	6	5	8	7	46.2	46.2	18.431	96.307	25	19476000	17993000	222910000	48474000
RPS13	40S ribosomal protein S13	P62277;J3KMX5	1	4	5	5	1	4	5	5	31.1	31.1	17.222	11.369	14	712680	6685900	42109000	69582000
RPS14	40S ribosomal protein S14	P62263;A0A2R8Y811;E5RH77	2	3	3	4	2	3	3	4	36.4	36.4	16.273	21.895	17	4093200	7470300	16580000	70786000
RPS15	40S ribosomal protein S15	P62841;S4R456;K7EM56;A0A0B4J2B4;K7E	0	1	2	2	0	1	2	2	9	9	17.04	1.8114	2	0	102480	4278500	800520

		J78;S4R417;K7EQJ5;K7ELC2																
RPS15A	40S ribosomal protein S15a	P62244;I3L3P7;I3L246;H3BN98	1	3	2	4	1	3	2	4	29.2	29.2	14.839	9.4081	9	3219000	25873000	10320000	46310000
RPS16	40S ribosomal protein S16	P62249; M0R210; M0R3H0; M0QX76; M0R1M5;Q6IPX4	0	5	5	6	0	5	5	6	40.4	40.4	16.445	17.923	22	0	24030000	45598000	166550000
RPS18	40S ribosomal protein S18	P62269	1	4	7	7	1	4	7	7	38.8	38.8	17.718	22.039	23	12574000	11007000	44078000	82661000
RPS2	40S ribosomal protein S2	P15880; E9PQD7; H0YEN5; E9PMM9;E9PPT0	1	4	10	9	1	4	10	9	35.2	35.2	31.324	36.638	23	740040	5563800	124090000	47828000
RPS20	40S ribosomal protein S20	P60866; E5RJX2; G3XAN0	1	3	3	4	1	3	3	4	26.1	26.1	13.373	11.83	21	6474600	21829000	67343000	147180000
RPS23	40S ribosomal protein S23	P62266; D6RD47	1	1	2	2	1	1	2	2	16.1	16.1	15.807	4.6402	9	1061300	1119200	12089000	7660400
RPS24	40S ribosomal protein S24	P62847; E7ETK0; A0A2R8Y849;A0A2R8YD14;A0A2R8Y8A0	0	0	2	2	0	0	2	2	15	15	15.423	5.6661	2	0	0	5254300	13805000
RPS3	40S ribosomal protein S3	P23396; E9PL09; E9PPU1; H0YEU2; F2Z2S8; H0YCJ7	12	13	18	14	12	13	18	14	70.8	70.8	26.688	124.93	118	215210000	103720000	725630000	408230000
RPS3A	40S ribosomal protein S3a	P61247; D6RG13; D6RAT0; D6RB09; H0Y8L7; E9PFI5; H0Y9Y4; D6RAS7; D6R9B6	5	7	9	8	5	7	9	8	38.3	38.3	29.945	32.705	42	34892000	35758000	249190000	130330000
RPS4X	40S ribosomal protein S4, X isoform	P62701	10	10	12	11	10	10	12	11	45.6	45.6	29.597	151.96	77	91089000	50368000	471410000	148930000
RPS5	40S ribosomal protein S5	P46782; M0QZN2;M0R0F0;M0R0R2	4	6	7	6	4	6	7	6	27.5	27.5	22.876	64.594	24	67624000	56751000	236810000	114270000
RPS6	40S ribosomal protein S6	P62753; A2A3R7; A2A3R5	4	4	6	6	4	4	6	6	26.1	26.1	28.68	38.622	32	7783400	27922000	102250000	70911000
RPS6KA1	Ribosomal protein S6 kinase;Ribosomal protein S6 kinase alpha-1	E9PGT3; Q15418	0	2	6	3	0	2	6	3	11	11	81.471	12.557	10	0	412590	18337000	4846000
RPS7	40S ribosomal protein S7	P62081; B5MCP9	3	5	5	5	3	5	5	5	26.8	26.8	22.127	27.607	22	22336000	37185000	180200000	113280000

		;A0A2R8Y623																	
RPS8	40S ribosomal protein S8	P62241; Q5JR95	8	8	9	7	8	8	9	7	46.6	46.6	24.205	219.48	40	60528000	29831000	154780000	140160000
RPS9	40S ribosomal protein S9	P46781; B5MCT8; C9JM19	7	9	9	11	7	9	9	11	40.2	40.2	22.591	42.429	48	106740000	79417000	425950000	341550000
RPSA	40S ribosomal protein SA	P08865; C9J9K3; A0A0C4DG17;F8WD59	2	2	8	8	2	2	8	8	45.1	45.1	32.854	101.87	26	6565900	1852500	77987000	26053000
RSL1D1	Ribosomal L1 domain-containing protein 1	O76021; J3QSV6; I3L3C4;I3L3U9;I3L234	0	1	4	4	0	1	4	4	10.6	10.6	54.972	8.1891	5	0	1590300	10459000	5282400
RTCB	tRNA-splicing ligase RtcB homolog	Q9Y3I0	1	1	2	1	1	1	2	1	5	5	55.21	3.2141	4	0	998010	2961000	0
RUVBL1	RuvB-like 1	Q9Y265; E7ETR0	6	4	8	9	6	4	8	9	36	36	50.227	37.627	18	9088800	5941300	34887000	17386000
RUVBL2	RuvB-like 2	Q9Y230; M0R0Y3	3	4	7	4	3	4	7	4	21.8	21.8	51.156	33.292	16	3689500	7016700	51928000	8335400
S100A11	Protein S100-A11	P31949	1	0	4	0	1	0	4	0	64.8	64.8	11.74	87.81	5	731510	0	28115000	0
S100A14	Protein S100-A14	Q9HCY8	2	1	3	2	2	1	3	2	44.2	44.2	11.662	181.87	15	13791000	8265000	50530000	3997900
S100A16	Protein S100-A16	Q96FQ6	1	2	4	2	1	2	4	2	53.4	53.4	11.801	29.981	21	8428300	10358000	62968000	6326400
S100A7	Protein S100-A7	P31151	5	7	6	7	2	4	3	4	57.4	45.5	11.471	60.562	76	72374000	67594000	392350000	81773000
S100A8	Protein S100-A8	P05109	7	5	7	2	7	5	7	2	44.1	44.1	10.834	114.17	87	200900000	130870000	771970000	117750000
S100A9	Protein S100-A9	P06702	9	9	10	8	9	9	10	8	78.9	78.9	13.242	323.31	202	370650000	200770000	2018000000	242180000
SAR1A;SAR1B	GTP-binding protein SAR1a;GTP-binding protein SAR1b	Q9NR31; Q9Y6B6; H0Y5E8; X1WI22; D6RAA2; Q5SQT8; D6RDB2; D6RD69	1	0	2	2	1	0	2	2	11.6	11.6	22.367	4.0503	3	825840	0	4284100	2543500
SBDS	Ribosome maturation protein SBDS	Q9Y3A5; F8WE72; A0A087X020	1	1	2	2	1	1	2	2	10	10	28.763	4.7129	4	2114000	142400	6067200	3189600
SBSN	Suprabasin	Q6UWP8	8	7	10	9	8	7	10	9	39.3	39.3	60.54	201.85	118	100940000	110460000	168680000	52440000
SCAMP3	Secretory carrier-associated membrane protein 3	O14828	1	1	3	3	1	1	3	3	13	13	38.287	8.1438	4	779690	413480	5735200	2250000
SEC22B	Vesicle-trafficking protein SEC22b	O75396	0	0	3	3	0	0	3	3	15.8	15.8	24.593	10.573	4	0	0	8657000	4706700
SEC61A1	Protein transport protein Sec61 subunit alpha isoform 1	P61619; B4DR61	4	1	6	4	4	1	6	4	16.8	16.8	52.264	27.94	15	7327100	630490	52657000	13472000
SERPINA1	Alpha-1-antitrypsin;Short peptide from AAT	P01009; A0A0G2JRN3;A0	2	5	1	1	2	5	1	1	15.8	15.8	46.736	30.993	6	2495500	19178000	2466700	634010

		A024R6I7																
SERPINA12	Serpin A12	Q8IW75	4	3	4	4	4	3	4	4	9.7	9.7	47.174	8.1492	24	24051000	13048000	41681000	4419500
SERPINA3	Alpha-1-antichymotrypsin;Alpha-1-antichymotrypsin His-Pro-less	P01011; G3V3A0	2	5	3	0	2	5	3	0	16.1	16.1	47.65	44.118	7	1671000	15827000	10364000	0
SERPINB1	Leukocyte elastase inhibitor	P30740	6	8	13	8	5	7	12	7	38.8	36.1	42.741	48.815	45	25905000	17376000	108250000	35894000
SERPINB12	Serpin B12	Q96P63	14	11	15	13	14	11	15	13	54.3	54.3	46.276	323.31	138	240940000	99530000	167110000	105450000
SERPINB13	Serpin B13	Q9UIV8; C9JL93; F8WE70; A0A0A0MQW3	3	0	1	0	3	0	1	0	10.7	10.7	44.276	7.576	3	7472900	0	695820	0
SERPINB2	Plasminogen activator inhibitor 2	P05120; E7ERB5; E7EPJ9	2	1	3	1	2	1	3	1	10.4	10.4	46.596	43.674	18	13290000	6533100	30787000	5424200
SERPINB3	Serpin B3	P29508	17	11	21	12	8	6	11	7	56.9	30.3	44.564	323.31	170	194640000	61492000	829430000	74660000
SERPINB4	Serpin B4	P48594; H0Y5H9; C9JZ65	9	7	16	6	0	2	6	1	47.4	20.8	44.853	147.54	23	0	2124500	228580000	451220
SERPINB5	Serpin B5	P36952; C9JLM5	0	0	6	0	0	0	6	0	20.5	20.5	42.1	14.489	8	0	0	34988000	0
SERPINB6	Serpin B6	P35237; A0A2R8Y6A7;A0A2R8YD12;A0A024QZX5; A0A087X1N8	2	2	2	3	2	2	2	2	14.1	12	42.621	7.5467	6	4093700	5963900	12763000	11787000
SERPINB7	Serpin B7	O75635; C9JM00	0	0	2	0	0	0	2	0	6.1	6.1	42.904	3.179	2	0	0	7458700	0
SET;SETSIP	Protein SET;Protein SETSIP	Q01105; P0DME0;A0A0C4DFV9;A0A087X027	1	0	2	0	1	0	2	0	7.9	7.9	33.488	2.8497	3	0	0	3044500	0
SF3B3	Splicing factor 3B subunit 3	Q15393	1	2	3	2	1	2	3	2	3.9	3.9	135.58	6.6279	6	1326300	2094300	8598200	3310300
SFN	14-3-3 protein sigma	P31947	2	3	12	2	2	1	10	1	44	36.7	27.774	196.39	44	2950700	2287900	515730000	3885900
SFXN1	Sideroflexin-1	Q9H9B4; D6RDG7;S4R2X2;D6RFI0	2	2	5	5	2	2	5	5	23.3	23.3	35.619	35.254	13	2035600	1157600	29480000	12484000
SFXN3	Sideroflexin-3	Q9BWM7;A0A1P0AYU5	1	0	5	2	1	0	5	2	19.3	19.3	35.503	19.648	7	389730	0	5185500	2954500
SLC16A1	Monocarboxylate transporter 1	P53985; Q5T8R3; Q5T8R5	2	1	4	3	2	1	4	3	10	10	53.944	13.54	14	2959200	1203000	19628000	12109000
SLC16A3	Monocarboxylate transporter 4	O15427; J3QQS9; J3QQV2; J3QSC3	3	0	4	4	3	0	4	4	10.1	10.1	49.469	8.4589	9	5046700	0	12249000	5234500

SLC1A5	Neutral amino acid transporter B(0);Amino acid transporter	Q15758; M0R144; M0QX44; M0QXM4	0	0	2	0	0	0	2	0	3.9	3.9	56.598	3.9305	2	0	0	10727000	0
SLC25A1	Tricarboxylate transport protein, mitochondrial	P53007	0	0	3	2	0	0	3	2	10.3	10.3	34.012	4.4836	3	0	0	9934500	2554200
SLC25A11	Mitochondrial 2-oxoglutarate/malate carrier protein	Q02978;I3L1P8	0	0	6	2	0	0	6	2	24.8	24.8	34.061	35.277	7	0	0	28047000	4708400
SLC25A12	Calcium-binding mitochondrial carrier protein Aralar1	O75746	2	1	7	3	1	0	3	2	11.9	7.4	74.761	24.102	10	2211100	888590	24427000	3549200
SLC25A24	Calcium-binding mitochondrial carrier protein SCaMC-1	Q6NUK1;A0A3B3IU96	0	0	4	1	0	0	4	1	12.2	12.2	53.354	7.2755	5	0	0	19946000	4047100
SLC25A5	ADP/ATP translocase 2;ADP/ATP translocase 2, N-terminally processed	P05141	10	9	12	12	4	3	6	6	35.2	19.5	32.852	48.136	116	270490000	112730000	1160700000	477670000
SLC25A6;SLC25A4	ADP/ATP translocase 3;ADP/ATP translocase 3, N-terminally processed;ADP/ATP translocase 1	P12236; P12235	7	7	11	9	1	1	5	3	44.3	25.5	32.866	9.3346	11	5590500	2253100	48496000	11534000
SLC3A2	4F2 cell-surface antigen heavy chain	P08195; F5GZS6; J3KPF3	3	0	10	8	3	0	10	8	20.2	20.2	67.993	25.422	15	2596300	0	31883000	25301000
SLC7A5	Large neutral amino acids transporter small subunit 1	Q01650	1	0	2	1	1	0	2	1	6.3	6.3	55.01	3.81	3	1390400	0	7366700	1420100
SMARCA5;SMARCA1	SWI/SNF-related matrix-associated actin-dependent regulator of chromatin subfamily A member 5;Probable global transcription activator SNF2L1	O60264; P28370; A0A0A0MRP6;B7ZLQ5	0	0	2	1	0	0	2	1	1.9	1.9	121.9	3.5662	4	0	0	10963000	1531500
SMARCC1	SWI/SNF complex subunit SMARCC1	Q92922	1	0	4	4	1	0	4	4	7.3	7.3	122.87	13.202	7	9110200	0	17999000	7498300
SMARCE1	SWI/SNF-related matrix-associated actin-dependent regulator of chromatin subfamily E member 1	Q969G3; A0A2R8Y765;A0A2R8Y7U4;A0A2U3TZQ7; A0A2R8Y7I9;A0A2R8Y4T4;A0A2R8YES3; A0A2R8Y855;B4DGM3;J3QKS7;J3QR61	0	0	4	2	0	0	4	2	17.3	17.3	46.649	10.451	5	0	0	10225000	2636200
SMC1A	Structural maintenance of chromosomes protein 1A;Structural maintenance of chromosomes protein	Q14683; G8JLG1; A0A6Q8PHC3	1	2	18	16	1	2	18	16	18.2	18.2	143.23	51.699	38	377340	1481400	59479000	40946000

SMC2	Structural maintenance of chromosomes protein 2	O95347	3	3	11	12	3	3	11	12	13.6	13.6	135.65	31.982	23	2233000	2505400	19344000	23349000
SMC3	Structural maintenance of chromosomes protein 3	Q9UQE7	4	2	9	16	4	2	9	16	19.2	19.2	141.54	92.376	29	3172500	1728000	25180000	41575000
SMC4	Structural maintenance of chromosomes protein 4;Structural maintenance of chromosomes protein	Q9NTJ3; E9PD53	4	7	15	10	4	7	15	10	13.6	13.6	147.18	76.458	25	3148200	3270300	45290000	17105000
SNRNP200	U5 small nuclear ribonucleoprotein 200 kDa helicase	O75643	1	1	3	1	1	1	3	1	1.6	1.6	244.5	5.6536	3	258280	103380	4507700	387140
SNRPA1	U2 small nuclear ribonucleoprotein A	P09661; H0YLR3; H0YMA0	0	0	2	2	0	0	2	2	9.8	9.8	28.415	5.0755	3	0	0	2904400	2239700
SNTB1	Beta-1-syntrophin	Q13884; A0A3B3ITC2;E5RIX7	1	0	2	2	1	0	2	2	7.4	7.4	58.06	4.2848	4	544270	0	3488000	2593000
SNX2	Sorting nexin-2	O60749	0	2	7	4	0	2	7	4	17.7	17.7	58.47	38.009	11	0	763730	20463000	8026200
SORD	Sorbitol dehydrogenase	Q00796; A0A6I8PIS1;H0YKB3	3	0	3	3	3	0	3	3	16	16	38.324	4.4608	5	4522400	0	7619200	6054700
SPCS3	Signal peptidase complex subunit 3	P61009	1	0	2	0	1	0	2	0	11.7	11.7	20.313	3.8783	3	2063200	0	20259000	0
SPG20	Spartin	Q8N0X7	3	1	4	3	3	1	4	3	9.6	9.6	72.832	21.303	8	3931500	2701300	10753000	5744000
SPRR1A	Cornifin-A	P35321	2	3	4	3	1	1	2	1	69.7	36	9.8774	197.65	33	20252000	15961000	56146000	11445000
SPRR2G	Small proline-rich protein 2G	Q9BYE4	3	1	2	3	2	0	1	1	78.1	49.3	8.1576	28.207	3	9028200	0	626900	373240
SPTAN1	Spectrin alpha chain, non-erythrocytic 1	Q13813; A0A0D9SF54;A0A0D9SGF6	13	2	5	0	13	2	5	0	7.3	7.3	284.54	26.804	17	16000000	701330	9392300	0
SPTBN1	Spectrin beta chain, non-erythrocytic 1	Q01082; A0A087WUZ3	10	2	1	0	9	2	1	0	5.4	5.1	274.61	37.591	10	14205000	552200	386490	0
SPTBN2	Spectrin beta chain, non-erythrocytic 2	O15020	1	0	2	0	0	0	2	0	1.3	1	271.32	2.9634	2	0	0	2134600	0
SQRDL	Sulfide:quinone oxidoreductase, mitochondrial	Q9Y6N5; H3BNX3	8	10	16	11	8	10	16	11	40.4	40.4	49.96	138.58	66	36729000	21237000	224890000	77427000
SRM	Spermidine synthase	P19623	4	4	8	9	4	4	8	9	48.3	48.3	33.824	59.411	25	9642400	11340000	66517000	41321000
SRP68	Signal recognition particle subunit SRP68	Q9UHB9	0	0	2	1	0	0	2	1	3.5	3.5	70.729	3.8961	4	0	0	4961400	1803700
SRPR	Signal recognition particle receptor subunit alpha	P08240	0	0	2	0	0	0	2	0	4.7	4.7	69.81	3.5565	2	0	0	1829700	0
SRPRB	Signal recognition particle receptor subunit beta	Q9Y5M8; H7C4H2	0	0	6	4	0	0	6	4	31.7	31.7	29.702	35.198	8	0	0	32185000	11076000

SRRT	Serrate RNA effector molecule homolog	Q9BXP5; H7C3A1; H7C1K0	1	3	4	4	1	3	4	4	3.8	3.8	100.67	5.671	7	659280	1196800	10463000	4922100
SRSF3;SRSF7	Serine/arginine-rich splicing factor 3;Serine/arginine-rich splicing factor 7	P84103; A0A087X2D0;Q16629;A0A0B4J1Z1;C9JAB2	1	0	2	2	1	0	2	2	14	14	19.329	5.2253	3	430940	0	9235500	10046000
SSR4	Translocon-associated protein subunit delta	P51571; A6NLM8	0	2	0	3	0	2	0	3	23.1	23.1	18.998	17.474	5	0	4689800	0	23345000
STAT1	Signal transducer and activator of transcription 1-alpha/beta;Signal transducer and activator of transcription	P42224; A0A669KB56;A0A669KBA4;A0A669KB53; A0A669KB52;J3KPM9;A0A669KBI6;A0A669KB68;E7EPD2;A0A669KB75	0	1	9	10	0	1	9	10	16.7	16.7	87.334	62.284	17	0	546830	32708000	20885000
STK26;STK25	Serine/threonine-protein kinase 26;Serine/threonine-protein kinase 25	Q9P289; O00506; C9JDH9; C9J6L2; C9JCC0; C9J232; H7C279; Q8NBY1; B4E0Y9; C9JJV0	0	0	1	2	0	0	1	2	10.6	10.6	46.528	7.6893	3	0	0	4895600	3092600
STMN1; STMN2	Stathmin;Stathmin-2	P16949; A2A2D0; Q93045; E5RGX5	0	0	0	2	0	0	0	2	14.8	14.8	17.302	5.1699	2	0	0	0	5949400
STOM	Erythrocyte band 7 integral membrane protein	P27105; F8VSL7	0	0	4	2	0	0	4	2	17.4	17.4	31.73	31.074	5	0	0	14562000	2691200
STOML2	Stomatin-like protein 2, mitochondrial	Q9UJZ1; A0A087WYB4	7	5	9	9	7	5	9	9	34.8	34.8	38.534	94.471	33	24862000	24165000	84494000	48541000
SUCLG1	Succinyl-CoA ligase [ADP/GDP-forming] subunit alpha, mitochondrial	P53597; A0A494C0D1	1	0	1	1	1	0	1	1	11.6	11.6	36.249	2.7717	2	634530	0	3657300	18897000
SULT1A1;SULT1A2;SULT1A3;SULT1A4	Sulfotransferase 1A1;Sulfotransferase; Sulfotransferase 1A2;Sulfotransferase 1A3;Sulfotransferase 1A4	P50225; H3BRY5; P50226; P0DMM9;P0DMN0;H3BQX5;A0A0A6YYL2	2	1	5	5	2	1	5	5	21.4	21.4	34.165	22.986	8	1862900	412110	16331000	11767000
SUPT16H	FACT complex subunit SPT16	Q9Y5B9; G3V5A4; G3V401; G3V2X0	0	0	1	1	0	0	1	1	2.7	2.7	119.91	3.9725	2	0	0	674630	1329000

SYK	Tyrosine-protein kinase SYK	P43405	0	0	7	5	0	0	7	5	15	15	72.065	15.691	12	0	0	22672000	6545000
SYNCRIP	Heterogeneous nuclear ribonucleoprotein Q	O60506; B7Z645; F6UXX1	0	0	2	0	0	0	2	0	4.8	4.8	69.602	6.5112	1	0	0	4411600	0
TAGLN2	Transgelin-2	P37802; X6RJP6	1	1	3	2	1	1	3	2	20.6	20.6	22.391	12.99	6	2656900	1035000	13882000	3235600
TARDBP;TDP43	TAR DNA-binding protein 43	Q13148; A0A087WYY0;A0A087X260;G3V162;B1AKP7;A0A087WX29; A0A1W2PNU8;A0A087WW61;A0A087WX67; A0A087WXV3;A0A087WXQ5;A0A0A0N0M3;A0A087WV68	4	3	4	5	4	3	4	5	21	21	44.739	105.24	22	11942000	6435800	28870000	17112000
TARS	Threonine--tRNA ligase, cytoplasmic	P26639	1	0	4	2	1	0	4	2	5.3	5.3	83.434	5.5661	4	893850	0	3888800	2041300
TARSL2	Probable threonine--tRNA ligase 2, cytoplasmic	A2RTX5; B7ZLP8; H0YKB9	1	0	2	2	1	0	2	2	2.9	2.9	92.644	3.0913	3	294520	0	4134300	1553000
TBRG4	Protein TBRG4	Q969Z0; C9J7P5; C9IZN7	0	0	3	0	0	0	3	0	5.4	5.4	70.737	4.3958	3	0	0	5158900	0
TCP1	T-complex protein 1 subunit alpha	P17987; E7ERF2; E7EQR6; F5H282	14	14	27	23	13	13	25	21	63.1	59	60.343	323.31	104	92676000	56374000	476280000	189630000
TF	Serotransferrin	P02787	0	1	8	0	0	1	8	0	13.9	13.9	77.063	61.277	8	0	540880	21321000	0
TFG	Protein TFG	Q92734; C9JTY3; C9JUE0; C9JJP5; A0A6Q8PF51;A0A6Q8PFC4;A0A6Q8PH89; A0A6Q8PH27;A0A6Q8PG00;A0A6Q8PG04; A0A6Q8PGS4;A0A6Q8PF40;A0A6Q8PFY7	1	0	2	0	1	0	2	0	4.5	4.5	43.447	2.1058	2	740560	0	4510100	0
TFRC	Transferrin receptor protein 1;Transferrin	P02786; G3V0E5	4	4	7	7	4	4	7	7	10.7	10.7	84.87	60.847	12	2854200	1917600	17171000	10146000

	receptor protein 1, serum form																		
TGM1	Protein-glutamine gamma-glutamyltransferase K	P22735	12	11	13	11	12	11	13	11	19.3	19.3	89.786	86.008	107	76881000	58594000	156790000	28373000
TGM3	Protein-glutamine gamma-glutamyltransferase E;Protein-glutamine gamma-glutamyltransferase E 50 kDa catalytic chain;Protein-glutamine gamma-glutamyltransferase E 27 kDa non-catalytic chain	Q08188; A0A494C0J7	14	12	10	10	14	12	10	10	27.4	27.4	76.631	305.85	123	131040000	108240000	152350000	112560000
TIMM50	Mitochondrial import inner membrane translocase subunit TIM50	Q3ZCQ8;M0R0C3;M0R2F8;M0R047;M0R003	1	0	4	3	1	0	4	3	17	17	39.646	133.56	8	1054300	0	14330000	8231700
TKT	Transketolase	P29401; A0A0B4J1R6	2	1	5	1	2	1	5	1	13.2	13.2	67.877	21.119	6	1374300	2312700	18770000	659610
TMED10	Transmembrane emp24 domain-containing protein 10	P49755; G3V2K7	0	0	2	1	0	0	2	1	9.6	9.6	24.976	3.54	3	0	0	11334000	1492100
TMEM33	Transmembrane protein 33	P57088; D6RAA6; H0Y8N0	1	1	2	2	1	1	2	2	8.9	8.9	27.978	4.6999	3	4792500	1963500	9812000	2886000
TMOD3	Tropomodulin-3	Q9NYL9; H0YKU1; H0YNJ8	10	5	6	0	10	5	6	0	40.1	40.1	39.594	206.93	17	55764000	5720500	14126000	0
TOMM22	Mitochondrial import receptor subunit TOM22 homolog	Q9NS69	0	2	3	3	0	2	3	3	40.8	40.8	15.521	72.234	6	0	2856800	5891200	26270000
TOMM40	Mitochondrial import receptor subunit TOM40 homolog	O96008; K7EKG4	1	0	4	5	1	0	4	5	20.2	20.2	37.893	21.935	6	363520	0	8461400	6105900
TOP1	DNA topoisomerase 1	P11387	1	2	5	1	1	2	5	1	7.3	7.3	90.725	7.1662	6	262930	3506900	32214000	656560
TOP2A	DNA topoisomerase 2-alpha	P11388	1	2	6	3	0	0	2	0	4.7	2.1	174.38	8.5515	1	0	0	2637900	0
TOP2B	DNA topoisomerase 2-beta;DNA topoisomerase 2	Q02880; E9PCY5	3	3	12	8	2	1	8	5	7.9	5.5	183.26	37.99	24	3329900	3023100	44113000	19647000
TOR1AIP1	Torsin-1A-interacting protein 1	Q5JTV8; A0A0A0MSK5;J3KN66	0	0	2	2	0	0	2	2	6.9	6.9	66.248	5.8989	4	0	0	4896400	1863500
TPI1	Triosephosphate isomerase	P60174; U3KPZ0	6	4	7	5	6	4	7	5	44.2	44.2	26.669	95.627	34	34171000	11349000	111700000	8557600
TPM3	Tropomyosin alpha-3 chain	A0A087WWU8;D6R904;A0A494C0G0;A0A494C0P6	3	3	6	4	1	1	3	2	26.9	16.3	26.42	50.626	28	13318000	9743800	52281000	10468000
TPSAB1; TPSB2	Tryptase alpha/beta-1;Tryptase beta-2	Q15661; P20231; J3QTS8; A0A087	3	2	5	4	3	2	5	4	24	24	30.515	24.958	16	11254000	7534600	36702000	17357000

		WUI4;A0A087X1U0;A0A0C4DGM1																	
TRAP1	Heat shock protein 75 kDa, mitochondrial	Q12931;I3L0K7	0	1	4	3	0	1	4	3	8.2	8.2	80.109	12.917	8	0	835890	44390000	6128900
TRIM28	Transcription intermediary factor 1-beta	Q13263; M0R0K9	0	0	6	4	0	0	6	4	10.3	10.3	88.549	51.216	9	0	0	11457000	6338800
TRIM29	Tripartite motif-containing protein 29	Q14134	0	1	3	0	0	1	3	0	5.1	5.1	65.834	4.8477	3	0	483850	13621000	0
TRIP11	Thyroid receptor-interacting protein 11	Q15643; H0YJ97	30	36	37	4	30	36	37	4	23.6	23.6	227.58	323.31	91	56216000	97541000	108500000	1343700
TRIP13	Pachytene checkpoint protein 2 homolog	Q15645; H0YAL2	0	0	1	3	0	0	1	3	7.9	7.9	48.55	3.6132	4	0	0	3562800	2273000
TRMT10C	Mitochondrial ribonuclease P protein 1	Q7L0Y3; C9JVB6	0	0	1	1	0	0	1	1	6.2	6.2	47.346	10.563	2	0	0	2078700	1970800
TSFM	Elongation factor Ts, mitochondrial;Elongation factor Ts	P43897; C9JG32; C9JT21; F8VPA7; F8VS27	0	1	1	2	0	1	1	2	14.5	14.5	35.39	16.07	3	0	861800	3599700	4012700
TST	Thiosulfate sulfurtransferase	Q16762	0	0	2	2	0	0	2	2	12.5	12.5	33.429	3.6582	2	0	0	6705400	3095300
TTR	Transthyretin	P02766; A0A087WT59	2	6	2	1	2	6	2	1	63.9	63.9	15.887	68.691	11	2846000	20965000	4506900	4472700
TUBA1C; TUBA1B	Tubulin alpha-1C chain	Q9BQE3;F5H5D3;A0A1W2PQM2;F8VVB9	16	14	20	20	0	0	2	2	65.3	9.1	49.895	30.792	8	0	0	25423000	16082000
TUBA4A	Tubulin alpha-4A chain	P68366	16	14	18	18	2	2	3	3	56	9.8	49.924	37.982	12	12956000	10775000	128190000	65212000
TUBB	Tubulin beta chain	P07437; Q5JP53; Q5ST81	18	17	20	22	3	3	4	4	60.8	16	49.67	253.14	126	248390000	221860000	2015100000	983280000
TUBB2B; TUBB2A	Tubulin beta-2B chain;Tubulin beta-2A chain	Q9BVA1; Q13885	13	12	18	17	0	0	3	0	54.6	10.8	49.953	9.548	2	0	0	17572000	0
TUBB6	Tubulin beta-6 chain	Q9BUF5; K7ESM5	6	6	13	9	0	0	3	1	31.6	9.9	49.857	17.34	4	0	0	11188000	919290
TUBB8	Tubulin beta-8 chain	Q3ZCM7;A0A075B736;Q5SQY0	8	7	10	10	1	1	2	2	22.7	7.4	49.775	101.75	7	7221800	1788500	74756000	28349000
TUBG1;TUBG2	Tubulin gamma-1 chain;Tubulin gamma-2 chain;Tubulin gamma chain	P23258; Q9NRH3;K7EKE5;K7EIS0	0	0	2	1	0	0	2	1	6	6	51.169	3.9492	3	0	0	1733200	1258700
TUFM	Elongation factor Tu, mitochondrial	P49411	12	14	20	21	12	14	20	21	56	56	49.541	323.31	162	76544000	57243000	744810000	404800000
TWF2	Twinfilin-2	Q6IBS0; D6RG15	4	2	9	8	4	2	9	8	45.8	45.8	39.548	155.5	23	6827500	1930000	61508000	24938000
TXN	Thioredoxin	P10599	5	5	5	5	5	5	5	5	42.9	42.9	11.737	21.877	67	106720000	76077000	136780000	44240000
TXNDC5	Thioredoxin domain-containing protein 5	Q8NBS9	0	1	2	0	0	1	2	0	6	6	47.628	9.1116	2	0	163300	2421900	0

TYMP	Thymidine phosphorylase	P19971; C9JGI3; A0A494C0L6	1	1	9	0	1	1	9	0	29.5	29.5	49.955	46.075	8	20876000	246510	34613000	0
U2AF1;U2AF1L4	Splicing factor U2AF 35 kDa subunit;Splicing factor U2AF 26 kDa subunit	Q01081; P0DN76; Q8WU68;K7EJH3; A0A1B0GW87;K7EJM7;M0QYK5; M0R2N4	0	0	2	2	0	0	2	2	8.3	8.3	27.872	2.9727	3	0	0	10818000	3096400
UBA1	Ubiquitin-like modifier-activating enzyme 1	P22314; Q5JRS1; Q5JRS3; Q5JRS2; Q5JRR9; Q5JRS0; Q5JRR6	0	0	2	0	0	0	2	0	2.2	2.2	117.85	3.2004	2	0	0	5789500	0
UBA2	SUMO-activating enzyme subunit 2	Q9UBT2; K7EPL2	0	0	6	5	0	0	6	5	13.8	13.8	71.223	22.975	10	0	0	20600000	7789700
UBC;UBB	Polyubiquitin-C;Ubiquitin;Polyubiquitin-B;Ubiquitin;Ubiquitin-60S ribosomal protein L40;Ubiquitin;60S ribosomal protein L40;Ubiquitin-40S ribosomal protein S27a;Ubiquitin;40S ribosomal protein S27a	P0CG48; F5H6Q2; F5H388; B4DV12; F5H265; J3QKN0; P0CG47; F5GYU3; Q5PY61; F5H747; J3QTR3; J3QS39; Q96C32; F5H2Z3; P62987; P62979; F5GXK7; M0R2S1; A0A2R8Y422;M0R1M6;M0R1V7;F5GZ39;J3QSA3	6	6	6	6	6	6	6	6	64.4	64.4	77.038	156.3	135	328870000	276200000	689720000	226810000
UBE2V1;UBE2V2;TMEM189-UBE2V1	Ubiquitin-conjugating enzyme E2 variant 1;Ubiquitin-conjugating enzyme E2 variant 2	Q13404; Q15819; E5RIF1; D6RG00; H0YBX6; I3L0A0	2	0	0	0	2	0	0	0	15	15	16.495	2.5628	2	3778800	0	0	0
UBTF	Nucleolar transcription factor 1	P17480; E9PKP7	0	0	4	0	0	0	4	0	7.1	7.1	89.405	10.411	5	0	0	13680000	0
UFL1	E3 UFM1-protein ligase 1	O94874	0	0	2	0	0	0	2	0	4.5	4.5	89.594	1.7155	2	0	0	37706000	0
UGDH	UDP-glucose 6-dehydrogenase	O60701	0	1	4	2	0	1	4	2	13.2	13.2	55.023	7.6869	6	0	560230	8207100	3410600
UMPS	Uridine 5-monophosphate synthase;Orotate phosphoribosyltransferase;Orotidine 5-	P11172; E9PFD2	0	0	2	2	0	0	2	2	6.2	6.2	52.221	3.0306	3	0	0	2371300	2725800

	phosphate decarboxylase																	
UNC45A	Protein unc-45 homolog A	Q9H3U1; A0A1W2PNX8	0	0	1	1	0	0	1	1	3.1	3.1	103.08	2.8511	2	0	0	1066200	1137000
UPF1	Regulator of nonsense transcripts 1	Q92900	1	1	3	1	1	1	3	1	4.6	4.6	124.34	5.0217	2	202050	382380	2997800	462910
UQCRC2	Cytochrome b-c1 complex subunit 2, mitochondrial	P22695; H3BRG4;H3BSJ9	6	5	10	11	6	5	10	11	46.8	46.8	48.442	143.59	49	17423000	13418000	122850000	80979000
UQCRFS1	Cytochrome b-c1 complex subunit Rieske, mitochondrial;Cytochrome b-c1 complex subunit 11	P47985	1	0	2	3	1	0	2	3	10.9	10.9	29.668	30.975	6	1612100	0	12583000	12620000
VARS	Valine--tRNA ligase	P26640; A0A140T936	1	0	6	2	1	0	6	2	6.9	6.9	140.47	14.269	8	721720	0	15964000	2759400
VCL	Vinculin	P18206	3	3	4	0	3	3	4	0	5.6	5.6	123.8	22.562	8	2331400	2325400	11618000	0
VCP	Transitional endoplasmic reticulum ATPase	P55072	2	0	8	1	2	0	8	1	14.8	14.8	89.321	48.022	10	2599300	0	36373000	192480
VDAC1	Voltage-dependent anion-selective channel protein 1	P21796	1	0	6	4	1	0	6	4	29.7	29.7	30.772	16.777	15	0	0	24863000	13955000
VDAC2	Voltage-dependent anion-selective channel protein 2	P45880; A0A0A0MR02	4	4	7	6	4	4	7	6	35.7	35.7	31.566	108.13	28	11081000	11885000	64793000	36075000
VDAC3	Voltage-dependent anion-selective channel protein 3	Q9Y277; E5RJN6; E5RHZ6	2	2	5	6	2	2	5	6	32.2	32.2	30.658	59.628	19	9900300	7791700	35875000	37052000
VIM	Vimentin	P08670; B0YJC4; B0YJC5	9	6	19	17	8	6	18	16	48.5	46.6	53.651	247.14	69	41572000	20277000	262770000	88876000
VPS13C	Vacuolar protein sorting-associated protein 13C	Q709C8; A0A3B3IT88;A0A3B3IU50	3	2	3	3	3	2	3	3	1.5	1.5	422.39	6.2465	4	2649200	1717000	5242300	1256300
VPS4B	Vacuolar protein sorting-associated protein 4B	O75351	0	0	2	1	0	0	2	1	7.2	7.2	49.301	4.4705	2	0	0	5206200	1122400
XP32	Skin-specific protein 32	Q5T750	5	5	6	5	5	5	6	5	20.8	20.8	26.238	30.226	38	80875000	49768000	192690000	28426000
XPO1	Exportin-1	O14980	5	1	13	12	5	1	13	12	16.6	16.6	123.38	51.158	25	1836000	596980	54149000	25664000
XPO7	Exportin-7	Q9UIA9; E7ESC6	1	1	2	3	1	1	2	3	4.7	4.7	123.91	4.539	4	344790	7110600	30157000	38406000
XPOT	Exportin-T	O43592	1	1	7	7	1	1	7	7	11.3	11.3	109.96	26.183	12	972010	725450	23933000	15701000
XRCC5	X-ray repair cross-complementing protein 5	P13010	14	16	19	15	14	16	19	15	46.9	46.9	82.704	142.61	90	67392000	46540000	234190000	81730000
XRCC6	X-ray repair cross-complementing protein 6	P12956; B1AHC9	22	23	25	22	22	23	25	22	49.3	49.3	69.842	226.89	126	118930000	148660000	213860000	132740000
YARS	Tyrosine--tRNA ligase, cytoplasmic	P54577; A0A6Q8PFX2	10	8	19	22	10	8	19	22	44.9	44.9	59.143	79.445	55	19834000	17639000	222400000	140700000
YBX1;YBX3;YBX2	Nuclease-sensitive element-binding	P67809; P16989;	7	5	7	7	7	5	7	7	41.4	41.4	35.924	297.81	34	137970000	52178000	180160000	93228000

	protein 1;Y-box-binding protein 3;Y-box-binding protein 2	Q9Y2T7; H0Y449																	
YTHDF2	YTH domain-containing family protein 2	Q9Y5A9; S4R3V3; S4R3J8	0	0	1	2	0	0	1	2	4	4	62.333	2.9732	2	0	0	1265100	2210500
YWHAB	14-3-3 protein beta/alpha;14-3-3 protein beta/alpha, N-terminally processed	P31946	1	3	6	3	1	1	4	2	30.1	22.8	28.082	189.03	11	1274500	430320	49321000	1196700
YWHAE	14-3-3 protein epsilon	P62258	0	4	9	3	0	2	7	2	30.2	23.1	29.174	17.266	12	0	1806500	46860000	1215700
YWHAG	14-3-3 protein gamma;14-3-3 protein gamma, N-terminally processed	P61981	1	3	4	2	1	1	2	1	17	9.7	28.302	8.8147	3	149350000	40573000	87090000	103990000
YWHAQ	14-3-3 protein theta	P27348; E9PG15	4	5	10	8	4	3	8	7	42.9	39.6	27.764	261.37	21	11989000	3975100	102210000	26451000
YWHAZ	14-3-3 protein zeta/delta	P63104; E7EX29; B0AZS6; E7ESK7	2	2	9	4	2	0	7	3	46.5	39.2	27.745	294.78	25	7999500	0	202350000	5674800
ZG16B	Zymogen granule protein 16 homolog B	Q96DA0; A0A0C4DGN4;I3L1H9;I3L3X0	1	1	2	0	1	1	2	0	17.8	17.8	22.739	67.921	6	2458700	1475300	12624000	0

Supplementary Table 2. qPCR primers, PCR primers for site-directed mutagenesis, and siRNA sequences

qPCR primer		
Gene	Forward	Reverse
EPRS1	AAGCGGAAAAGGCTCCTAAG	CCCAGTCTTTTCTTTATACTCAGCTT
GAPDH	TGACCACAGTCCATGCCAT	GACGGACACATTGGGGGTAG
PCR primer for site-directed mutagenesis		
EPRS1 S886A	AATTTCTGGTTGGGGCTGAATCCGAACTTTGAGATAAT GGGGG	CCCCCATTATCTCAAAGTTCGGATTCAGCCCCAACCAG AAATT
EPRS1 S999A	TCCTGCTCCACTTGCTGAGAGCCCACCTC	GAGGTGGGCTCTCAGCAAGTGGAGCAGGA
EPRS1 S886D	CCCCCATTATCTCAAAGTTCGGATTCAGACCCAACCAG AAATT	AATTTCTGGTTGGGTCTGAATCCGAACTTTGAGATAAT GGGGG
EPRS1 S999D	TCTAAAAACCAAGGAGGTGGGCTCTCAGATAGTGGAG CAGGAGA	TCTCCTGCTCCACTATCTGAGAGCCCACCTCCTTGGTT TTTAGA
EPRS1 K974A/K977A	TTTGGCCATCATTTTGTTTCTGAGGCGCATTCTGCGCT TCAGATTTATTTTCTTTTTCTTTCTTCTTCTTATCT	AGATAAGAAGAAGAAAGAAAAAGAAAATAAATCTGAAG CGCAGAATGCGCCTCAGAAACAAAATGATGGCCAAA
EPRS1 P978A/K980A	CCTTTGGCCATCATTTTGTGCCTGAGCCGCATTCTGCG CTTCAGAT	ATCTGAAGCGCAGAATGCGGCTCAGGCACAAAATGAT GGCCAAAGG
Rab5B Q79L	GAGATCTGGGACACAGCTGGGCTGGAGCGATATCACA GCTTAGCC	GGCTAAGCTGTGATATCGCTCCAGCCCAGCTGTGTCC CAGATCTC
siRNA sequence		
EPRS1	CAAAGUCAUCAUCAAACAC	
AKT1	Cell Signaling Technology, SignalSilence, #6909	
AKT2	Cell Signaling Technology, SignalSilence, #6407	
Control	Cell Signaling Technology, SignalSilence, #6568	
