# Supplementary material for: Glutamyl-prolyl-tRNA synthetase 1 coordinates early endosomal anti-inflammatory AKT signaling
Source: Nat Commun. 2022 Oct 29;13:6455. doi: 10.1038/s41467-022-34226-4 (PMC9617928; doi:10.1038/s41467-022-34226-4)
Supplement: Supplementary file 3 — Reporting Summary [file 41467_2022_34226_MOESM3_ESM.pdf]

nature portfolio

Corresponding author(s): Myung Hee Kim, Eun-Young Lee

Last updated by author(s): Sep 21, 2022

Reporting Summary

Nature Portfolio wishes to improve the reproducibility of the work that we publish. This form provides structure for consistency and transparency in reporting. For further information on Nature Portfolio policies, see our Editorial Policies and the Editorial Policy Checklist.

Statistics

For all statistical analyses, confirm that the following items are present in the figure legend, table legend, main text, or Methods section.

n/a	Confirmed	
☐	☒	The exact sample size (*n*) for each experimental group/condition, given as a discrete number and unit of measurement
☐	☒	A statement on whether measurements were taken from distinct samples or whether the same sample was measured repeatedly
☐	☒	The statistical test(s) used AND whether they are one- or two-sided<br>*Only common tests should be described solely by name; describe more complex techniques in the Methods section.*
☒	☐	A description of all covariates tested
☒	☐	A description of any assumptions or corrections, such as tests of normality and adjustment for multiple comparisons
☐	☒	A full description of the statistical parameters including central tendency (e.g. means) or other basic estimates (e.g. regression coefficient) AND variation (e.g. standard deviation) or associated estimates of uncertainty (e.g. confidence intervals)
☐	☒	For null hypothesis testing, the test statistic (e.g. *F*, *t*, *r*) with confidence intervals, effect sizes, degrees of freedom and *P* value noted<br>*Give P values as exact values whenever suitable.*
☒	☐	For Bayesian analysis, information on the choice of priors and Markov chain Monte Carlo settings
☒	☐	For hierarchical and complex designs, identification of the appropriate level for tests and full reporting of outcomes
☐	☒	Estimates of effect sizes (e.g. Cohen's *d*, Pearson's *r*), indicating how they were calculated

Our web collection on statistics for biologists contains articles on many of the points above.

Software and code

Policy information about availability of computer code

Data collection	Data collection described in Methods section. Confocal microscopy images were acquired under a Nikon laser scanning confocal microscope (C2plus, A1R). qRT-PCR data were collected with Roche LightCycler96.
Data analysis	NIS-Elements software (AR 4.10.00 64-bit) for confocal microscopy image analysis<br>ImageJ version 1.53k software to analyze the mean pixel density<br>LightCycler 96 software for qRT-PCR data analysis<br>GraphPad Prism 6 for statistical analysis

For manuscripts utilizing custom algorithms or software that are central to the research but not yet described in published literature, software must be made available to editors and reviewers. We strongly encourage code deposition in a community repository (e.g. GitHub). See the Nature Portfolio guidelines for submitting code & software for further information.

Data

Policy information about availability of data

All manuscripts must include a data availability statement. This statement should provide the following information, where applicable:

- Accession codes, unique identifiers, or web links for publicly available datasets
- A description of any restrictions on data availability
- For clinical datasets or third party data, please ensure that the statement adheres to our policy

All data are available within this article and the Supplementary Information files. The mass spectrometry proteomics data generated in this study have been deposited in the ProteomeXchange Consortium via the PRIDE partner repository under accession code PXD036072. Source data are provided with this paper.

Human research participants

Policy information about studies involving human research participants and Sex and Gender in Research.

Reporting on sex and gender	n/a
Population characteristics	n/a
Recruitment	n/a
Ethics oversight	n/a

Note that full information on the approval of the study protocol must also be provided in the manuscript.

Field-specific reporting

Please select the one below that is the best fit for your research. If you are not sure, read the appropriate sections before making your selection.

[x] Life sciences [ ] Behavioural & social sciences [ ] Ecological, evolutionary & environmental sciences

For a reference copy of the document with all sections, see nature.com/documents/nr-reporting-summary-flat.pdf

Life sciences study design

All studies must disclose on these points even when the disclosure is negative.

Sample size	The sample sizes were determined by referring recent papers including in vivo experiments for survival assay. The in vitro sample size was completed according to enable statistical analyses. The sample sizes of all animal experiments were described in each Figure legend.
Data exclusions	No data were excluded from analyses.
Replication	For all experiments, at least two biological replicates were analyzed in at least two independent experiments. All the replicates showed consistent reproducibility.
Randomization	Allocation of cells for experimental procedures was done at random. For animal studies, mice were grouped according to the genotype. Each experiment mice were age- and sex-matched.
Blinding	In general, the investigators were blind at the time of experiment execution and data acquisition. Especially, in vivo experiments in this study were performed by blind test since mice were identified by individual numbering after group allocations. Determination of mice body weights and survivals were considered as objective measures, not subject to bias. For histopathological and microscopical examinations, samples were blinded to ensure unbiased imaging and analyses.

Reporting for specific materials, systems and methods

We require information from authors about some types of materials, experimental systems and methods used in many studies. Here, indicate whether each material, system or method listed is relevant to your study. If you are not sure if a list item applies to your research, read the appropriate section before selecting a response.

Materials & experimental systems

n/a	Involved in the study
	☒ Antibodies
	☒ Eukaryotic cell lines
☒	☐ Palaeontology and archaeology
	☒ Animals and other organisms
☒	☐ Clinical data
☒	☐ Dual use research of concern

Methods

n/a	Involved in the study
☒	☐ ChIP-seq
☒	☐ Flow cytometry
☒	☐ MRI-based neuroimaging

Antibodies

Antibodies used

The following antibodies were used: anti-EPRS1 (Abcam, ab31531; Thermo Fisher Scientific, A303-957A), anti-MARS1 (Abcam, ab50793), anti-AIMP3 (Neomics, NMS-01-0002), and anti-KARS1 (Neomics, NMS-02-0005).
The following antibodies were from Cell Signaling Technology: anti-FLAG (#8146), anti-HA (#2999), anti-Actin-HRP (#12620), anti-AKT (#4691), anti-AKT1 (#2967), anti-AKT2 (#5239), anti-p-AKT Thr308 (#2965), anti-p-AKT Ser473 (#4060), anti-Rab5 (#3547), anti-GSK3β (#12456), anti-p-GSK3β Ser9 (#5558), anti-CREB (#9197), anti-p-CREB Ser133 (#9198), anti-NF-kB p65 (#8242), anti-p-NF-kB p65 Ser536 (#3033), anti-mTOR (#2983), anti-p-mTOR S2448 (#5536), anti-S6K (#2708), anti-p-S6K Thr389 (#9234), anti-ERK1/2 (#4695), anti-ERK1/2 T202/Y204 (#9101), anti-JNK (#9252), anti-p-JNK T183/Y185 (#4668), anti-p38 (#9212), anti-p-p38 T108/Y182 (#4511), anti-RSK1 (#9333), anti-PKC (#59754), and anti-GST (#2622).
The following antibodies were from Santa Cruz Biotechnology: anti-STK26 (sc-376649), anti-SYK (sc-1240), anti-OSR1 (sc-376545), anti-PKA (sc-365615), anti-PKN (sc-393344), anti-BTK (sc-81735), and anti-GFP (sc-9996).
Other antibodies included anti-Strep (IBA, #2-1509-001, GmbH, Germany), Alexa Fluor 594-conjugated anti-rabbit IgG (Invitrogen, A11037), anti-p-EPRS1 Ser990 (Abclon, Korea). Phospho-specific Ser886 and Ser999 antibodies were provided by Prof. Paul L. Fox (Cleveland Clinic Lerner Research Institute).

Validation

All the commercially available antibodies are validated by the manufacturers, and validation data are available at the manufacturers' websites.
In house made anti-EPRS1 phosphorylation antibodies (Ser886, S990, and Ser999) were validated by immunoblotting (described in the paper with references: Nat Immunol 17, 1252-1262, 2016; Mol Cell 35, 164-180, 2009).

Eukaryotic cell lines

Policy information about cell lines and Sex and Gender in Research

Cell line source(s)	293T (ATCC), HeLa (ATCC), U937 (ATCC), and Raw 264.7 (KCLB) cells
Authentication	Independent authentication was not done in the laboratory.
Mycoplasma contamination	All cell lines were free of mycoplasma contaminations.
Commonly misidentified lines (See ICLAC register)	No commonly misidentified cell lines were used in this study.

Animals and other research organisms

Policy information about studies involving animals; ARRIVE guidelines recommended for reporting animal research, and Sex and Gender in Research

Laboratory animals	C57BL/6N mice both wild-type (Eprs1+/+) and EPRS1 knockdown (heterozygous Eprs1+/-), or mice with conditional deletion of EPRS1 in macrophages by LysM promoter driven, Cre recombinase-mediated excision of exons of the EPRS1 gene (Eprs1fl/fl-Lyz2Cre) and LysM-Cre-negative homozygous floxed EPRS1 littermates (Eprs1fl/fl) between the ages of 6-9 weeks were used for this study.
Wild animals	This study did not involve wild animals.
Reporting on sex	Care was taken even disperse sexes between experimental groups.
Field-collected samples	This study did not involve field-collected samples.
Ethics oversight	All animal experiments were approved by the Institutional Animal Use and Care Committee of the Korea Research Institute of Bioscience and Biotechnology, and were performed in accordance with the Guide for the Care and Use of Laboratory Animals (published by the US National Institutes of Health).

Note that full information on the approval of the study protocol must also be provided in the manuscript.
